# A Multi-Strategy Enhanced Bionic-Inspired Secretary Bird Optimization Algorithm for Numerical Optimization and Artistic Image Segmentation

**DOI:** 10.3390/biomimetics11060385

**Published:** 2026-06-01

**Authors:** Xuanqi Yuan, Jinlu Qin, Xiaohan Zhong

**Affiliations:** 1 School of Packaging Design & Art, Hunan University of Technology, Zhuzhou 412007, China; 14565@hut.edu.cn (X.Y.); 14438@hut.edu.cn (J.Q.); 2 School of Design, Shunde Polytechnic University, Foshan 528000, China; 3 Department of Design, Chung-Ang University, Seoul 06974, Republic of Korea

**Keywords:** metaheuristic algorithm, secretary bird optimization algorithm, multilevel threshold image segmentation, numerical optimization, swarm intelligence algorithms

## Abstract

To address the limitations of the original Secretary Bird Optimization Algorithm (SBOA), such as insufficient population diversity, weak local exploitation ability, and a tendency to become trapped in local optima when solving complex optimization problems, this paper proposes a Multi-Strategy Improved Secretary Bird Optimization Algorithm (MISBOA). First, a chaotic elite initialization strategy is introduced to improve the quality and diversity of the initial population. Second, an adaptive spiral Lévy flight strategy is designed to enhance the balance between global exploration and local exploitation during the iterative process. Third, a dynamic neighborhood-guided mutation strategy is incorporated to maintain population diversity and improve convergence accuracy in the later search stage. To validate the effectiveness of the proposed algorithm, MISBOA is comprehensively evaluated on the IEEE CEC2014, CEC2017, and CEC2020 benchmark suites. Experimental results demonstrate that MISBOA achieves superior convergence speed, optimization accuracy, and robustness compared with several representative metaheuristic algorithms. Furthermore, MISBOA is applied to Otsu-based multilevel threshold image segmentation. The segmentation performance is assessed using PSNR, FSIM, SSIM, and visual quality comparisons. The results indicate that MISBOA can generate more accurate and stable segmentation outcomes, demonstrating its strong potential for solving complex numerical optimization and artistic image segmentation problems.

## 1. Introduction

In recent years, driven by the rapid advancements in artificial intelligence, big data, cloud computing, and computer vision technologies, image processing has emerged as one of the most dynamic and practically valuable research directions within the field of information science. Serving as a fundamental basis for image understanding and analysis, image processing techniques are widely applied across a multitude of domains, including medical diagnosis [1], remote sensing [2], intelligent transportation [3], industrial inspection [4], agricultural identification [5], facial recognition [6], and digital media [7]. Particularly against the backdrop of the continuous evolution of deep learning and intelligent perception technologies, there is a growing demand for higher standards regarding image quality, object recognition accuracy, and automated analysis capabilities [8]; this, in turn, has further propelled the sustained development of image processing-related technologies. Among the myriad tasks within image processing, image segmentation stands out as a core research area in computer vision, primarily because it enables the partitioning of an image into distinct regions characterized by similar features, while simultaneously extracting critical information regarding the boundaries, textures, and structural properties of target objects. High-quality image segmentation results not only enhance the accuracy of subsequent feature extraction and object recognition processes but also significantly boost the overall performance of image analysis systems, thereby attracting increasing attention from researchers.

Image segmentation techniques hold immense significance in practical applications. For instance, in medical image analysis, precise image segmentation enables clinicians to rapidly identify lesion areas, thereby enhancing the efficiency of disease diagnosis [9]. In the realm of remote sensing image processing, image segmentation is utilized for land-cover classification, road extraction, and object monitoring [10]. Within the field of industrial inspection, it facilitates product defect detection and quality assessment [11]. Furthermore, in the domains of artistic image processing and digital media, image segmentation is widely applied in tasks such as style transfer, image restoration, object matting, and the creation of visual effects [12]. Compared to natural images, artistic images typically feature more intricate textural information, richer color variations, and more pronounced characteristics of subjective expression; consequently, their segmentation presents a greater challenge. This is particularly true for artistic images, where distinct regions often lack clear boundaries and frequently contain numerous areas characterized by rich detail, similar color tones, or overlapping textures—conditions that render traditional segmentation methods ill-equipped to yield optimal results. Consequently, a key direction in current research involves exploring how to simultaneously enhance the accuracy and stability of artistic image segmentation while faithfully preserving the image’s edge and detailed information.

Currently, researchers have proposed numerous distinct approaches to address the problem of image segmentation, primarily encompassing threshold-based [13], region-based [14], clustering-based [15], edge-detection-based [16], and deep-learning-based methods [17]. Among these, threshold-based segmentation has garnered significant attention due to its simplicity of implementation, computational efficiency, and applicability across a wide variety of image scenarios. In particular, multi-threshold image segmentation methods are capable of effectively extracting information regarding distinct regions within an image, thereby yielding superior segmentation results compared to single-threshold methods [18]. However, as the number of thresholds increases, the image segmentation problem gradually evolves into a complex optimization challenge characterized by high dimensionality, non-linearity, and multi-modality; consequently, traditional exhaustive search methods often suffer from drawbacks such as high computational costs, slow convergence rates, and a susceptibility to becoming trapped in local optima. Therefore, the efficient search for the optimal combination of thresholds has emerged as a critical factor in enhancing image segmentation performance.

To address the aforementioned complex image segmentation problems, researchers have begun exploring the use of optimization algorithms to search for the optimal combination of thresholds, aiming to enhance both the efficiency and accuracy of image segmentation. While traditional optimization methods—such as gradient descent [19], dynamic programming [20], and exhaustive search [21]—can yield satisfactory results when applied to simple, low-dimensional problems, they often suffer from significant drawbacks when confronted with high-dimensional, nonlinear, and multimodal challenges like multi-threshold image segmentation. Specifically, in the context of artistic image segmentation—characterized by intricate textures and rich color variations—the strong coupling relationships among different thresholds make it exceedingly difficult for traditional methods to achieve satisfactory segmentation results within a finite timeframe.

Against this backdrop, metaheuristic optimization algorithms have gradually emerged as a crucial tool for addressing complex image segmentation problems, owing to their robust global search capabilities, minimal parameter requirements, and reduced reliance on specific problem models. Typically, metaheuristic algorithms simulate natural phenomena—such as biological behaviors, collective cooperation, or evolutionary mechanisms—to iteratively update candidate solutions within the search space, thereby facilitating an efficient search for the optimal solution. Compared to traditional optimization methods, metaheuristic algorithms do not require gradient information and impose fewer constraints regarding the continuity and differentiability of the objective function; consequently, they are particularly well-suited for solving complex, multimodal, and non-differentiable problems.

In recent years, an increasing number of researchers have employed heuristic algorithms to solve image segmentation problems. For instance, to address the problem of multi-threshold image segmentation, Ma et al. proposed an improved multi-threshold image segmentation method based on the Whale Optimization Algorithm [22], utilizing inter-class variance (Otsu’s method) as the objective function. Experimental results demonstrate that the proposed RAV-WOA is capable of selecting appropriate thresholds for both grayscale and color images while simultaneously ensuring high efficiency and segmentation quality. To address the image processing task of grayscale multi-level thresholding segmentation, Laith et al. proposed a novel heuristic optimization algorithm that combines the Reptile Search Algorithm with the Salp Swarm Algorithm [23]. This method employs Otsu’s variance classification function to determine the optimal thresholds at each level. Experimental results on COVID-19-related images demonstrate that the performance of the proposed algorithm surpasses that of other heuristic optimization algorithms reported in the literature. To address the challenge of processing Alzheimer’s disease images—which feature complex textures and rich information content—Yu et al. proposed a novel 3D Rényi entropy model that integrates grayscale intensity [24], nonlocal means, and local entropy information. Simulation results demonstrate the model’s outstanding segmentation performance and stability, fully highlighting its immense potential for practical applications in medical image analysis.

The Secretary Bird Optimization Algorithm (SBOA) is a novel population-based meta-heuristic algorithm proposed by Fu et al. in 2024, inspired by the survival behaviors of secretary birds in their natural environment [25]. Experimental evaluations—conducted using the CEC-2017 and CEC-2022 benchmark suites, 12 engineering optimization problems, and a 3D path planning task for unmanned aerial vehicles (UAVs)—demonstrated that SBOA is capable of identifying superior solutions at a faster convergence rate, thereby fully showcasing its immense potential for solving real-world optimization problems. Since its inception, the algorithm has garnered widespread attention within the academic community; numerous researchers have subsequently built upon this algorithm, implementing targeted modifications to address their specific optimization challenges and achieve enhanced results. For instance, to address problems related to wireless sensor network deployment and engineering, Meng et al. improved the SBOA by incorporating a differential cooperative search mechanism [26]. Simulation results indicate that, although the improved algorithm exhibits inconsistent performance in high-dimensional multimodal and complex constrained optimization problems, it performs well in the three specific engineering design and wireless sensor deployment problems it was designed to solve. To enhance power generation efficiency, Qin et al. improved the SBOA by incorporating a feedback regulation mechanism based on incremental PID control [27], a collaborative camouflage strategy, and a cosine-similarity-based update strategy. When applied to the shape optimization problem of Combined Quartic Generalized Spherical Interpolation (CQGBI) curves, this method enables the design of smoother curve shapes—based on parameters derived from the MISBOA approach—thereby boosting power generation efficiency. To address the limitations of existing TDOA-based localization algorithms, Liao et al. proposed an improved Secretary bird Optimization Algorithm for target localization [28]. Simulation results demonstrate that, compared with traditional mathematical algorithms and other meta-heuristic algorithms, this algorithm exhibits superior robustness and localization accuracy across various simulation scenarios.

Although numerous metaheuristic algorithms have been proposed in recent decades, not all algorithms exhibit sufficient adaptability and scalability for complex optimization tasks such as multilevel image segmentation. Compared with many conventional metaphor-based optimizers that rely on relatively simple update mechanisms, the Secretary Bird Optimization Algorithm (SBOA) possesses a distinctive multi-stage search framework inspired by prey searching, exhausting, attacking, and escape behaviors. These mechanisms naturally correspond to different optimization phases, including global exploration, transition search, local exploitation, and diversity maintenance. Such characteristics provide SBOA with stronger behavioral interpretability and greater flexibility for balancing exploration and exploitation.

In addition, SBOA is a recently developed optimization algorithm with considerable research potential. Although it has demonstrated promising performance in several engineering optimization tasks, its search mechanism still suffers from several limitations, including insufficient population diversity, weak local exploitation ability, and premature convergence when solving high-dimensional multimodal problems. These shortcomings become particularly significant in multilevel threshold image segmentation problems, where the search space is highly nonlinear, strongly coupled, and contains numerous local optima.

Therefore, SBOA provides a meaningful and extensible optimization framework for further investigation. Motivated by its unique staged-search mechanism and existing limitations, this study develops an enhanced SBOA to improve population diversity, strengthen exploration–exploitation balance, and enhance convergence robustness for complex numerical optimization and artistic image segmentation tasks.

However, according to the No Free Lunch Theorem, no single optimization algorithm can consistently maintain optimal performance across all optimization problems. In other words, the fact that an algorithm performs exceptionally well on certain problems does not guarantee that it will possess a similar advantage on others. Different optimization problems often feature distinct search space structures, variable dimensions, constraints, and objective function characteristics; consequently, they necessitate the adoption of search mechanisms and update strategies that are specifically tailored to the problem’s unique properties. Complex numerical optimization and artistic image segmentation problems, in particular, are frequently characterized by high dimensionality, multi-modality, non-linearity, and a susceptibility to becoming trapped in local optima; under such conditions, relying solely on the singular search mechanism of a standard algorithm makes it difficult to simultaneously balance global exploration capabilities with local exploitation capabilities. Therefore, building upon the SBOA framework, this paper introduces a suite of enhancement strategies designed to boost population diversity and strengthen the algorithm’s ability to escape local optima. By further improving its convergence speed, optimization accuracy, and robustness, these enhancements render the algorithm more effectively suited for addressing image segmentation problems. The specific contributions of this paper are as follows:

1. A chaotic elite initialization strategy is proposed to improve the quality of the initial population. By combining Tent chaotic mapping with elite-guided population reconstruction, the proposed strategy enhances the uniformity and diversity of the population distribution, thereby improving the algorithm’s ability to locate promising regions in the early search stage.
2. An adaptive spiral Lévy flight strategy is introduced to strengthen the balance between global exploration and local exploitation. By integrating spiral motion with adaptive Lévy flight, the proposed strategy enables the algorithm to maintain wide-range exploration in the early stage and fine local search in the later stage, thereby improving its ability to escape local optima.
3. A dynamic neighborhood-guided mutation strategy is developed to alleviate the problem of premature convergence. By dynamically adjusting the neighborhood radius and combining neighborhood learning with global-best guidance, the proposed strategy effectively maintains population diversity and enhances convergence accuracy in the later search stage.
4. Extensive numerical optimization experiments are conducted on the IEEE CEC2014, CEC2017, and CEC2020 benchmark suites to evaluate the performance of MISBOA. The experimental results demonstrate that MISBOA achieves superior convergence speed, optimization accuracy, and robustness compared with several state-of-the-art metaheuristic algorithms.
5. MISBOA is successfully applied to Otsu-based multilevel threshold image segmentation. Experimental results on multiple benchmark images, evaluated by PSNR, FSIM, SSIM, and visual quality, verify that the proposed algorithm can generate more accurate and stable segmentation results for complex artistic images.

The remainder of this paper is organized as follows. Section 2 introduces the original Secretary Bird Optimization Algorithm and describes the improvement strategies incorporated into the proposed MISBOA in detail. Section 3 presents a comprehensive experimental evaluation of MISBOA on a series of benchmark functions for numerical optimization, demonstrating its effectiveness and robustness. Section 4 applies the proposed MIGSEA to artistic image segmentation tasks and assesses its performance through Peak Signal-to-Noise Ratio, Structural Similarity Index Measure, Feature Similarity Index, and visual quality comparisons. Finally, Section 5 summarizes the main conclusions of this study and discusses potential directions for future research.

## 2. Multi-Strategy Improved SBOA

The Secretary Bird Optimization Algorithm is a recently proposed swarm intelligence optimization method inspired by the hunting behavior of secretary birds in nature. By simulating different stages of prey searching, chasing, attacking, and escaping, SBOA is able to balance global exploration and local exploitation during the optimization process. Owing to its simple structure, few control parameters, and strong search capability, SBOA has shown promising performance in solving a variety of continuous optimization problems. However, when dealing with complex high-dimensional problems, SBOA may still suffer from insufficient population diversity, weak local exploitation ability, and premature convergence, which can limit its optimization accuracy and robustness.

To address these shortcomings, this section first provides a brief introduction to the original SBOA framework and its mathematical model. Subsequently, three effective improvement strategies are proposed to enhance the exploration capability, exploitation performance, and convergence accuracy of the algorithm. By integrating these strategies into the original SBOA, the proposed MISBOA is expected to achieve better optimization performance and stronger adaptability when solving numerical optimization and artistic image segmentation problems.

### 2.1. Secretary Bird Optimization Algorithm (SBOA)

The Secretary Bird Optimization Algorithm is a recently proposed swarm intelligence optimization algorithm inspired by the hunting and escape behaviors of secretary birds in nature. Secretary birds are well known for their ability to hunt snakes in grasslands. During hunting, they first search for prey over a wide range, then continuously circle around the prey to weaken its resistance, and finally launch a rapid and accurate attack. In addition, when facing predators, secretary birds can either camouflage themselves using the surrounding environment or quickly escape by running or flying. Based on these biological behaviors, SBOA divides the optimization process into two stages: hunting strategy and escape strategy, corresponding to the exploration and exploitation phases of the algorithm, respectively.

Assume that the population size is N, and the dimensionality of the optimization problem is dim. In SBOA, each secretary bird represents a candidate solution, and the population can be represented as:

(1) $$X=\left[\begin{array}{cccc} x_{1,1} & x_{1,2} & \ldots & x_{1,Dim}\\ x_{2,1} & x_{2,2} & \ldots & x_{2,Dim}\\ \vdots & \vdots & \ddots & \vdots \\ x_{N,1} & x_{N,2} & \ldots & x_{N,Dim} \end{array}\right]$$

where $x_{i,j}$ represents the value of the j-th dimension of the i-th secretary bird. The position of each individual is randomly initialized within the feasible search space as follows:

(2) $$x_{i,j}=lb_{j}+r\times (ub_{j}-lb_{j})$$

where $lb_{j}$ and $ub_{j}$ denote the lower and upper bounds of the j-th dimension, respectively, and r∈[0,1] is a random number. After initialization, the fitness values of all individuals are calculated according to the objective function:

(3) $$F=\left[\begin{array}{c} F(X_{1})\\ F(X_{2})\\ \vdots \\ F(X_{N}) \end{array}\right]$$

where $F(X_{i})$ denotes the fitness value of the i-th individual. In minimization problems, the individual with the smallest fitness value is considered the current best solution.

The search process of SBOA mainly consists of two phases. The first phase is the hunting stage, which is responsible for enhancing the exploration capability of the algorithm. The second phase is the escape stage, which focuses on improving the local exploitation ability and accelerating convergence. By combining these two stages, SBOA attempts to achieve a balance between global exploration and local exploitation.

#### 2.1.1. Hunting Strategy of Secretary Bird

The hunting behavior of secretary birds is divided into three stages: searching for prey, exhausting prey, and attacking prey. These three stages correspond to different search mechanisms in SBOA.

In the first stage, secretary birds search for prey over a wide area. This stage mainly occurs in the early iterations of the algorithm, where population diversity and global exploration are important. To simulate this process, SBOA updates the position of each individual according to the difference between two randomly selected individuals:

(4) $$x_{i,j}^{new}=x_{i,j}+(x_{r1,j}-x_{r2,j})\times R_{1}$$

where $x_{r1,j}$ and $x_{r2,j}$ are two randomly selected candidate solutions, and $R_{1}$ is a random vector in [0,1]. By using the positional difference between individuals, the algorithm can effectively expand the search range and avoid premature convergence. This mechanism improves the diversity of the population and enhances the global search ability of SBOA.

In the second stage, once the prey is found, secretary birds continuously move around it and provoke it in order to consume its energy. To model this behavior, SBOA combines Brownian motion with the current best solution:

(5) $$x_{i,j}^{new}=x_{best,j}+e^{(t/T{)}^{4}}\times (RB-0.5)\times (x_{best,j}-x_{i,j})$$

where $x_{best,j}$ is the best position found so far, RB denotes Brownian motion, t is the current iteration number, and T is the maximum number of iterations. Brownian motion introduces random local perturbations, enabling the algorithm to search around the best solution more effectively. At the same time, the exponential term dynamically adjusts the search range during the optimization process. This stage strengthens the exploitation ability of the algorithm near promising regions and improves convergence speed.

In the third stage, when the prey becomes weak, secretary birds launch a rapid and accurate attack. SBOA models this process by introducing Lévy flight:

(6) $$x_{i,j}^{new}=x_{best,j}+{\left(1-\frac{t}{T}\right)}^{2\frac{t}{T}}x_{i,j}\times RL$$

where RL denotes the weighted Lévy flight step size. Lévy flight is characterized by frequent short-distance movements and occasional long-distance jumps. Therefore, it allows the algorithm to perform local fine search while still maintaining the ability to jump out of local optima. In addition, the nonlinear weight factor dynamically adjusts the influence of Lévy flight according to the iteration process, helping the algorithm achieve a better balance between exploration and exploitation.

#### 2.1.2. Escape Strategy of Secretary Bird

When secretary birds encounter predators, they typically adopt two different escape behaviors. One is to camouflage themselves in the surrounding environment, while the other is to rapidly escape by running or flying. In SBOA, these two behaviors are modeled as:

(7) $$x_{i,j}^{new}=\left\{\begin{array}{c} x_{best,j}+(2\times RB-1)\times {\left(1-\frac{t}{T}\right)}^{2}\times x_{i,j},\;r_{i}<0.5\\ x_{i,j}+R_{2}\times (x_{random,j}-K\times x_{i,j}),\;\mathrm{otherwise} \end{array}\right.$$

where $R_{2}$ is a random vector, $x_{random,j}$ is a randomly selected solution, and K is a random integer equal to 1 or 2. In the first case, the individual moves toward the vicinity of the current best solution, which helps the algorithm exploit promising regions. In the second case, the individual updates its position according to a randomly selected solution, which enhances the diversity of the population and helps the algorithm avoid local optima. By alternately applying these two strategies, SBOA is able to maintain a dynamic balance between local search and global exploration, thereby improving its convergence accuracy and robustness. The pseudocode of the SBOA is outlined in Algorithm 1.
**Algorithm 1:** the pseudo-code of the SBOA1: Initialize the population $X_{i}\;(i\;=\;1,\;2,\;\ldots \;,\;N)$

2: Evaluate the fitness of all individuals 3: Determine the current best solution
$X_{best}$ 4: For t = 1 : T  do 5:
 For i = 1 : N do 6:  if t < 1/3 T then $7:\;\;\;\mathrm{Update}\;X_{i}$ using the prey-searching strategy 8:  else if 1/3 T ≤ t < 2/3 T then $9:\;\;\;\mathrm{Update}\;X_{i}$ using the prey-exhausting strategy 10:  else $11:\;\;\;\mathrm{Update}\;X_{i}$ using the prey-attacking strategy 12:  end if 13:  if rand < 0.5 then $14:\;\;\;\mathrm{Update}\;X_{i}$ using the camouflage strategy 15:  else $16:\;\;\;\mathrm{Update}\;X_{i}$ using the escape strategy 17:  end if 18:  Evaluate the new fitness of $X_{i}$ 19: End For 20: Update the current best solution $X_{best}$ 21: End For 22: Return $X_{best}$


### 2.2. Proposed MISBOA

It should be noted that the three proposed enhancement strategies are not directly derived from additional biological behaviors of secretary birds. Instead, they are algorithm-oriented improvement mechanisms designed according to the optimization characteristics and structural limitations of the original SBOA framework. The motivation of these strategies mainly comes from improving population diversity, strengthening the balance between global exploration and local exploitation, and alleviating premature convergence in complex optimization problems. Therefore, the proposed strategies should be regarded as computational enhancement mechanisms integrated into the SBOA framework, rather than newly introduced biological metaphors. Nevertheless, these strategies remain compatible with the staged-search structure of SBOA and further strengthen its optimization capability in different search phases.

The three proposed strategies were not introduced as arbitrary combinations of existing techniques, but were specifically designed to address different structural limitations of the original SBOA. In the original SBOA, the prey-searching stage mainly relies on randomly selected individuals, which may lead to insufficient population diversity and uneven search-space coverage during the early optimization stage. Therefore, the chaotic elite initialization strategy was introduced to improve the uniformity and quality of the initial population distribution.

In addition, although the original SBOA employs Lévy flight during the prey-attacking stage, its search trajectory remains relatively simple and lacks adaptive local refinement capability. To strengthen the transition from global exploration to local exploitation, the adaptive spiral Lévy flight strategy was incorporated to generate more flexible search trajectories and enhance exploitation around promising regions.

Furthermore, the original escape mechanism of SBOA mainly depends on random perturbation and best-solution guidance, which may gradually reduce population diversity in the later search stage and increase the risk of premature convergence. To alleviate this issue, the dynamic neighborhood-guided mutation strategy was developed to maintain cooperative information exchange among neighboring individuals while gradually enhancing convergence toward elite solutions.

Therefore, the three strategies are functionally complementary and correspond to different optimization stages of SBOA, rather than being simple combinations of generic metaheuristic operators.

#### 2.2.1. Chaotic Elite Initialization Strategy

In the original SBOA, the population is randomly initialized, which may lead to an uneven distribution of individuals and insufficient diversity in the early stage. This problem becomes more severe in multilevel threshold image segmentation because the threshold search space is very large and contains many local optima. To improve the quality of the initial population, a chaotic elite initialization strategy is introduced.

First, the Tent chaotic map is used to generate a uniformly distributed chaotic sequence:

(8) $$z_{k+1}=\left\{\begin{array}{c} \frac{z_{k}}{\mu },\;z_{k}<\mu \\ \frac{1-z_{k}}{1-\mu },\;z_{k}\geq \mu \end{array}\right.$$

where $z_{k}\in [0,1]$ is the chaotic variable, and μ is the control parameter. In this paper, μ=0.6, since this value provides better randomness and ergodicity. Then, the chaotic sequence is mapped into the search space:

(9) $$x_{i,j}=lb_{j}+z_{i,j}\times (ub_{j}-lb_{j})$$

After initialization, the fitness values of all individuals are calculated, and the top 20% of individuals are selected as elite individuals. The remaining individuals are regenerated around these elite solutions:

(10) $$x_{i,j}^{new}=x_{elite,j}+\lambda \times (x_{r1,j}-x_{r2,j})$$

where $x_{elite,j}$ is the position of an elite individual, $x_{r1,j}$ and $x_{r2,j}$ are two randomly selected individuals, and λ is the perturbation coefficient. In this study, λ=0.3.

**Figure 1:**
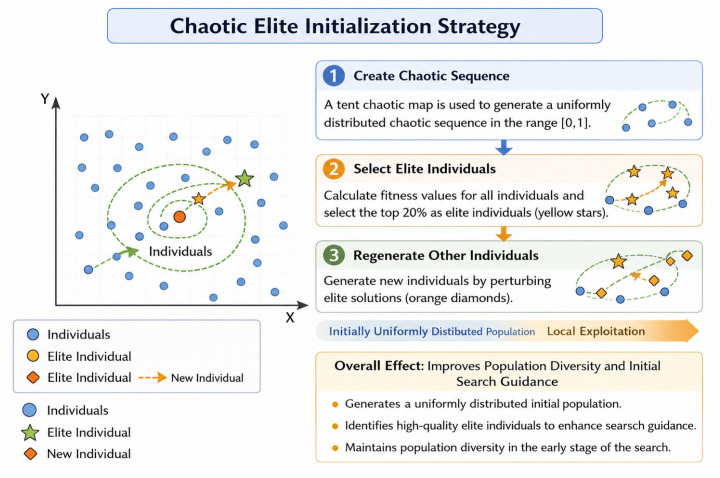
Schematic diagram of the Chaotic Elite Initialization Strategy.

Through this strategy, the population distribution becomes more uniform and diverse, which helps the algorithm locate promising regions in the early stage and improves the segmentation quality of complex artistic images. Figure 1 illustrates a schematic diagram of the Chaotic Elite Initialization Strategy.

#### 2.2.2. Adaptive Spiral Lévy Flight Strategy

In the original SBOA, Lévy flight is only used in the attacking stage, and its search pattern is relatively simple. However, for artistic image segmentation problems, especially multilevel threshold segmentation, the search process often requires both global jumps and fine local adjustment. Therefore, an adaptive spiral Lévy flight strategy is proposed to strengthen the balance between exploration and exploitation.

The position update rule is defined as:

(11) $$x_{i,j}^{new}=x_{best,j}+A\cdot e^{b\theta }\cdot cos(2\pi \theta )\cdot (x_{best,j}-x_{i,j})+L\cdot Levy(D)$$

where A is the adaptive weight factor, b is the spiral shape coefficient, θ∈[0,1] is a random number, and L is the adaptive Lévy flight coefficient. The adaptive weight factor is defined as:

(12) $$A=2\left(1-\frac{t}{T}\right)$$

The adaptive Lévy coefficient is defined as:

(13) $$L=0.5\left(1-\frac{t}{T}\right)+0.1$$

**Figure 2:**
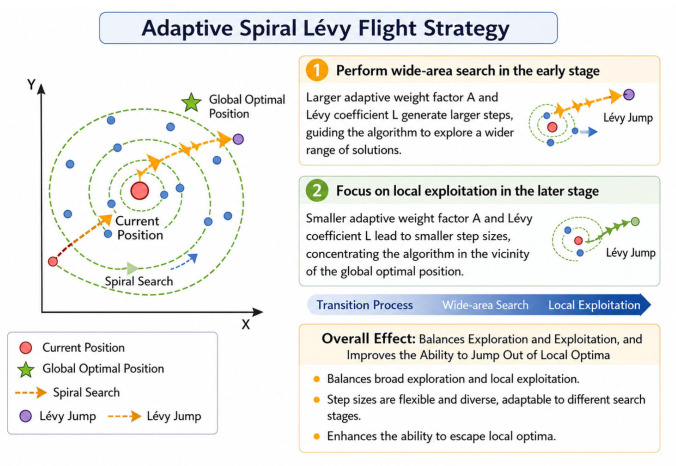
Schematic diagram of the Adaptive Spiral Lévy Flight Strategy.

In this paper, the spiral coefficient is set as b=1. In the early stage, larger values of A and L help the algorithm perform wide-range global exploration, while in the later stage, smaller values allow the algorithm to focus on local refinement. Compared with the original Lévy flight mechanism, the proposed strategy can generate more flexible search trajectories and improve the ability of the algorithm to jump out of local optima. Figure 2 illustrates a schematic diagram of the Adaptive Spiral Lévy Flight Strategy.

#### 2.2.3. Dynamic Neighborhood-Guided Mutation Strategy

When solving high-dimensional image segmentation problems, the original SBOA may easily lose population diversity in the later stage, causing premature convergence. To address this issue, a dynamic neighborhood-guided mutation strategy is proposed.

First, the neighborhood radius is defined as:

(14) $$R(t)=R_{max}\left(1-\frac{t}{T}\right)$$

where $R_{max}$ is the maximum neighborhood radius. In this paper, $R_{max}=0.5\times (ub-lb)$. For each individual, neighboring individuals within radius R(t) are selected, and the local neighborhood center is calculated as:

(15) $$X_{center}=\frac{1}{M}\sum_{k=1}^{M}X_{k}$$

where M is the number of neighboring individuals. Then, the mutation strategy is performed as:

(16) $$X_{i}^{new}=X_{i}+\alpha (X_{center}-X_{i})+\beta (X_{best}-X_{i})$$

where α and β are two adaptive coefficients:

(17) $$\alpha =0.5\left(1-\frac{t}{T}\right),\beta =0.5\frac{t}{T}$$

At the beginning of the search, a larger α helps individuals learn more from their neighbors and maintain population diversity. In the later stage, a larger β makes individuals move closer to the global best solution, thereby improving convergence accuracy.

**Figure 3:**
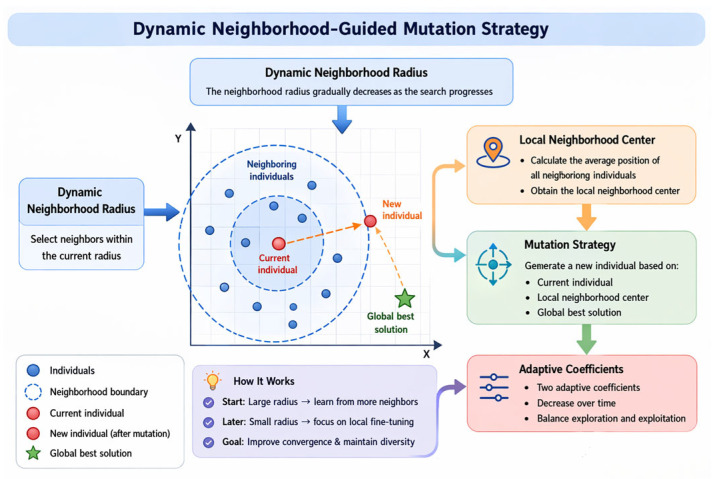
Schematic diagram of the Dynamic Neighborhood-Guided Mutation Strategy.

**Figure 4:**
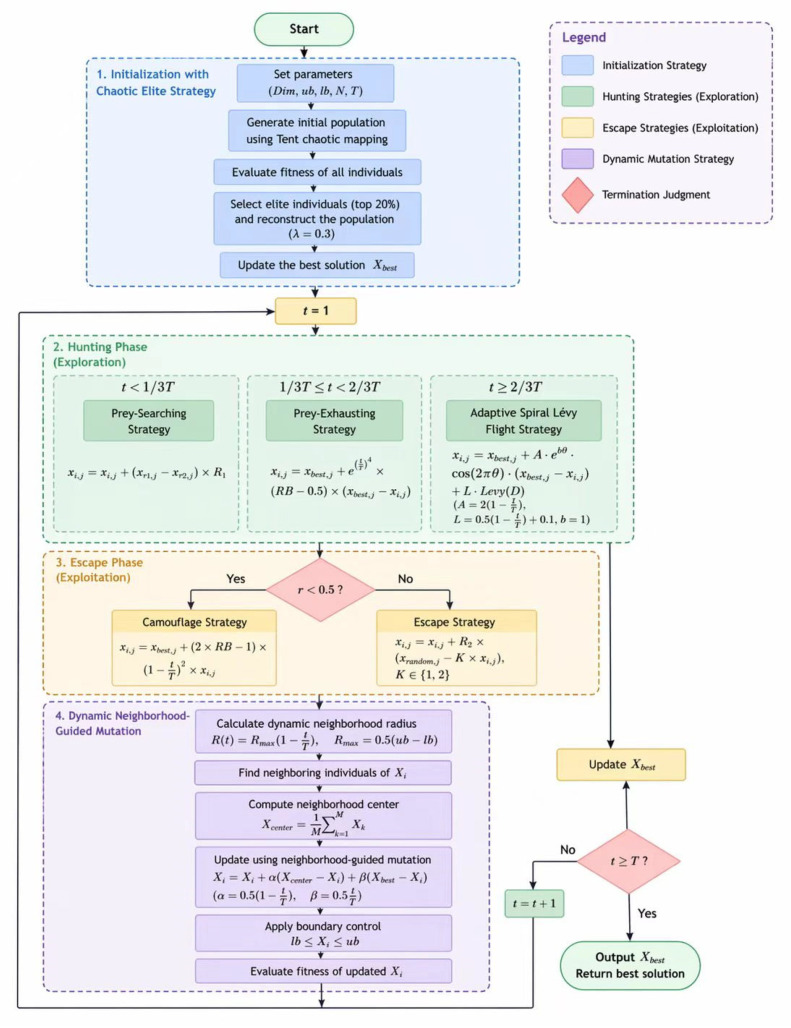
MISBOA flowchart.

This strategy allows the algorithm to dynamically adjust the influence of neighboring individuals and elite individuals during the search process, which is especially beneficial for artistic image segmentation tasks with many thresholds and complex texture distributions. Figure 3 illustrates a schematic diagram of the Dynamic Neighborhood-Guided Mutation Strategy. Algorithm 2 shows the pseudocode for MISBOA. Figure 4 shows the execution flowchart of MISBOA.
**Algorithm 2:** the pseudo-code of the MISBOA1:  Initialize Problem Setting (Dim, ub, lb, Pop_size (*N*), Max_Iter (*T*), Current_Iter (*t*)) 2:  Initialize the population using Tent chaotic mapping 3:  Evaluate the fitness of all Secretary Birds 4:  Select elite individuals and reconstruct the population 5:  Update Secretary Bird $X_{best}$ 6:  For t = 1 : T 7:  For i = 1 : N 8:   Exploration: 9:   if t < 1/3 T 10:    Calculate new status of the i-th Secretary Bird using prey-searching strategy 11:    Update the i-th Secretary Bird 12:   else if 1/3 T < t < 2/3 T 13:    Calculate new status of the i-th Secretary Bird using prey-exhausting strategy 14:    Update the i-th Secretary Bird 15:   else 16:    Calculate new status of the i-th Secretary Bird using adaptive spiral Lévy flight strategy 17:    Update the i-th Secretary Bird 18:   end if 19:   Exploitation: 20:   if r < 0.5 21:    Calculate new status of the i-th Secretary Bird using camouflage strategy 22:   else 23:    Calculate new status of the i-th Secretary Bird using escape strategy 24:   end if 25:   Dynamic neighborhood-guided mutation: 26:   Calculate the dynamic neighborhood radius R(t) 27:   Find neighboring individuals of the i-th Secretary Bird 28:   Calculate the neighborhood center $X_{center}$ 29:   Update the i-th Secretary Bird using neighborhood-guided mutation 30:   Apply boundary control 31:   Evaluate the fitness of the i-th Secretary Bird 32:  **end for**
i = 1 : N 33:  Save best candidate solution so far 34:  **end for**
t = 1 : T 35:  Output: The best solution obtained by MISBOA for given optimization problem 36:  Return best solution

### 2.3. Time Complexity Analysis of MISBOA

The computational complexity of an optimization algorithm is an important indicator for evaluating its efficiency and scalability. As per the original SBOA, let N denote the population size, Dim represent the problem dimension, and T be the maximum number of iterations. The time complexity of SBOA mainly consists of population initialization, fitness evaluation, and position updating. Therefore, the overall time complexity of SBOA can be expressed as O(N×(T×Dim+1)), which is dominated by the iterative update and fitness calculation processes.

For the proposed MISBOA, three additional strategies are incorporated into the original SBOA framework, namely the chaotic elite initialization strategy, the adaptive spiral Lévy flight strategy, and the dynamic neighborhood-guided mutation strategy. As a result, its computational complexity should be analyzed by considering both the original SBOA operations and the extra cost introduced by these enhancement mechanisms.

First, in the chaotic elite initialization strategy, the Tent chaotic mapping is used to generate the initial population, and then the elite individuals are selected to guide the regeneration of the remaining individuals. The generation of chaotic sequences requires traversing all individuals and dimensions once, resulting in a complexity of O(N×Dim). The elite selection operation usually requires sorting or ranking the population according to fitness values, whose complexity is O(NlogN). Since initialization is performed only once before the main iteration process, the total additional complexity introduced by this strategy is O(N×Dim+NlogN).

Second, in the adaptive spiral Lévy flight strategy, the position of each individual is updated using spiral motion and Lévy flight during the iterative process. Since the update is carried out for each individual in each dimension at every iteration, the additional computational cost of this strategy is O(T×N×Dim). It should be noted that Lévy flight itself is essentially a vector generation and scaling operation, so it does not change the order of complexity, but only increases the constant computational overhead.

Third, in the dynamic neighborhood-guided mutation strategy, each individual needs to calculate its neighborhood information and update its position accordingly. If the neighborhood center is estimated by scanning neighboring individuals, the worst-case computational cost for one iteration is $O(N^{2}\times Dim)$, because each individual may need to compare itself with all other individuals. Thus, over the whole optimization process, the complexity introduced by this strategy is $O(T{\times N}^{2}\times Dim)$. This term becomes the dominant part of the overall time complexity of MISBOA. Therefore, the total time complexity of MISBOA can be expressed as O(N×Dim+NlogN)+O(T×N×Dim)+O(T×N×N×Dim), which can be further simplified as $O(T\times N^{2}\times Dim)$. because the term $O(T\times N^{2}\times Dim)$ dominates the remaining lower-order terms.

Compared with the original SBOA, whose complexity is O(N×(T×Dim+1)), the proposed MISBOA has a higher computational complexity. The main reason is that the added dynamic neighborhood-guided mutation strategy introduces pairwise neighborhood interactions among individuals, which increases the complexity from approximately linear dependence on N to quadratic dependence on N. In other words, SBOA mainly performs individual-level position updates, while MISBOA further considers neighborhood-level cooperative mutation, leading to a greater computational burden.

Nevertheless, although MISBOA requires more computation time than SBOA, this increase is acceptable for two reasons. On the one hand, in practical optimization problems such as multilevel artistic image segmentation, the population size is usually set to a moderate value, so the quadratic term does not become prohibitively expensive. On the other hand, the three proposed strategies significantly enhance population diversity, strengthen the balance between exploration and exploitation, and improve the ability of the algorithm to escape local optima. Therefore, the additional computational cost is exchanged for better optimization accuracy, stronger robustness, and higher segmentation quality, which is worthwhile in complex image segmentation tasks.

## 3. Analysis of Numerical Optimization Experiments

This section presents a comprehensive numerical evaluation of the proposed MISBOA on the IEEE CEC2014 [29], IEEE CEC2017 [30], and CEC2020 benchmark suites [31]. First, a brief introduction to the characteristics of the three benchmark sets is provided. Then, the experimental settings are described in detail to ensure the reproducibility of the results. Subsequently, MISBOA is compared with nine representative optimization algorithms on all benchmark functions.

To guarantee a fair comparison and reduce the influence of randomness, all algorithms were tested under the same conditions, with a population size of 50 and a maximum number of 1000 iterations. Each algorithm was independently executed 30 times, and the experimental performance was assessed in terms of the mean and standard deviation of the obtained results. All experiments in this section were implemented on the MATLAB 2023a platform.

### 3.1. Numerical Optimization Test Set

The IEEE CEC2014, CEC2017, and CEC2020 test sets, all released by the IEEE Congress on Evolutionary Computation, constitute some of the most widely used standard benchmark sets for validating the performance of heuristic optimization algorithms. These test sets comprise a large collection of optimization functions exhibiting diverse characteristics, enabling a comprehensive evaluation of an algorithm’s search capability, convergence speed, stability, and global optimization ability when solving complex optimization problems.

Specifically, the CEC2014 test suite primarily comprises various problem types, including unimodal functions, multimodal functions, hybrid functions, and composite functions. Unimodal functions are typically employed to assess an algorithm’s local exploitation capabilities, whereas multimodal functions serve to evaluate its ability to escape local optima; hybrid and composite functions further heighten problem complexity, thereby enabling the assessment of an algorithm’s comprehensive performance within complex search spaces.

In comparison, the CEC2017 test suite features a more complex functional structure, incorporating a greater number of high-dimensional optimization problems characterized by rotation, translation, and non-separability; consequently, it imposes more stringent demands on an algorithm’s global exploration capabilities and robustness. This test suite is generally regarded as more challenging than CEC2014, as it is capable of more comprehensively reflecting the performance disparities among different algorithms.

The CEC2020 test suite further increases the proportion of complex hybrid and composite functions, while placing greater emphasis on the capability to solve high-dimensional, complex problems characterized by multiple local optima. Given its more intricate function landscapes and stronger coupling relationships among variables, it is frequently employed to validate the adaptability and generalization capabilities of novel, improved algorithms within complex optimization environments.

Overall, the CEC2014, CEC2017, and CEC2020 test suites encompass a wide range of optimization scenarios—spanning from simple to complex, low-dimensional to high-dimensional, and unimodal to complex combinatorial problems—and are therefore widely utilized for the performance validation and comparative analysis of heuristic algorithms, meta-heuristic algorithms, and swarm intelligence optimization algorithms.

### 3.2. Competitor Algorithms and Parameters Setting

To validate the performance of MISBOA, numerical optimization experiments are conducted in this subsection to analyze and compare it with nine other novel heuristic algorithms. The selected algorithms include the Bounty Hunter Optimizer (BHO), Newton Downhill Optimizer (NDO), Gekko Japonicus Algorithm (GJA), Stochastic social learning optimization (SSLO), LangEvin Equation (LEE), Snow Ablation Optimizer (SAO), Red-billed Blue Magpie Optimizer (RBMO), Escape Algorithm (ESC), and the standard Secretary bird optimization algorithm (SBOA). In the comparative experiments, the parameter settings of all algorithms were determined according to their original references. For clarity, Table 1 lists the detailed parameter configurations of each algorithm together with the corresponding references for further consultation.

### 3.3. Analysis of Numerical Optimization Experimental Results

#### 3.3.1. Performance Comparison on the IEEE CEC2014 Benchmark Functions

**Figure 5:**
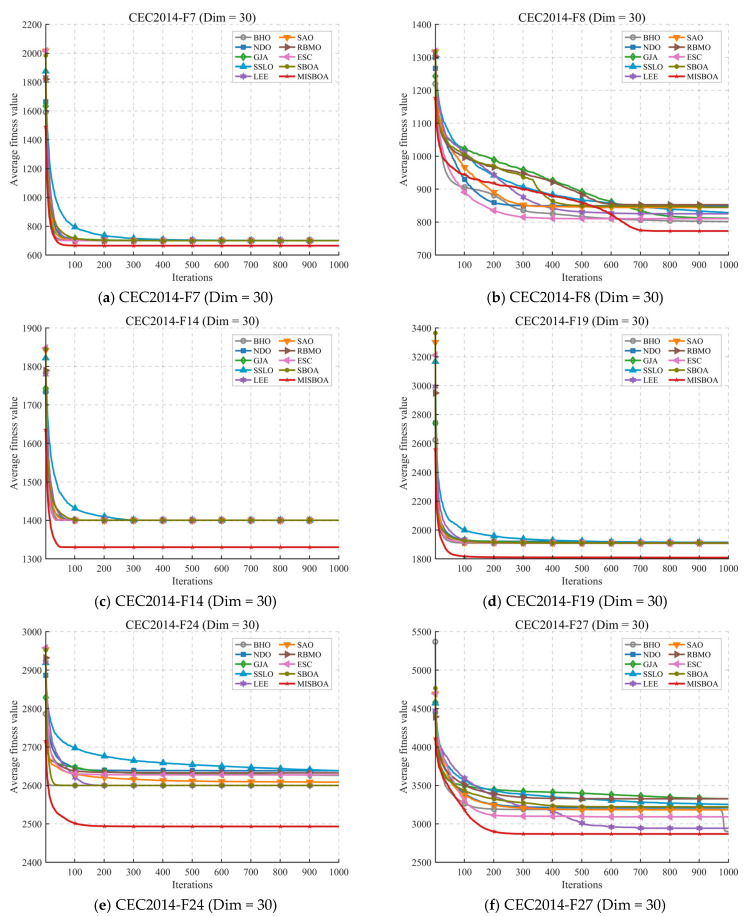
Convergence Speed Comparison of Different Algorithms on the CEC2014 Test Suite.

**Figure 6:**
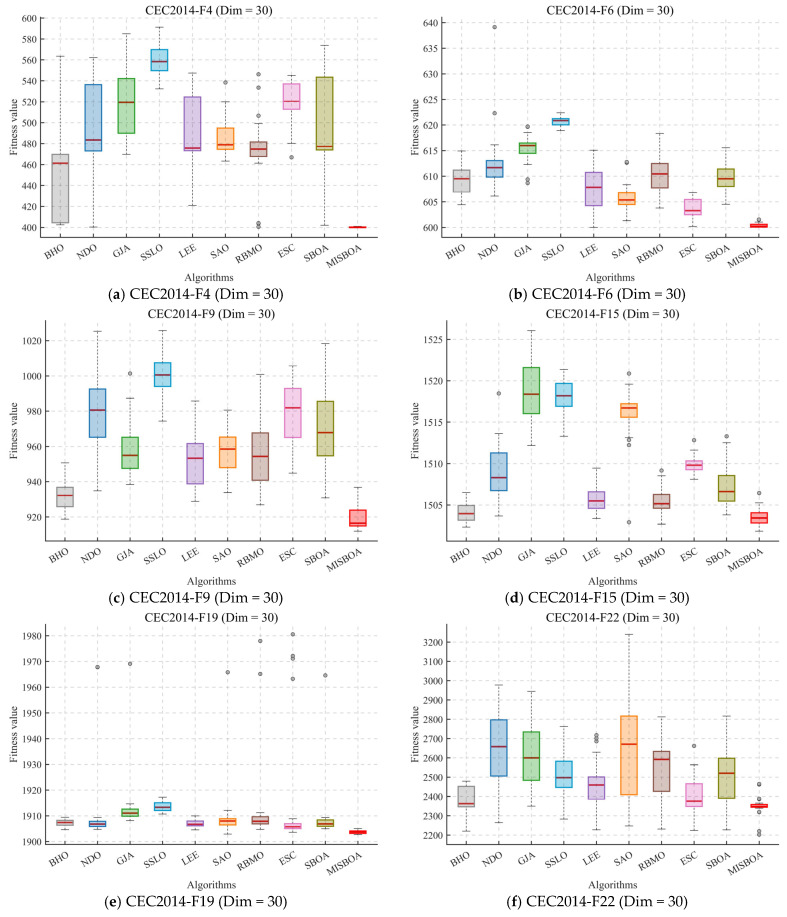
Boxplot-Based Performance Comparison of Different Algorithms on the CEC2014 Test Suite.

In this section, the numerical optimization performance of MISBOA is evaluated on the 30-dimensional functions of the CEC2014 benchmark suite. MISBOA is compared with nine representative state-of-the-art algorithms, and the corresponding optimization results are reported in Table 2. In the table, “Ave” and “Std” denote the mean and standard deviation of the results obtained from 30 independent runs, respectively. To further illustrate the search behavior of the compared algorithms, the convergence curves of the 10 algorithms are shown in Figure 5. In addition, Figure 6 presents the boxplot results of 30 independent runs for each algorithm, which are used to evaluate the robustness and stability of the algorithms while reducing the influence of random factors.

Figure 5 illustrates the convergence process of each algorithm on some functions of the CEC2014 test set. Overall, MISBOA exhibits a faster descent rate and better final convergence results on most test functions, indicating that it achieves a more effective balance between global search and local exploitation. Especially on functions such as F7, F14, and F20, MISBOA can rapidly reduce the objective function value in the early stages of iteration and maintain a relatively stable convergence trend in subsequent iterations, demonstrating strong search efficiency and stability. On relatively complex problems such as F8, F24, and F27, although some comparative algorithms can gradually approach the optimal region, MISBOA still demonstrates a faster convergence speed and stronger continuous optimization ability, obtaining higher-quality solutions with fewer iterations. In summary, Figure 5 fully illustrates that the proposed improvement strategy can effectively improve the algorithm’s optimization efficiency and enhance its ability to escape local optima, thus enabling MISBOA to exhibit superior convergence performance and competitive advantage on the CEC2014 benchmark functions.

Table 2 presents the mean and standard deviation results of each algorithm on the 30-dimensional CEC2014 benchmark functions. Overall, MISBOA achieves optimal or near-optimal results on most test functions, demonstrating strong optimization accuracy and stability. For unimodal functions such as F1–F4, MISBOA obtains significantly better average fitness values than the compared algorithms, indicating that the proposed improvement strategies effectively enhance local exploitation and convergence accuracy. For multimodal and hybrid functions such as F17, F18, F20, F21, and F22, MISBOA also shows strong global search ability, suggesting that it can better escape local optima and locate high-quality solutions. It is worth noting that on CEC2014-F23, MISBOA reaches the theoretical optimum value of 2500, with a standard deviation close to zero, while none of the other compared algorithms can achieve this result. This indicates that MISBOA not only finds the global optimum on this function but also maintains highly consistent performance across 30 independent runs. In addition, MISBOA exhibits relatively small standard deviations on many functions, further confirming its robustness and stability. Although MISBOA does not obtain the best results on a few functions, its overall performance remains highly competitive. Therefore, the results in Table 2 demonstrate that MISBOA has excellent convergence accuracy, global optimization capability, and robustness on the CEC2014 benchmark suite.

Figure 6 shows the box plot results of each algorithm on some functions of the CEC2014 test set, reflecting the distribution, stability, and resistance to random disturbances of different algorithms in 30 independent runs. Overall, MISBOA generally has a smaller box width, a better median position, and a shorter upper and lower bound, indicating that it maintains high consistency and stability across multiple independent runs. Particularly on functions F7, F14, F20, F24, and F30, MISBOA’s box plot distribution is more concentrated with fewer outliers, indicating that it not only obtains better solutions but also exhibits less fluctuation and strong robustness. In contrast, some of the compared algorithms have wider boxes and more outliers, indicating that these algorithms are susceptible to random initialization and local optima, leading to unstable results. Overall, Figure 6 further verifies the stability and reliability of MISBOA on the CEC2014 test set, demonstrating that the proposed improvement strategy can effectively improve the algorithm’s robustness and reduce the impact of random factors on the optimization results.

#### 3.3.2. Performance Comparison on the IEEE CEC2017 Benchmark Functions

**Figure 7:**
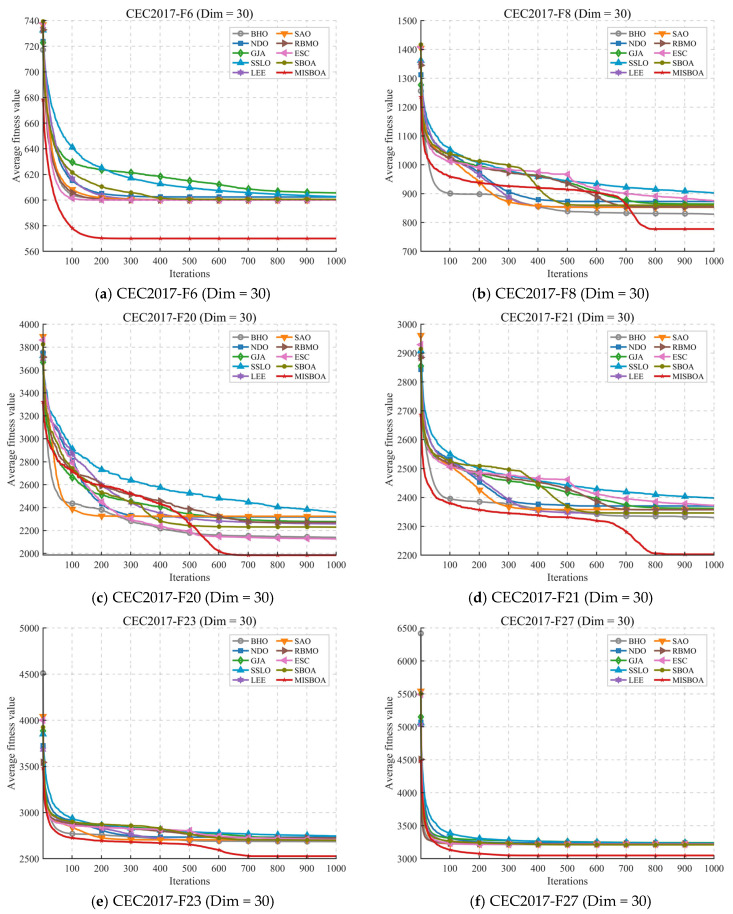

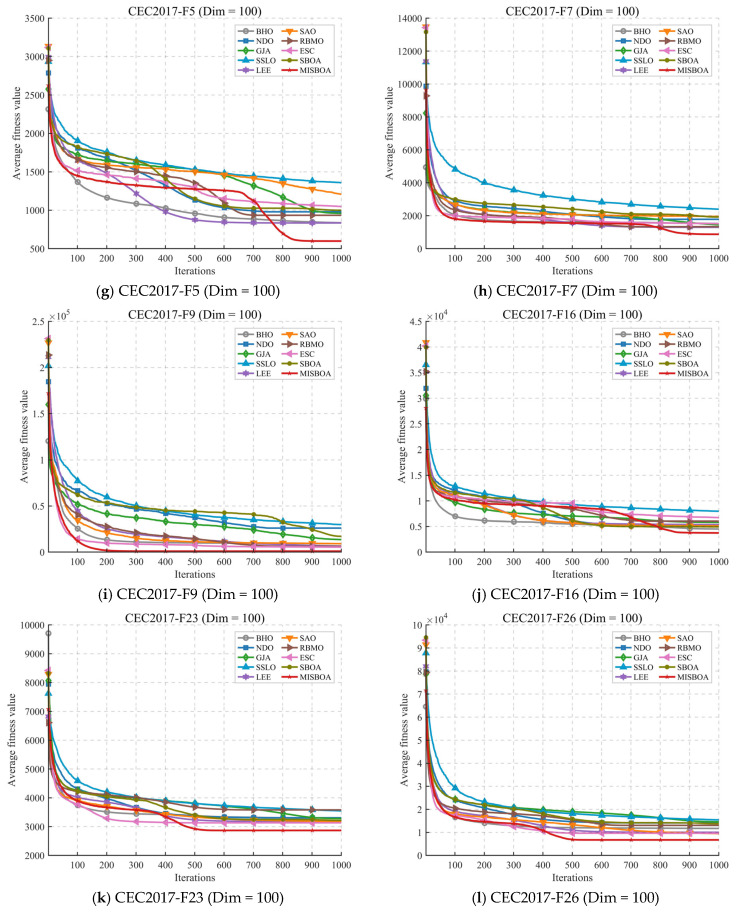
Convergence Speed Comparison of Different Algorithms on the CEC2017 Test Suite.

**Figure 8:**
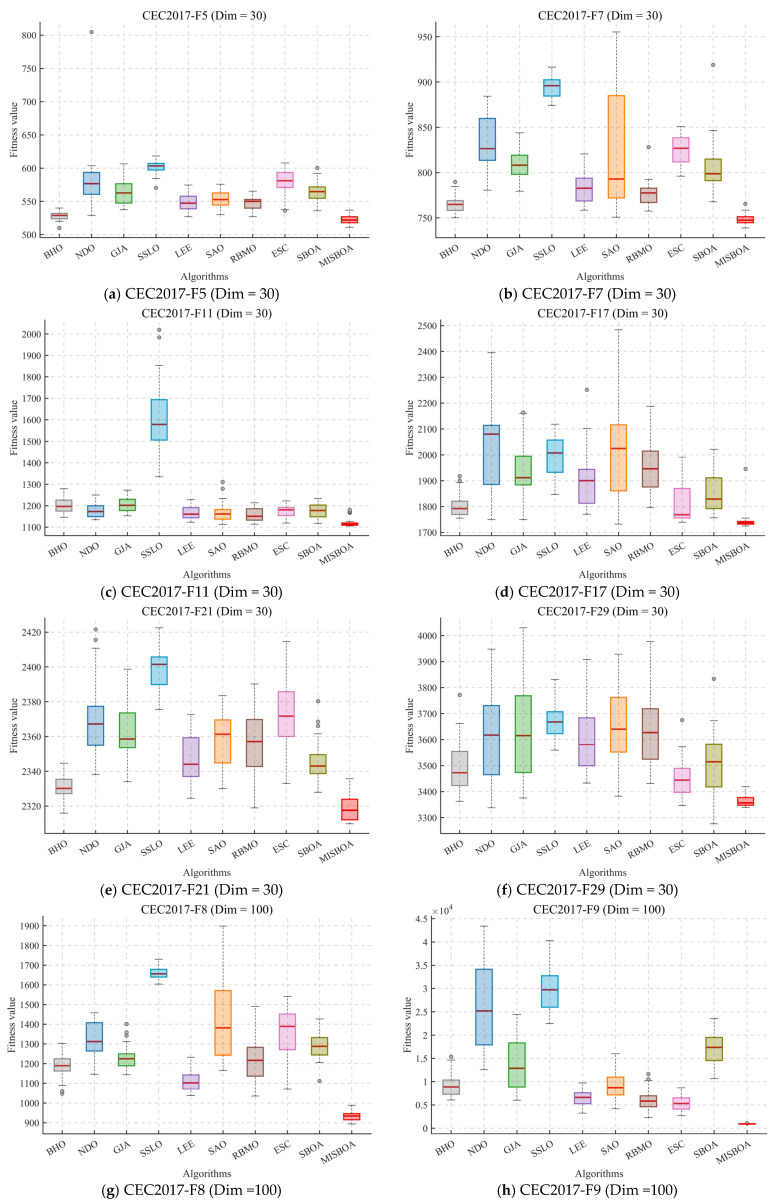

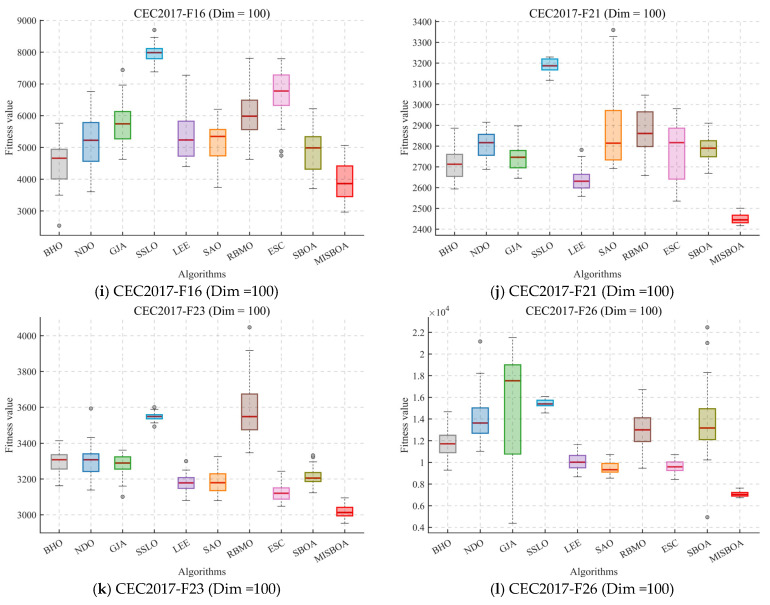
Boxplot-Based Performance Comparison of Different Algorithms on the CEC2017 Test Suite.

In this section, we evaluate the numerical optimization performance of MISBOA using the 30-dimensional and 100-dimensional functions from the CEC2017 benchmark suite. Specifically, MISBOA is compared against nine representative state-of-the-art algorithms, and the corresponding optimization results are presented in Table 3 and Table 4. In these tables, “Ave” and “Std” denote the mean and standard deviation, respectively, of the results obtained from 30 independent runs. To further elucidate the search behaviors of the compared algorithms, Figure 7 displays the convergence curves for these 10 algorithms. Furthermore, Figure 8 presents box plots of the results for each algorithm across the 30 independent runs; these results serve to assess the robustness and stability of the algorithms while simultaneously helping to mitigate the influence of stochastic factors.

Figure 7 shows the convergence curves of each algorithm on some functions of the CEC2017 test set. Overall, MISBOA exhibits faster convergence speed and better final convergence accuracy on most test functions, indicating that it can more effectively balance global search and local exploitation. In the early stages of iteration, MISBOA often rapidly reduces the objective function value and quickly enters the advantageous search region; in later iterations, its convergence curve continues to decline, unlike some of the comparative algorithms which plateau prematurely, demonstrating its strong ability to escape local optima and its continuous optimization capability. Especially on complex mixed and combined functions, MISBOA’s curve is usually below all other algorithms, and it can reach a better solution with fewer iterations, showing its higher search efficiency in handling high-dimensional, nonlinear, and multi-modal optimization problems. In contrast, while some of the comparative algorithms show a faster decline trend in the early stages, they are prone to getting trapped in local optima later, resulting in lower final convergence accuracy. Overall, Figure 7 fully illustrates that the chaotic elite initialization, adaptive spiral Lévy flight, and dynamic neighborhood-guided mutation strategies introduced by MISBOA can effectively enhance the algorithm’s exploration and development capabilities, thereby achieving better convergence performance on the CEC2017 test set.

Table 3 and Table 4 present the mean and standard deviation results of each algorithm on the 30-dimensional and 100-dimensional CEC2017 benchmark functions, respectively. Overall, MISBOA achieved optimal or near-optimal performance in both dimensional settings, demonstrating strong global search capability, local exploitation capability, and good dimensional adaptability. In the 30-dimensional test, MISBOA exhibited lower mean fitness values and smaller standard deviations on most unimodal, multimodal, mixed, and combined functions, indicating that it not only obtains higher-quality solutions but also achieves more stable results. As the problem dimension increased to 100 dimensions, the performance of most compared algorithms showed a significant decline, with increased mean values and greater volatility, indicating that they are more prone to getting trapped in local optima or premature convergence in high-dimensional complex problems. However, MISBOA still maintained superior solution results and a smaller standard deviation, continuing to outperform SBOA and other compared algorithms on most test functions, demonstrating strong robustness and scalability. This demonstrates that the proposed chaotic elite initialization strategy improves population diversity, the adaptive spiral Lévy flight strategy enhances global search capabilities, and the dynamic neighborhood-guided mutation strategy further improves the algorithm’s local exploitation capabilities in high-dimensional complex search spaces. Therefore, MISBOA’s superior performance on the 30-dimensional and 100-dimensional CEC2017 benchmark functions fully validates the effectiveness and generalization ability of the proposed improved strategy.

Figure 8 shows the box plot results of each algorithm on some functions of the CEC2017 test set, used to further evaluate the stability and robustness of different algorithms in 30 independent runs. As can be seen from the figure, MISBOA has a narrower overall box, a better median position, and a shorter upper and lower whisker range, indicating that it has smaller result fluctuations and higher consistency in multiple independent runs. Especially on some complex mixed and combined functions, the box plot distribution of MISBOA is more concentrated, with fewer outliers, indicating that it can maintain good stability in complex search environments and effectively reduce the impact of random factors on the optimization results. In contrast, although some of the compared algorithms can obtain good optimal values on a few functions, their boxes are wider, their upper and lower whiskers are longer, and they are accompanied by more outliers, indicating that the results of these algorithms fluctuate more and are easily affected by the initial population and local optima, resulting in relatively poor stability. Overall, Figure 8 further validates the robustness and reliability of MISBOA on the CEC2017 test set, demonstrating that the proposed improvement strategy not only enhances the algorithm’s optimization ability but also strengthens its stable performance in complex high-dimensional optimization problems.

#### 3.3.3. Performance Comparison on the IEEE CEC2020 Benchmark Functions

**Figure 9:**
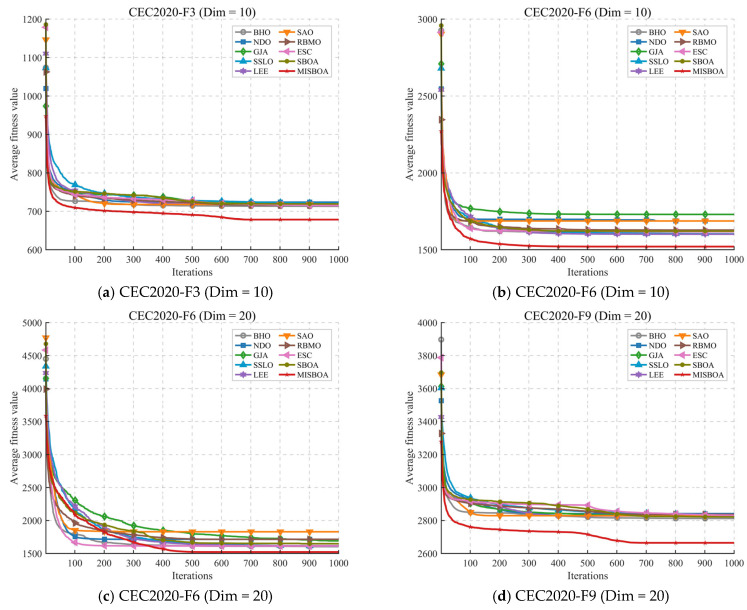
Convergence Speed Comparison of Different Algorithms on the CEC2020 Test Suite.

**Figure 10:**
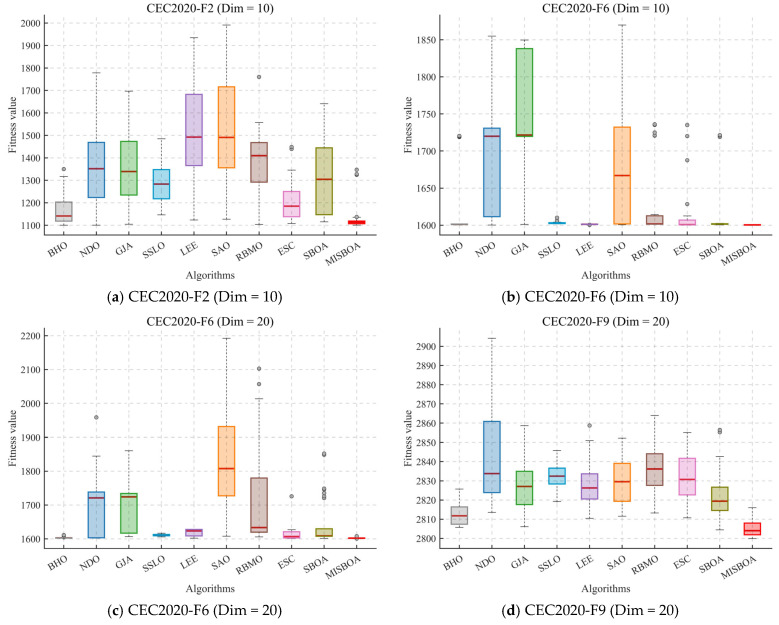
Boxplot-Based Performance Comparison of Different Algorithms on the CEC2020 Test Suite.

In this section, we evaluate the numerical optimization performance of MISBOA based on the 10-dimensional and 20-dimensional functions of the CEC2020 benchmark test suite. Specifically, MISBOA is compared against nine representative state-of-the-art algorithms, and the corresponding optimization results are presented in Table 5 and Table 6. In these tables, “Ave” and “Std” denote the mean and standard deviation, respectively, of the results obtained from 30 independent runs. To further elucidate the search behaviors of the compared algorithms, Figure 9 displays the convergence curves for these 10 algorithms. Furthermore, Figure 10 presents the box plots of the results for each algorithm across the 30 independent runs; these results serve to assess the robustness and stability of the algorithms while helping to mitigate the influence of stochastic factors.

Figure 9 shows the convergence curves of various algorithms on some functions of the CEC2020 test set. Overall, MISBOA exhibits faster convergence speed and better final optimization results on most functions. Especially on complex mixed and combined functions, MISBOA can rapidly reduce the objective function value in the early stages of iteration and maintain a downward trend in subsequent iterations, indicating its strong global search and local optimization capabilities. In contrast, some of the compared algorithms, while converging quickly in the early stages, tend to stagnate in the middle and later stages, making it difficult to further approach the global optimum; others show a slower rate of decrease throughout the optimization process, indicating a strong local optimum trap in complex high-dimensional search spaces. MISBOA’s convergence curve is generally lower than other algorithms and can obtain better solutions with fewer iterations, demonstrating its higher search efficiency and stronger continuous optimization capability. In summary, Figure 9 illustrates that the proposed chaotic elite initialization, adaptive spiral Lévy flight, and dynamic neighborhood-guided mutation strategies can effectively enhance the algorithm’s exploration and development capabilities on complex test functions of CEC2020, thereby enabling MISBOA to achieve better convergence performance and higher optimization accuracy.

Figure 10 shows the box plot results of each algorithm on some functions of the CEC2020 test set, used to further evaluate the stability and robustness of different algorithms in multiple independent runs. As can be seen from the figure, MISBOA has narrower box sizes, lower medians, and shorter upper and lower bounds on most test functions, indicating that it maintains high consistency and small result fluctuations in 30 independent runs. Especially on complex mixed and combined functions, MISBOA has fewer outliers and a more concentrated box distribution, indicating that it can effectively reduce the impact of random initialization and complex search spaces on the optimization results, thus achieving more stable performance. In contrast, while some of the compared algorithms achieve better results on a few functions, their box sizes are wider and have more outliers, indicating that these algorithms are easily affected by random factors and have relatively poor stability. Overall, Figure 10 further verifies the robustness and reliability of MISBOA on the CEC2020 test set, demonstrating that the proposed improvement strategies can not only improve the convergence accuracy of the algorithms but also enhance their stability in complex high-dimensional optimization problems.

Table 5 and Table 6 present the mean and standard deviation results of each algorithm on the 10-dimensional and 20-dimensional CEC2020 benchmark functions, respectively. Overall, MISBOA achieved optimal or near-optimal performance in both dimensional settings, demonstrating strong search capabilities and good dimensionality adaptability. In the 10-dimensional test, MISBOA exhibited lower mean fitness values and smaller standard deviations on most functions, indicating that it not only obtains better solutions but also possesses high stability and robustness. As the problem dimension increased to 20 dimensions, the performance of most compared algorithms decreased to varying degrees, manifested as increased mean values, wider fluctuation ranges, and significantly increased standard deviations. This suggests that these algorithms are more prone to getting trapped in local optima in more complex search spaces. However, MISBOA still maintained superior solution accuracy and a small standard deviation on most test functions, demonstrating strong global search capabilities and stability in high-dimensional complex problems. Compared to the standard SBOA, MISBOA demonstrates significant improvements on most functions, further proving that the chaotic elite initialization strategy, the adaptive spiral Lévy flight strategy, and the dynamic neighborhood-guided mutation strategy play a positive role in enhancing population diversity, balancing exploration and exploitation, and improving local search accuracy. Therefore, the experimental results of MISBOA on the 10-dimensional and 20-dimensional CEC2020 benchmark functions fully validate its excellent optimization performance and good generalization ability.

### 3.4. Statistical Analysis of Algorithm Performance

Statistical analysis plays a crucial role in evaluating the performance of heuristic and meta-heuristic algorithms. Given that such algorithms typically involve random initialization and stochastic search mechanisms, the results obtained from a single run may not accurately reflect their true optimization capabilities. Consequently, relying solely on the best or average results is often insufficient to provide a fair and reliable comparison. Statistical analysis effectively mitigates the influence of randomness and reveals whether the observed performance differences between algorithms are statistically significant and robust. Furthermore, statistical methods offer deeper insights into an algorithm’s robustness, consistency, and overall superiority across various benchmark problems. Therefore, incorporating statistical analysis is indispensable for conducting a comprehensive and compelling evaluation of heuristic algorithms. In this subsection, we subjected the proposed MISBOA to the Wilcoxon signed-rank test and the Friedman Mean Rank Test; the specific details are presented below.

#### 3.4.1. Wilcoxon Signed-Rank Test

In the performance evaluation of heuristic algorithms, relying solely on mean values, standard deviations, or convergence curves often proves insufficient to comprehensively reflect the true differences between algorithms; this is because heuristic algorithms exhibit significant stochasticity, leading to potentially substantial fluctuations across different independent runs [40]. Consequently, it is essential to incorporate statistical methods to further validate experimental results. As a non-parametric statistical test, the Wilcoxon signed-rank test does not require data to follow a normal distribution, making it particularly well-suited for comparing the performance of heuristic algorithms across multiple independent runs. By conducting a significance analysis on the performance differences between two algorithms across various test functions, one can effectively determine whether any observed performance improvement is statistically significant rather than merely attributable to random factors. Furthermore, the Wilcoxon signed-rank test provides an intuitive visualization of the extent of the improved algorithm’s superiority over its counterparts through W/T/L (Win/Tie/Loss) results; as such, this test has emerged as a crucial tool for assessing the reliability and superiority of heuristic optimization algorithms. In this section, we performed the Wilcoxon signed-rank test on the MISBOA at a significance level (α=0.05); the experimental results are shown in Table 7.

Table 7 presents the W/T/L results—based on the Wilcoxon signed-rank test—comparing MISBOA against other benchmark algorithms. As indicated by the results, MISBOA demonstrates a distinct statistical advantage across various test sets and under different dimensionality conditions. On the CEC2014 (dim = 30) test set, MISBOA achieved a higher number of wins compared to the majority of the algorithms; for instance, its W/T/L scores against GJA and RBMO were 28/2/0 and 28/1/1, respectively. On the CEC2017 (dim = 30) test set, MISBOA’s superiority was even more pronounced, achieving a score of 30/0/0 against both NDO and SSLO, thereby indicating that it significantly outperformed these benchmark algorithms across all test functions. Even on the higher-dimensional CEC2017 (dim = 100) test set, MISBOA maintained a high winning ratio, achieving W/T/L scores of 28/0/2 against both NDO and SSLO, and 28/1/1 against ESC. Regarding the CEC2020 test set, MISBOA similarly exhibited significant advantages under both dim = 10 and dim = 20 conditions, with multiple comparative results reaching 10/0/0. Overall, MISBOA consistently yielded results significantly superior to those of the benchmark algorithms across the majority of test functions, demonstrating that the proposed multi-strategy improvement mechanism effectively enhances the algorithm’s global search capability, local exploitation capability, and overall stability.

#### 3.4.2. Friedman Mean Rank Test

Since the performance of heuristic algorithms often varies significantly across different test functions, relying solely on the optimal values, mean values, or standard deviations obtained from a single function makes it difficult to comprehensively evaluate an algorithm’s overall performance [41]. Consequently, in scenarios involving multiple algorithms and multiple test problems, it is necessary to employ more comprehensive statistical analysis methods to facilitate a holistic comparison. The Friedman Mean Rank Test is a non-parametric statistical method well-suited for comparing multiple algorithms across multiple test functions; it evaluates the relative merits of algorithms from a global perspective by ranking their performance on each test function and subsequently calculating their average ranks. Compared to a mere comparison of numerical results, the Friedman Mean Rank Test effectively mitigates the impact of random fluctuations and isolated outliers, thereby providing a more objective reflection of an algorithm’s stability, robustness, and comprehensive optimization capabilities across diverse problems. As a result, this method has become a widely adopted and essential statistical tool in the performance analysis of heuristic algorithms. In this subsection, we subjected MISBOA to the Friedman Mean Rank Test; the experimental results are presented in the table below.

Table 8 presents the results of the Friedman Mean Rank Test for each algorithm across various test sets. As the results indicate, MISBOA achieved the lowest mean rank (M.R.) and the highest final rank (T.R.) across all test sets and under varying dimensional conditions, demonstrating its superior overall performance. In the CEC2014 (dim = 30) test set, MISBOA attained a mean rank of 2.00 and a final rank of 1st—significantly outperforming the other comparative algorithms—with BHO securing second place with a mean rank of 2.30. In the CEC2017 (dim = 30) and CEC2017 (dim = 100) test sets, MISBOA achieved mean ranks of 1.03 and 1.87, respectively, ranking 1st in both instances; this demonstrates that the algorithm maintains strong competitiveness in complex optimization problems of both medium and high dimensions. Notably, in the CEC2017 (dim = 30) test set, MISBOA’s mean rank approached the theoretical optimum of 1, indicating that it achieved the best results across the vast majority of the test functions. For the CEC2020 test set, MISBOA’s superiority was equally evident. Under the conditions of dim = 10 and dim = 20, its mean ranks were 1.30 and 1.20, respectively—firmly securing the first-place position in both cases—while the mean ranks of the other algorithms generally exceeded 2, with some even surpassing 7. This suggests that MISBOA not only possesses robust global search capabilities but also maintains stable optimization performance across test functions of varying dimensions and types. Overall, MISBOA achieved the best comprehensive ranking across all test sets, demonstrating that the proposed multi-strategy improvement mechanism effectively enhances the algorithm’s convergence performance, search precision, and stability, thereby enabling it to exhibit superior overall competitiveness across a wide range of complex optimization problems.

### 3.5. Comparison with CEC Competition Winners

Comparison with CEC competition winners is of great significance in the evaluation of metaheuristic optimization algorithms because the winning algorithms in CEC competitions usually represent the state-of-the-art optimization performance under standardized benchmark environments. These algorithms are typically developed through extensive research and rigorous experimental validation, exhibiting strong global search capability, convergence accuracy, and robustness on complex optimization problems. Therefore, comparing the proposed algorithm with CEC competition winners can provide a more convincing assessment of its optimization effectiveness and competitiveness. Moreover, such comparisons help demonstrate whether the proposed algorithm can maintain superior performance when facing highly competitive and challenging optimization scenarios. Motivated by this, this subsection conducts experimental comparisons between MISBOA and several representative CEC competition-winning algorithms to further verify the effectiveness, robustness, and competitiveness of the proposed method. The experimental results are shown below.

**Figure 11:**
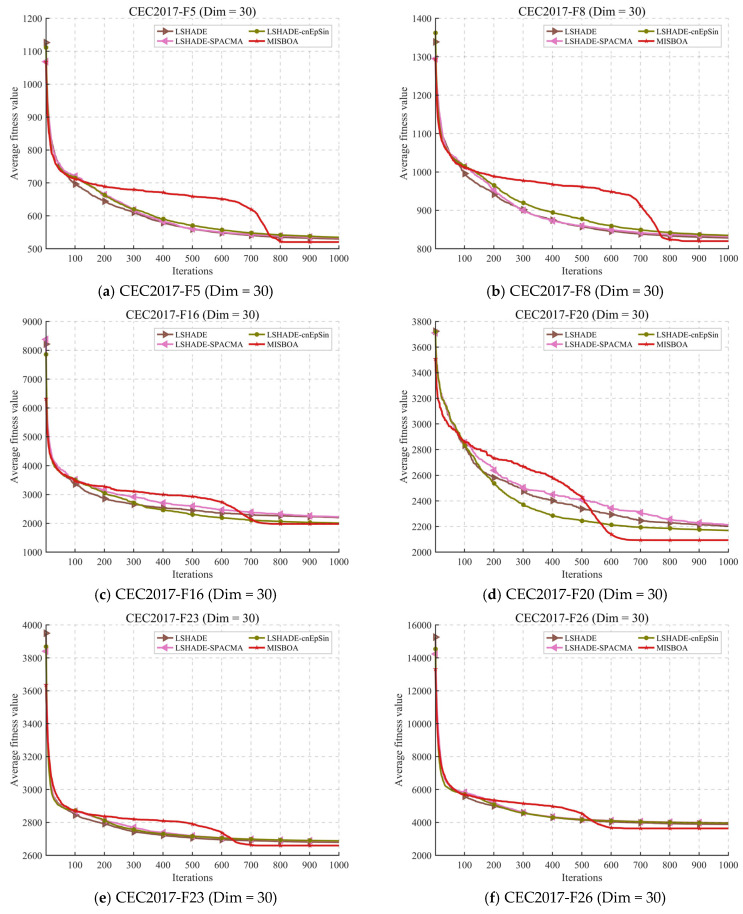
Convergence curves compared to the CEC winner algorithm.

Figure 11 presents the convergence curve comparisons between MISBOA and several representative CEC competition-winning algorithms, including LSHADE, LSHADE-cnEpSin, and LSHADE-SPACMA, on selected CEC2017 benchmark functions with 30 dimensions. Overall, MISBOA demonstrates highly competitive convergence behavior and achieves superior optimization performance on most test functions. Specifically, on functions such as F5, F8, and F16, although MISBOA exhibits a relatively slower convergence rate during the early and middle stages of the iteration process, it maintains stronger continuous optimization capability in the later stages and ultimately obtains lower fitness values than the compared winner algorithms. This phenomenon indicates that the proposed strategies effectively enhance the algorithm’s ability to escape local optima and maintain population diversity during the later search stage. On more complex functions such as F20, F23, and F26, MISBOA not only converges steadily throughout the optimization process but also achieves the best final convergence accuracy among all compared algorithms. In particular, the convergence curves of MISBOA exhibit more stable descending trends and lower final fitness values, demonstrating its strong balance between global exploration and local exploitation. Overall, the experimental results in Figure 11 verify that MISBOA possesses strong competitiveness even when compared with highly advanced CEC competition-winning algorithms, thereby further confirming the effectiveness, robustness, and optimization capability of the proposed enhancement strategies.

**Figure 12:**
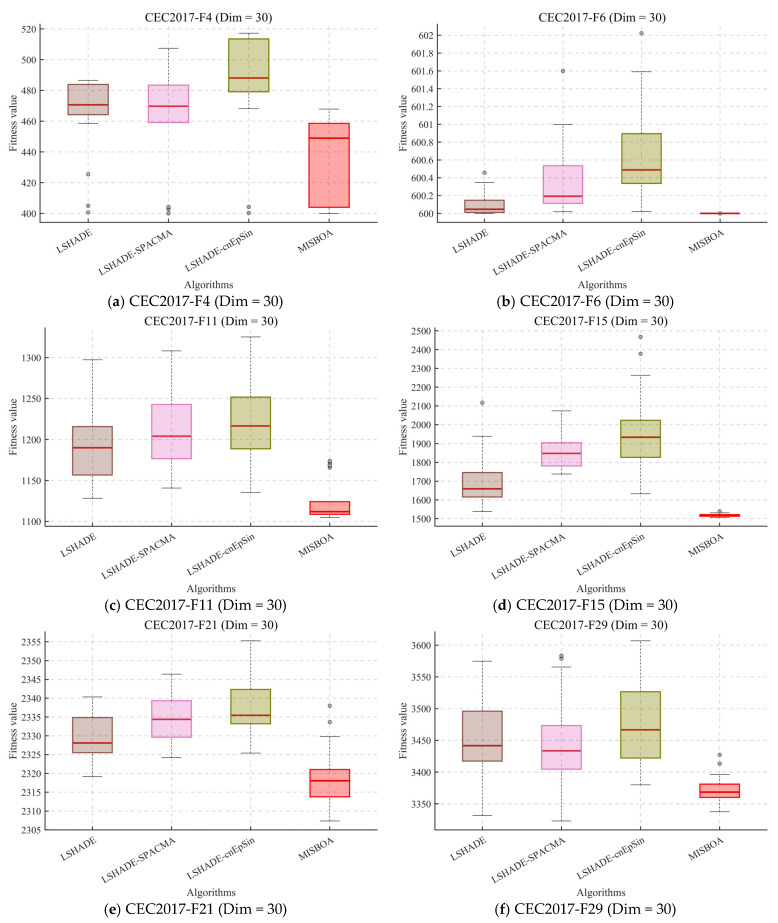
Box plots comparing the algorithm with the CEC winner.

Figure 12 presents the boxplot comparisons between MISBOA and several representative CEC competition-winning algorithms on selected CEC2017 benchmark functions with 30 dimensions. The boxplots are used to evaluate the distribution characteristics, robustness, and stability of the algorithms over 30 independent runs. Overall, MISBOA exhibits significantly superior distribution characteristics compared with the competing algorithms, demonstrating stronger robustness and more stable optimization performance. Specifically, on functions such as F11, F15, F21, and F29, MISBOA achieves lower median fitness values and narrower box ranges, indicating that the proposed algorithm can consistently obtain high-quality solutions with relatively small fluctuations across multiple independent runs. In addition, MISBOA generally produces fewer outliers and shorter upper and lower whiskers, which further demonstrates its strong resistance to random disturbances and its ability to maintain stable convergence behavior. Although on certain functions such as F4 the distribution range of MISBOA is relatively wider during some runs, its median fitness value still remains significantly better than those of the compared algorithms, indicating superior overall optimization capability. Particularly on F6, MISBOA shows an extremely concentrated distribution with almost negligible variance, demonstrating excellent convergence stability and reliability. Overall, the experimental results shown in Figure 12 further confirm that MISBOA not only achieves competitive convergence accuracy when compared with advanced CEC competition-winning algorithms, but also maintains strong robustness, stability, and consistency in repeated optimization experiments.

Table 9 presents the comparative experimental results between MISBOA and several representative CEC competition-winning algorithms on the 30-dimensional CEC2017 benchmark functions. The compared algorithms include LSHADE, LSHADE-SPACMA, and LSHADE-cnEpSin, which are widely recognized as highly competitive optimization algorithms in CEC competitions. Overall, MISBOA demonstrates highly competitive optimization performance and achieves superior or near-optimal results on most benchmark functions. Specifically, MISBOA obtains the best average fitness values on a large number of functions, including F1, F3, F4, F5, F6, F8, F9, F10, F11, F15, F16, F17, F20, F21, F22, F23, F24, F27, F28, F29, and F30, indicating its strong global optimization capability and convergence accuracy. Particularly on complex multimodal and hybrid functions such as F15, F20, F21, and F29, MISBOA exhibits significantly lower fitness values than the compared winner algorithms, demonstrating that the proposed strategies can effectively enhance exploration capability and improve the ability to escape local optima.

In addition, the standard deviation results further verify the robustness and stability of MISBOA. On many benchmark functions, MISBOA achieves relatively smaller standard deviation values, indicating that the proposed algorithm maintains stable optimization performance and strong resistance to random disturbances over 30 independent runs. Especially on functions such as F1, F6, F10, F15, and F29, MISBOA not only achieves the best average performance but also exhibits highly concentrated result distributions, demonstrating excellent convergence reliability and consistency. Although MISBOA does not achieve the absolute best performance on a few functions, its results remain highly competitive and generally close to those of the compared algorithms. Overall, the experimental results in Table 9 demonstrate that MISBOA possesses strong competitiveness even when compared with advanced CEC competition-winning algorithms, thereby further validating the effectiveness of the proposed enhancement strategies in improving convergence accuracy, robustness, and optimization capability on complex optimization problems.

### 3.6. Strategy Effectiveness Analysis

Strategy effectiveness analysis plays a crucial role in the evaluation of metaheuristic optimization algorithms because it can directly reveal the contribution of each improvement strategy to the overall optimization performance. Since many improved metaheuristic algorithms are constructed by integrating multiple enhancement mechanisms, merely reporting the final optimization results is often insufficient to fully demonstrate the effectiveness and rationality of the proposed strategies. Through strategy effectiveness analysis, it is possible to investigate whether each strategy can effectively improve specific optimization behaviors, such as population diversity maintenance, exploration capability, exploitation performance, convergence speed, and the ability to escape local optima. In addition, such analysis helps verify the complementarity and cooperative interaction among different strategies, thereby providing more convincing evidence for the effectiveness and necessity of the proposed improvements. Therefore, to comprehensively evaluate the contribution of each proposed strategy in MISBOA, this subsection conducts a detailed strategy effectiveness analysis experiment. Among them, SBOA1 indicates that the Chaotic Elite Initialization Strategy algorithm has been added to the SBOA, SBOA2 indicates that the Adaptive Spiral Lévy Flight Strategy algorithm has been added to the SBOA, and SBOA3 indicates that the Dynamic Neighborhood-Guided Mutation Strategy algorithm has been added to the SBOA.

Table 10 presents the strategy effectiveness analysis results of MISBOA on the CEC2014, CEC2017, and CEC2020 benchmark suites under different dimensional settings. The experimental results are evaluated using Mean Rank (M.R) and Total Rank (T.R), where lower values indicate better overall optimization performance. Overall, as more improvement strategies are incorporated into the original SBOA framework, the ranking performance of the algorithm consistently improves across all benchmark suites, demonstrating the effectiveness and complementarity of the proposed strategies.

Specifically, the original SBOA generally exhibits the worst ranking performance on most benchmark sets, indicating that the standard SBOA still suffers from limitations such as insufficient population diversity and weak balance between exploration and exploitation when solving complex optimization problems. After introducing the first improvement strategy, SBOA1 shows noticeable performance improvement on all test suites, suggesting that the proposed initialization enhancement mechanism can effectively improve the early-stage search capability and population quality. Subsequently, SBOA2 further improves the mean rank values, indicating that the adaptive spiral Lévy flight strategy effectively strengthens the transition between global exploration and local exploitation. Moreover, SBOA3 achieves additional ranking improvements compared with SBOA2, demonstrating that the dynamic neighborhood-guided mutation strategy can effectively alleviate premature convergence and enhance population diversity maintenance in the later search stage.

Finally, MISBOA consistently achieves the best Mean Rank and Total Rank values on all benchmark suites and dimensional settings, including CEC2014, CEC2017 (30D and 100D), and CEC2020 (10D and 20D). In particular, MISBOA obtains rank values close to 1 on almost all test sets, indicating that the complete integration of the three proposed strategies provides the strongest optimization capability and the most stable performance among all compared variants. Overall, the results in Table 10 clearly verify that each proposed strategy contributes positively to the optimization performance of MISBOA, while the cooperative interaction among the three strategies further enhances the convergence accuracy, robustness, and global optimization capability of the algorithm.

### 3.7. Parameter Sensitivity Analysis

Parameter sensitivity analysis plays a crucial role in the performance evaluation of metaheuristic optimization algorithms, as the optimization behavior of these algorithms is often heavily influenced by the choice of control parameters. Different parameter settings significantly affect the balance between global exploration and local exploitation, convergence speed, population diversity, and solution accuracy. Therefore, conducting parameter sensitivity analysis is essential for assessing the stability, robustness, and adaptability of the proposed algorithm under different parameter configurations. Furthermore, parameter sensitivity analysis helps reveal the impact of key parameters on the algorithm’s search dynamics and provides guidance for selecting appropriate parameter values in practical optimization problems. Moreover, it can further verify whether the proposed algorithm can maintain stable optimization performance under different parameter conditions, thereby enhancing the reliability and persuasiveness of the experimental results. Therefore, to comprehensively evaluate the robustness and parameter adaptability of MISBOA, this section will conduct detailed parameter sensitivity analysis experiments on several representative hyperparameters.

**Figure 13:**
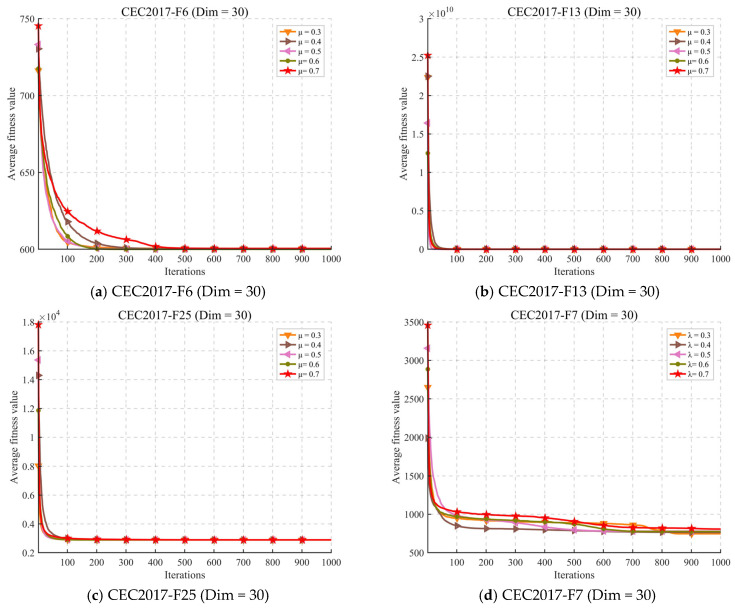

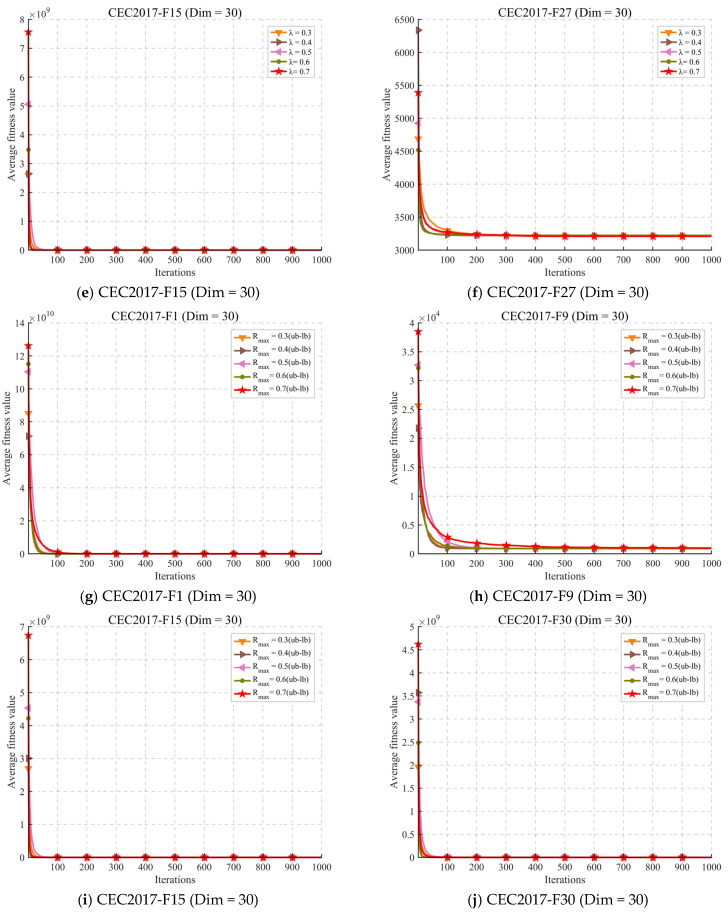
Parameter sensitivity analysis experimental results.

Figure 13 shows the parameter sensitivity analysis results of the MISBOA for several representative hyperparameters. Overall, MISBOA maintains relatively stable convergence behavior under different parameter configurations, indicating strong robustness and parameter adaptability. Specifically, different values of the control parameters have little impact on the convergence speed and final optimization accuracy of some benchmark functions; however, the overall convergence trend remains highly consistent. Among the tested parameter settings, moderate parameter values generally achieve better convergence performance and stability, reflecting a more balanced trade-off between global exploration and local exploitation. Furthermore, MISBOA maintains stable optimization capabilities under different parameter combinations, further validating the reliability and robustness of the proposed improvement strategy.

## 4. Multilevel Thresholding-Based Image Segmentation Model

In the context of multi-threshold image segmentation, the selection of thresholds directly impacts the effectiveness of distinguishing target regions from background regions. Common threshold selection methods primarily include Otsu’s method and Kapur’s entropy method. Specifically, Otsu’s method identifies the optimal threshold by maximizing the inter-class variance, whereas Kapur’s entropy method achieves image segmentation by maximizing the information entropy of each region. Given its advantages—including computational simplicity, robust stability, and sensitivity to grayscale distribution—this paper adopts Otsu’s method as the objective function for multi-threshold image segmentation [22,23].

Assuming that grayscale image I comprises a total of L grayscale levels, and the number of pixels corresponding to the i-th grayscale level is $n_{i}$, then the total number of pixels in the image can be expressed as:

(18) $$N=\sum_{i=0}^{L-1}n_{i},$$

Accordingly, the probability of occurrence of the i-th grayscale level is:

(19) $$P_{i}=\frac{n_{i}}{N},i=0,1,\ldots ,L-1$$

where $P_{i}\geq 0$, and satisfying $\sum_{i=0}^{L-1}P_{i}=1$.

For single-threshold segmentation, when the gray value t is selected as the threshold, the image is divided into two parts: the target region and the background region. The gray value range [0, t] corresponds to the target region, and the gray value range [t+1, L−1] corresponds to the background region. Let the probabilities of the two regions be $\omega_{0}$ and $\omega_{1}$, and their average gray values be $\mu_{0}$ and $\mu_{1}$, respectively. The average gray value of the entire image is μ. Then:

(20) $$\omega_{0}=\sum_{i=0}^{t}P_{i}$$

(21) $$\mu_{0}=\frac{\sum_{i=0}^{t}iP_{i}}{\omega_{0}}$$

(22) $$\omega_{1}=\sum_{i=t+1}^{L-1}P_{i}$$

(23) $$\mu_{1}=\frac{\sum_{i=t+1}^{L-1}iP_{i}}{\omega_{1}}$$

(24) $$\mu =\sum_{i=0}^{L-1}iP_{i}$$

Based on this, the inter-class variance of single-threshold segmentation can be written as:

(25) $$\nu (t)=\omega_{0}(\mu_{0}-\mu {)}^{2}+\omega_{1}(\mu_{1}-\mu {)}^{2}$$

For multi-threshold segmentation, if k thresholds $t_{1},t_{2},\ldots ,t_{k}$ are set, the image will be divided into k+1 gray-level regions. Correspondingly, the inter-class variance in the multi-threshold case can be further extended as follows:

(26) $$\nu (t_{1},t_{2},\ldots ,t_{k})=\sum_{j=0}^{k}\omega_{j}(\mu_{j}-\mu {)}^{2}$$

Wherein, the probability and average gray level of the j-th region are defined as follows:

(27) $$\omega_{j}=\sum_{i=t_{j}+1}^{t_{j+1}}P_{i}$$

(28) $$\mu_{j}=\frac{\sum_{i=t_{j}+1}^{t_{j+1}}iP_{i}}{\omega_{j}}$$

Therefore, the multi-threshold image segmentation problem can be transformed into an optimization problem, namely, finding a set of optimal thresholds that maximizes the inter-class variance:

(29) $$T_{best}=\arg\max\nu (t_{1},t_{2},\ldots ,t_{k})$$

where $T_{best}$ represents the optimal combination of thresholds.

**Figure 14:**
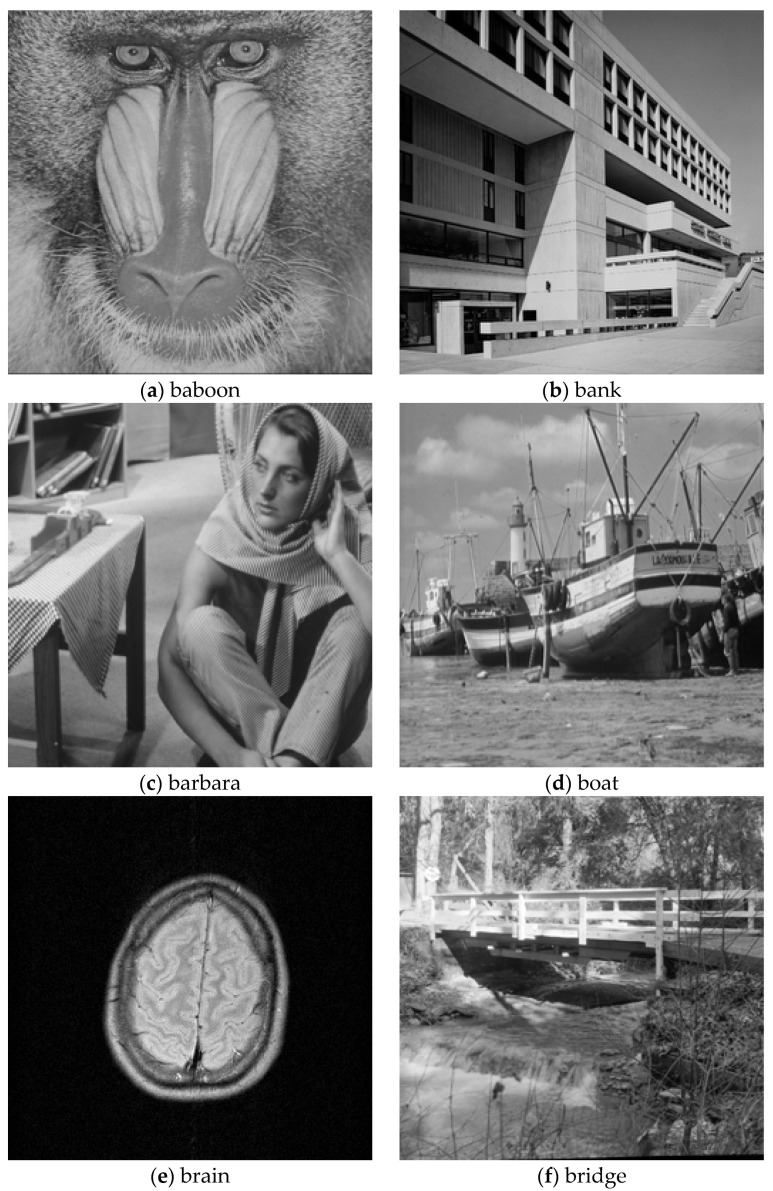

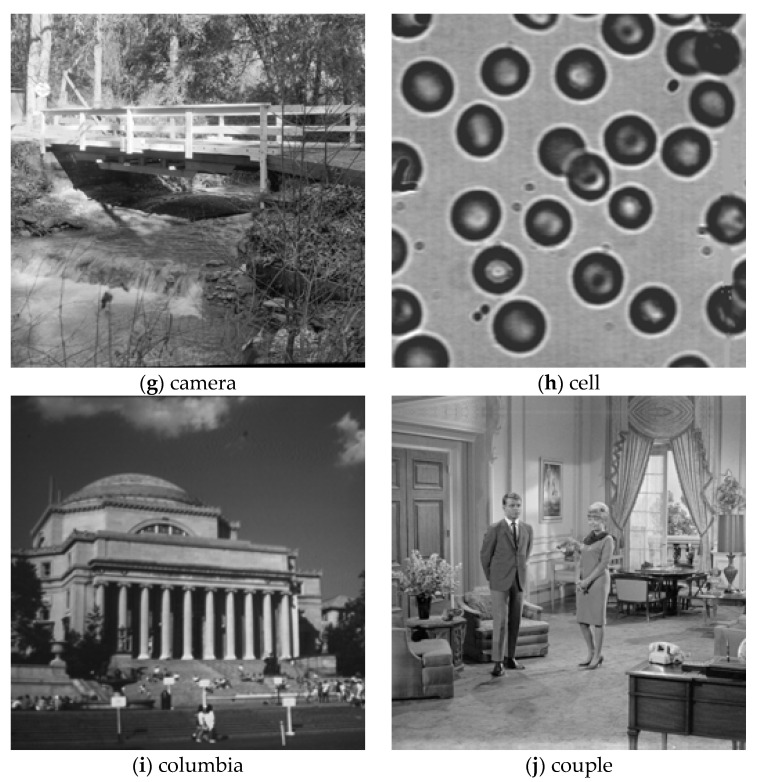
Benchmark Test Images Used for Segmentation Evaluation.

To verify the effectiveness of the proposed algorithm in multi-threshold image segmentation, this paper selects 10 typical test images for experimental analysis, as shown in Figure 14, and conducts comparative tests using different threshold numbers. All algorithms are run independently 30 times under the same parameter conditions to ensure the fairness and reliability of the experimental results. Finally, the segmentation accuracy and stability of the algorithm are comprehensively evaluated using the mean and standard deviation of the objective function values.

### 4.1. Evaluation Metrics

To comprehensively evaluate the quality of image segmentation results, this paper uses Peak Signal-to-Noise Ratio (PSNR), Structural Similarity Index (SSIM), and Feature Similarity Index (FSIM) as evaluation metrics.

PSNR measures the degree of distortion between the original image and the segmented image; a higher value indicates higher image fidelity. The definition of PSNR is as follows:

(30) $$PSNR=10{log}_{10}(\frac{255^{2}}{MSE}),$$

The mean squared error (MSE) can be expressed as:

(31) $$MSE=\frac{1}{MN}\sum_{j=1}^{M}\;\sum_{k=1}^{N}{\left[I(j,k)-I^{\prime }(j,k)\right]}^{2},$$

where M×N represents the image size, and I and $I^{\prime }$ represent the original image and the segmented image, respectively.

SSIM measures the similarity between two images in terms of brightness, contrast, and structural information. A value closer to 1 indicates a closer resemblance between the segmented image and the original image. The formula for calculating SSIM is:

(32) $$SSIM(I,I^{\prime })=\frac{\left(2\mu_{I}\mu_{I^{\prime }}+C_{1})(2\sigma_{{II}^{\prime }}+C_{2}\right)}{\left(\mu_{I}^{2}+\mu_{I^{\prime }}^{2}+C_{1})(\sigma_{I}^{2}+\sigma_{I^{\prime }}^{2}+C_{2}\right)},$$

where $\mu_{I}$ and $\mu_{I^{\prime }}$ represent the average gray values of the two images, respectively, $\sigma_{I}^{2}$ and $\sigma_{I^{\prime }}^{2}$ represent variance, $\sigma_{{II}^{\prime }}$ represents the covariance.

Unlike PSNR and SSIM, FSIM focuses more on edge, texture, and local feature information in an image, thus more effectively reflecting the preservation of image details. The expression for FSIM is:

(33) $$FSIM(I,I^{\prime })=\frac{\sum_{x\in \Omega }S_{L}(x)\cdot PC_{m}(x)}{\sum_{x\in \Omega }PC_{m}(x)},$$

where Ω represents the entire image region, $S_{L}(x)$ represents the local feature similarity at pixel x, and $PC_{m}(x)$ represents the phase consistency feature.

Overall, higher PSNR, SSIM, and FSIM values mean that the segmented image can better preserve the grayscale information, structural features, and details of the original image. Therefore, these metrics can comprehensively evaluate the performance of multi-threshold image segmentation algorithms.

### 4.2. MISBOA Analysis for Multilevel Threshold Segmentation Based on Otsu Criterion

This study applies the MISBOA to multilevel image thresholding and adopts the Otsu criterion as the objective function to search for the optimal threshold values for ten benchmark images. The segmentation performance of the proposed method is assessed by maximizing the Otsu objective function and further evaluated using three widely used image quality indicators, namely Peak Signal-to-Noise Ratio, Feature Similarity Index, and Structural Similarity Index. Larger values of these metrics indicate that the segmented image preserves more structural information, texture details, and visual quality from the original image. The experimental results are shown below.

**Figure 15:**
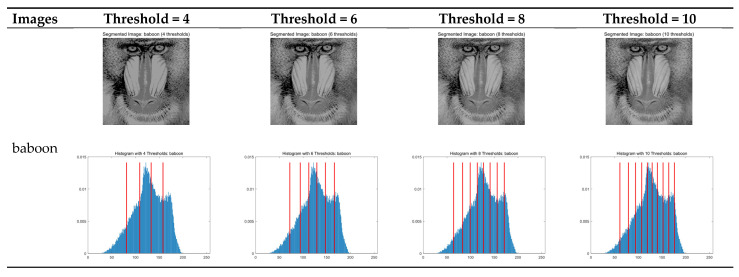

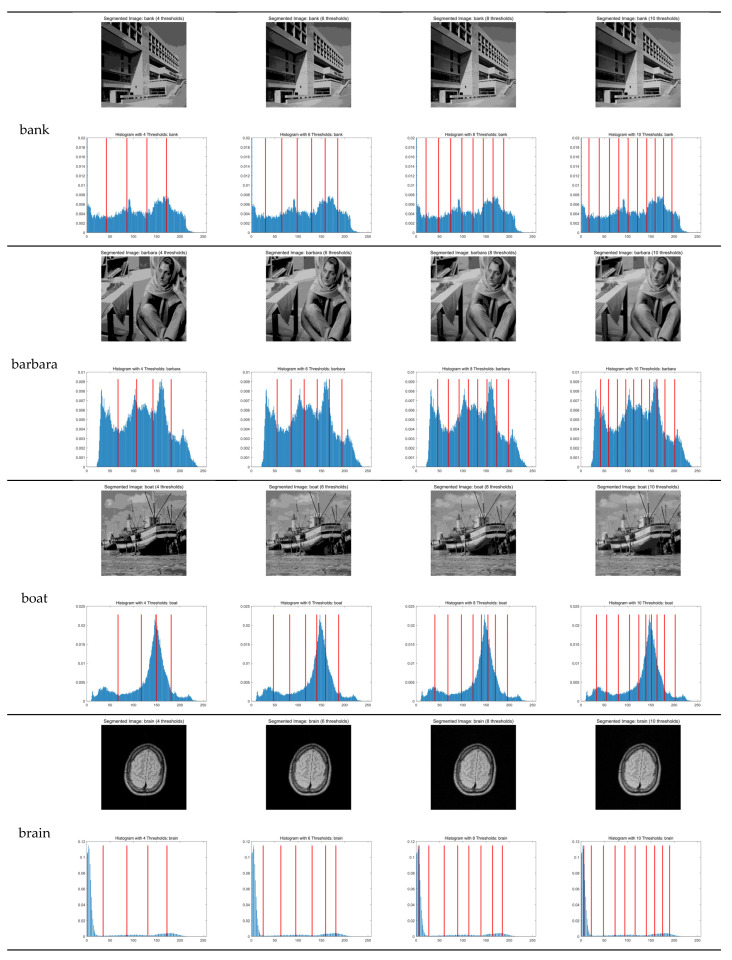

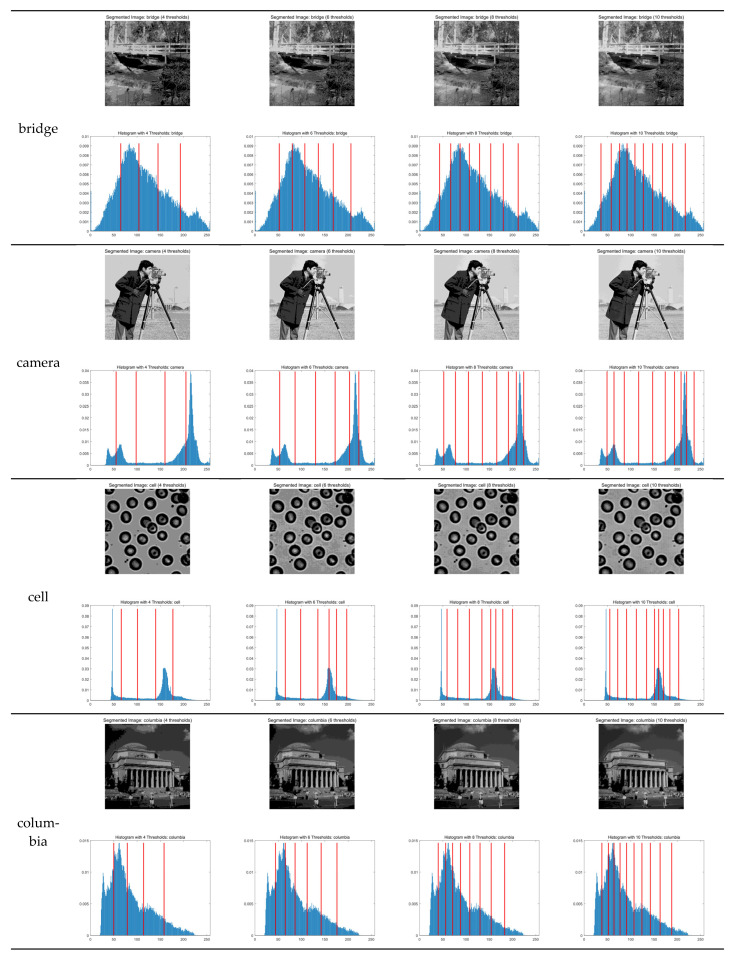

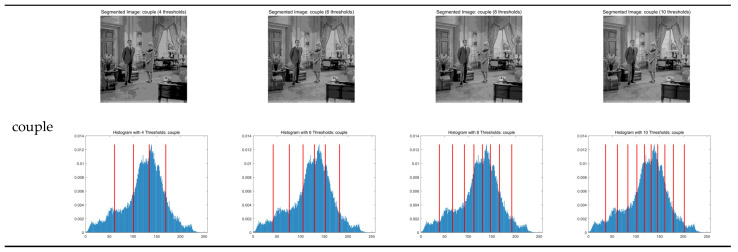
Performance of MISBOA in Otsu-Based Multilevel Threshold Segmentation.

Figure 15 shows the experimental results of MISBOA in the Otsu-based multi-threshold image segmentation task. As can be seen from the figure, with the increase in the number of thresholds, the target region, edge contours, and texture details in the image are gradually separated more clearly, indicating that MISBOA can effectively search for better threshold combinations, thereby improving segmentation quality. At lower threshold numbers, the segmentation results can already distinguish the foreground and background well and retain the main structural information of the image; as the number of thresholds further increases, the grayscale levels, local textures, and edge transitions in the image become richer and more natural, making the overall segmentation results more readable and visually continuous. For some test images with complex textures, uneven grayscale distribution, or blurred boundaries, MISBOA can still maintain the integrity of the target region well, reduce detail loss and over segmentation, demonstrating strong global search and local optimization capabilities. Overall, Figure 15 illustrates that MISBOA can obtain clear, stable, and visually high-quality segmentation results in the multi-threshold image segmentation task, verifying its effectiveness and practical value in complex image processing problems.

Table 11 presents the mean fitness (Ave) and standard deviation (Std) of different algorithms in the multi-threshold image segmentation task based on the Otsu objective function. Overall, the fitness values of each algorithm show an increasing trend with the increase in the number of thresholds, indicating that more thresholds can more fully exploit the gray-level distribution information in the image, thus achieving better segmentation results. However, compared with other comparative algorithms, MISBOA achieved a higher mean fitness value and a smaller standard deviation in most test images and under different threshold settings, indicating that it not only searches for better threshold combinations but also has better stability and consistency in multiple independent runs. Especially on images with complex textures, uneven gray-level distribution, and blurred edges, MISBOA’s advantage is more pronounced, with its Ave value significantly better than other algorithms, while its Std value remains at a low level. This shows that the proposed improvement strategy can effectively enhance population diversity, improve global search ability, and reduce the probability of the algorithm getting trapped in local optima. Overall, the experimental results in Table 11 fully demonstrate that MISBOA has higher optimization accuracy, stronger robustness, and better generalization ability in multi-threshold image segmentation tasks based on the Otsu objective function.

Table 12 presents the mean and standard deviation of PSNR for different algorithms on various test images in the Otsu multi-threshold segmentation task. Overall, the PSNR values of all algorithms generally improve with the increase in the number of thresholds, indicating that more thresholds can more fully preserve the grayscale information and detailed features in the original image, thereby reducing segmentation distortion. Compared to other comparison algorithms, MISBOA achieved higher PSNR values on most test images and under different threshold settings, with a smaller corresponding standard deviation, indicating that it can consistently obtain higher-quality segmentation results in multiple independent runs. Especially on images with complex textures, rich grayscale variations, and blurred edges, MISBOA’s advantages are more pronounced. Its higher PSNR value indicates that the segmented image better preserves the information of the original image, with lower pixel errors and less distortion. Meanwhile, the smaller Std value further demonstrates that MISBOA has good stability and robustness, and is not easily affected by random initialization and local optima. Overall, the results in Table 12 show that MISBOA achieves higher image reconstruction quality and better visual fidelity in Otsu-based multi-threshold image segmentation tasks.

Table 13 presents the mean and standard deviation of the FSIM scores for different algorithms on various test images in the Otsu multi-threshold image segmentation task. Overall, the FSIM scores of all algorithms generally improve with increasing threshold number, indicating that more thresholds better preserve edge, texture, and structural features in the image. Compared to other comparison algorithms, MISBOA achieved higher FSIM scores on most test images and under different threshold settings, with a smaller corresponding standard deviation. This suggests that it not only more effectively preserves important image features but also exhibits better stability and consistency in multiple independent runs. MISBOA’s advantage is particularly evident in images with rich textures, complex boundaries, and numerous details. Its higher FSIM score indicates that the segmentation results better preserve the edge contours, local textures, and visually salient features of the target region, making the segmented image more consistent with human visual perception. Meanwhile, the smaller standard deviation (Std) score further demonstrates that MISBOA performs more stably on complex images and is less susceptible to random factors. Overall, the experimental results in Table 13 show that MISBOA has stronger feature preservation capabilities and higher visual quality in Otsu-based multi-threshold image segmentation tasks.

Table 14 presents the mean and standard deviation of SSIM values for different algorithms on various test images in the Otsu multi-threshold image segmentation task. Overall, the SSIM values of all algorithms show an upward trend with increasing threshold number, indicating that a higher threshold number helps to more accurately preserve structural information and regional hierarchy in the image. Compared to other comparison algorithms, MISBOA achieved higher SSIM values on most test images and under different threshold settings, with a smaller standard deviation. This indicates that it not only better preserves the brightness, contrast, and structural features of the original image but also exhibits high stability and consistency in multiple independent runs. Especially in images with complex textures, blurred edges, and uneven grayscale distributions, MISBOA still maintains a high SSIM value, demonstrating that its segmentation results can more completely preserve the structural information of the target region, making the segmented image visually more natural and continuous. Meanwhile, the lower standard deviation further illustrates that MISBOA has good robustness in complex multi-threshold search spaces and is not easily affected by random initialization and local optima. Overall, the experimental results in Table 14 show that MISBOA has stronger structure preservation ability and better visual consistency in Otsu-based multi-threshold image segmentation tasks.

Table 15 presents the runtime comparison results of different algorithms on multiple benchmark images for multilevel threshold image segmentation tasks. Overall, MISBOA requires slightly more computational time than the original SBOA on most test images due to the introduction of multiple enhancement strategies, particularly the adaptive spiral Lévy flight and dynamic neighborhood-guided mutation mechanisms, which increase the computational complexity of the optimization process. However, the increase in runtime remains relatively limited and acceptable. For example, on most benchmark images, the runtime difference between MISBOA and SBOA is only a few seconds, while MISBOA achieves significantly better segmentation quality and optimization performance in terms of objective fitness values and image quality metrics. In addition, compared with several other algorithms such as ESC, GJA, and SSLO, MISBOA still maintains relatively competitive computational efficiency. Particularly, ESC exhibits substantially higher runtime on almost all benchmark images, whereas MISBOA achieves a better balance between segmentation accuracy and computational cost. Therefore, although MISBOA introduces a moderate increase in runtime compared with SBOA, this additional computational overhead is acceptable for multilevel threshold image segmentation problems considering the significant improvements in convergence accuracy, robustness, and segmentation quality achieved by the proposed algorithm.

## 5. Conclusions

In this paper, a Multi-Strategy Improved Secretary Bird Optimization Algorithm (MISBOA) is proposed to overcome the shortcomings of the original SBOA in terms of insufficient population diversity, weak local exploitation ability, and premature convergence. Three effective improvement strategies, namely chaotic elite initialization, adaptive spiral Lévy flight, and dynamic neighborhood-guided mutation, are incorporated into the original SBOA framework. These strategies significantly improve the balance between global exploration and local exploitation, enhance the ability to escape local optima, and strengthen the convergence accuracy and robustness of the algorithm.

To verify the performance of MISBOA, extensive experiments are conducted on the IEEE CEC2014, CEC2017, and CEC2020 benchmark suites. Compared with several advanced metaheuristic algorithms and representative CEC competition-winning algorithms, MISBOA achieves superior optimization accuracy, faster convergence speed, and stronger stability on most benchmark functions. In particular, MISBOA demonstrates highly competitive performance on complex multimodal, hybrid, and composition functions, indicating that the proposed strategies can effectively improve both exploration capability and exploitation performance. Furthermore, the convergence curves, boxplot analysis, statistical tests, strategy effectiveness analysis, and parameter sensitivity analysis consistently demonstrate that MISBOA possesses strong robustness, stable search behavior, and reliable optimization capability.

Furthermore, MISBOA is successfully applied to Otsu-based multilevel threshold image segmentation. Experimental results on multiple benchmark artistic images show that MISBOA can obtain higher objective fitness values and superior image quality metrics, including PSNR, FSIM, and SSIM. The segmented images also exhibit clearer object boundaries, richer detail preservation, and better visual effects. These findings confirm that MISBOA has strong applicability and effectiveness in solving complex artistic image segmentation problems.

Although MISBOA demonstrates promising optimization performance, several limitations still exist. First, the introduction of multiple enhancement strategies inevitably increases the computational complexity and computational overhead of the algorithm compared with the original SBOA. In particular, the dynamic neighborhood-guided mutation strategy requires additional neighborhood interaction calculations, which may increase the computational burden when solving high-dimensional optimization problems. Second, some control parameters in MISBOA are still empirically determined, and different optimization problems may require different parameter settings to achieve optimal performance. Therefore, the adaptive parameter adjustment capability of the algorithm still requires further investigation. Third, the current work mainly focuses on continuous numerical optimization and Otsu-based multilevel threshold image segmentation problems, while the performance of MISBOA on other challenging optimization scenarios, such as constrained optimization, multi-objective optimization, dynamic optimization, and discrete combinatorial optimization problems, has not yet been fully explored. In addition, although MISBOA exhibits strong optimization performance experimentally, deeper theoretical analysis and strict mathematical convergence proofs still remain insufficient.

Therefore, future work may focus on reducing the computational burden through adaptive neighborhood selection, parallel computing, or lightweight update mechanisms. In addition, MISBOA can be further extended to solve more real-world optimization problems, such as feature selection, engineering design, cloud task scheduling, wireless sensor network deployment, and UAV path planning. Future studies may also investigate adaptive parameter control mechanisms and theoretical convergence analysis to further improve the interpretability, scalability, and practical applicability of the proposed algorithm.

## Figures and Tables

**Table 1:** Parameter Configurations of the Compared Algorithms.

Algorithms	Parameter Name	Parameter Value	Reference
BHO	nTransfer, rho	5, 0.25	[32]
NDO	u	0.5	[33]
GJA	beta_start, beta_end	1.2, 0.3	[34]
SSLO	CR	0.9	[35]
LEE	inT, upr	1, 0.3	[36]
SAO	N1	0.5	[37]
RBMO	Epsilon	0.5	[38]
ESC	eliteSize, beta_base	5, 1.5	[39]
SBOA	beta	1.5	[25]

**Table 2:** Performance Comparison of Different Algorithms on the 30-Dimensional CEC2014 Benchmark Functions.

ID	Items	BHO	NDO	GJA	SSLO	LEE	SAO	RBMO	ESC	SBOA	MISBOA
CEC2014-F1	Ave	2.5066 × 10^5^	2.1732 × 10^6^	6.0964 × 10^6^	4.7766 × 10^7^	2.6614 × 10^6^	2.0733 × 10^6^	1.8911 × 10^5^	8.3092 × 10^6^	6.0607 × 10^6^	2.1767 × 10^3^
	Std	1.7409 × 10^5^	1.2405 × 10^6^	2.5346 × 10^6^	1.3085 × 10^7^	1.1783 × 10^6^	1.0938 × 10^6^	1.3707 × 10^5^	4.6988 × 10^6^	4.0035 × 10^6^	2.0669 × 10^3^
CEC2014-F2	Ave	2.0009 × 10^2^	3.2135 × 10^3^	1.3679 × 10^6^	1.0512 × 10^7^	2.0003 × 10^2^	1.0908 × 10^4^	2.0021 × 10^2^	9.2482 × 10^3^	8.7464 × 10^3^	2.0000 × 10^2^
	Std	2.2283 × 10^−1^	2.5095 × 10^3^	2.3735 × 10^5^	2.3774 × 10^6^	2.5235 × 10^−2^	9.9897 × 10^3^	2.7067 × 10^−1^	6.2854 × 10^3^	7.5205 × 10^3^	2.4186 × 10^−14^
CEC2014-F3	Ave	3.0044 × 10^2^	2.9023 × 10^3^	4.0530 × 10^3^	6.1515 × 10^3^	3.0002 × 10^2^	8.1133 × 10^3^	3.0025 × 10^2^	2.6203 × 10^3^	3.0248 × 10^3^	3.0000 × 10^2^
	Std	1.3791 × 10^00^	1.9766 × 10^3^	1.3393 × 10^3^	3.0504 × 10^3^	6.8199 × 10^−3^	3.7347 × 10^3^	2.3921 × 10^−1^	4.7131 × 10^3^	2.1803 × 10^3^	4.9510 × 10^−14^
CEC2014-F4	Ave	4.3863 × 10^2^	4.8282 × 10^2^	5.1429 × 10^2^	5.5740 × 10^2^	4.8438 × 10^2^	4.8809 × 10^2^	4.7285 × 10^2^	5.1470 × 10^2^	4.9407 × 10^2^	4.0011 × 10^2^
	Std	3.3035 × 10^1^	3.6196 × 10^1^	3.1459 × 10^1^	2.0410 × 10^1^	3.3222 × 10^1^	3.6493 × 10^1^	2.5577 × 10^1^	2.8831 × 10^1^	3.0176 × 10^1^	2.0105 × 10^−1^
CEC2014-F5	Ave	5.2016 × 10^2^	5.2057 × 10^2^	5.2093 × 10^2^	5.2048 × 10^2^	5.2054 × 10^2^	5.2100 × 10^2^	5.2030 × 10^2^	5.2097 × 10^2^	5.2012 × 10^2^	5.2074 × 10^2^
	Std	2.0395 × 10^−1^	1.7020 × 10^−1^	5.7840 × 10^−2^	5.3882 × 10^−2^	2.0339 × 10^−1^	6.2369 × 10^−2^	1.1585 × 10^−1^	5.0138 × 10^−2^	6.0937 × 10^−2^	9.6401 × 10^−2^
CEC2014-F6	Ave	6.0755 × 10^2^	6.1234 × 10^2^	6.1560 × 10^2^	6.2036 × 10^2^	6.0837 × 10^2^	6.0463 × 10^2^	6.0894 × 10^2^	6.0249 × 10^2^	6.1009 × 10^2^	6.0057 × 10^2^
	Std	4.0067 × 10^00^	2.7379 × 10^00^	1.9030 × 10^00^	1.2525 × 10^00^	3.0902 × 10^00^	2.1270 × 10^00^	3.8291 × 10^00^	1.2591 × 10^00^	2.6151 × 10^00^	7.9730 × 10^−1^
CEC2014-F7	Ave	7.0002 × 10^2^	7.0002 × 10^2^	7.0095 × 10^2^	7.0105 × 10^2^	7.0000 × 10^2^	7.0001 × 10^2^	7.0001 × 10^2^	7.0000 × 10^2^	7.0001 × 10^2^	7.0000 × 10^2^
	Std	1.3126 × 10^−2^	4.1455 × 10^−2^	3.4725 × 10^−2^	1.2865 × 10^−2^	4.3456 × 10^−3^	1.4565 × 10^−2^	8.0940 × 10^−3^	1.7810 × 10^−3^	1.5925 × 10^−2^	4.2222 × 10^−14^
CEC2014-F8	Ave	8.0141 × 10^2^	8.4968 × 10^2^	8.1184 × 10^2^	8.2880 × 10^2^	8.2534 × 10^2^	8.4458 × 10^2^	8.5284 × 10^2^	8.1038 × 10^2^	8.4521 × 10^2^	8.1386 × 10^2^
	Std	1.0506 × 10^00^	1.4750 × 10^1^	3.0762 × 10^00^	3.2199 × 10^00^	7.6367 × 10^00^	1.0673 × 10^1^	1.6732 × 10^1^	3.5213 × 10^00^	1.4511 × 10^1^	4.4337 × 10^00^
CEC2014-F9	Ave	9.2952 × 10^2^	9.8041 × 10^2^	9.6428 × 10^2^	1.0032 × 10^3^	9.5390 × 10^2^	9.5486 × 10^2^	9.4735 × 10^2^	9.7227 × 10^2^	9.6825 × 10^2^	9.1844 × 10^2^
	Std	6.8282 × 10^00^	1.8257 × 10^1^	1.6642 × 10^1^	1.0900 × 10^1^	1.4033 × 10^1^	1.3836 × 10^1^	1.3572 × 10^1^	2.2924 × 10^1^	1.3417 × 10^1^	7.4673 × 10^00^
CEC2014-F10	Ave	1.1430 × 10^3^	1.6611 × 10^3^	1.4575 × 10^3^	1.6655 × 10^3^	2.8879 × 10^3^	2.8874 × 10^3^	3.5463 × 10^3^	1.2096 × 10^3^	1.7793 × 10^3^	1.2235 × 10^3^
	Std	1.2284 × 10^2^	3.7858 × 10^2^	1.8627 × 10^2^	1.5438 × 10^2^	4.8829 × 10^2^	5.0340 × 10^2^	6.1863 × 10^2^	1.6878 × 10^2^	2.5914 × 10^2^	1.4805 × 10^2^
CEC2014-F11	Ave	3.5818 × 10^3^	4.4586 × 10^3^	3.6424 × 10^3^	4.7279 × 10^3^	4.6031 × 10^3^	3.9016 × 10^3^	4.5428 × 10^3^	6.3632 × 10^3^	3.9689 × 10^3^	3.3364 × 10^3^
	Std	4.6536 × 10^2^	7.3914 × 10^2^	6.7934 × 10^2^	2.9476 × 10^2^	6.5107 × 10^2^	7.3440 × 10^2^	4.4563 × 10^2^	4.8121 × 10^2^	7.0654 × 10^2^	6.1142 × 10^2^
CEC2014-F12	Ave	1.2004 × 10^3^	1.2006 × 10^3^	1.2006 × 10^3^	1.2007 × 10^3^	1.2005 × 10^3^	1.2017 × 10^3^	1.2008 × 10^3^	1.2021 × 10^3^	1.2002 × 10^3^	1.2005 × 10^3^
	Std	1.3763 × 10^−1^	2.5121 × 10^−1^	2.4100 × 10^−1^	1.3575 × 10^−1^	2.3348 × 10^−1^	1.0905 × 10^00^	3.1518 × 10^−1^	3.7220 × 10^−1^	1.1210 × 10^−1^	3.7475 × 10^−1^
CEC2014-F13	Ave	1.3002 × 10^3^	1.3005 × 10^3^	1.3004 × 10^3^	1.3004 × 10^3^	1.3004 × 10^3^	1.3005 × 10^3^	1.3003 × 10^3^	1.3003 × 10^3^	1.3004 × 10^3^	1.3002 × 10^3^
	Std	5.8921 × 10^−2^	2.7362 × 10^−1^	6.0189 × 10^−2^	4.3852 × 10^−2^	5.9542 × 10^−2^	8.7354 × 10^−2^	7.5534 × 10^−2^	4.4323 × 10^−2^	1.0615 × 10^−1^	3.5293 × 10^−2^
CEC2014-F14	Ave	1.4003 × 10^3^	1.4003 × 10^3^	1.4003 × 10^3^	1.4003 × 10^3^	1.4003 × 10^3^	1.4004 × 10^3^	1.4004 × 10^3^	1.4003 × 10^3^	1.4003 × 10^3^	1.4003 × 10^3^
	Std	5.7789 × 10^−2^	5.5809 × 10^−2^	4.7677 × 10^−2^	2.7024 × 10^−2^	8.2078 × 10^−2^	1.5322 × 10^−1^	2.2430 × 10^−1^	4.5337 × 10^−2^	1.0950 × 10^−1^	2.7868 × 10^−2^
CEC2014-F15	Ave	1.5042 × 10^3^	1.5092 × 10^3^	1.5184 × 10^3^	1.5177 × 10^3^	1.5061 × 10^3^	1.5159 × 10^3^	1.5052 × 10^3^	1.5098 × 10^3^	1.5071 × 10^3^	1.5031 × 10^3^
	Std	9.7598 × 10^−1^	3.4790 × 10^00^	3.6426 × 10^00^	1.7337 × 10^00^	1.7166 × 10^00^	3.8003 × 10^00^	1.3539 × 10^00^	1.1543 × 10^00^	2.3906 × 10^00^	6.9901 × 10^−1^
CEC2014-F16	Ave	1.6104 × 10^3^	1.6115 × 10^3^	1.6109 × 10^3^	1.6115 × 10^3^	1.6119 × 10^3^	1.6126 × 10^3^	1.6114 × 10^3^	1.6118 × 10^3^	1.6107 × 10^3^	1.6099 × 10^3^
	Std	5.6080 × 10^−1^	6.2709 × 10^−1^	5.4586 × 10^−1^	3.6655 × 10^−1^	6.1340 × 10^−1^	3.5099 × 10^−1^	6.6402 × 10^−1^	4.6880 × 10^−1^	7.4399 × 10^−1^	9.4974 × 10^−1^
CEC2014-F17	Ave	5.5852 × 10^3^	3.0809 × 10^5^	3.6616 × 10^5^	2.6380 × 10^6^	6.7220 × 10^3^	2.4155 × 10^5^	2.8542 × 10^3^	1.1021 × 10^6^	4.1504 × 10^5^	2.2941 × 10^3^
	Std	4.5609 × 10^3^	2.0216 × 10^5^	2.0292 × 10^5^	8.2519 × 10^5^	2.8337 × 10^3^	2.1127 × 10^5^	2.9209 × 10^2^	7.8913 × 10^5^	2.7741 × 10^5^	3.4118 × 10^2^
CEC2014-F18	Ave	1.9127 × 10^3^	3.9694 × 10^3^	2.9382 × 10^4^	4.6587 × 10^4^	2.2162 × 10^3^	4.1689 × 10^3^	3.1638 × 10^3^	3.3782 × 10^3^	4.1572 × 10^3^	1.8158 × 10^3^
	Std	4.5847 × 10^1^	2.3759 × 10^3^	8.0689 × 10^3^	4.4429 × 10^4^	8.7391 × 10^1^	4.0722 × 10^3^	5.0085 × 10^3^	1.2416 × 10^3^	3.2243 × 10^3^	1.8798 × 10^1^
CEC2014-F19	Ave	1.9077 × 10^3^	1.9145 × 10^3^	1.9111 × 10^3^	1.9130 × 10^3^	1.9093 × 10^3^	1.9102 × 10^3^	1.9114 × 10^3^	1.9075 × 10^3^	1.9085 × 10^3^	1.9037 × 10^3^
	Std	1.5887 × 10^00^	2.2969 × 10^1^	1.5497 × 10^00^	1.6027 × 10^00^	1.3737 × 10^1^	1.0218 × 10^1^	1.4669 × 10^1^	1.1062 × 10^1^	1.0651 × 10^1^	6.7297 × 10^−1^
CEC2014-F20	Ave	2.2132 × 10^3^	1.1512 × 10^4^	5.2819 × 10^3^	1.0811 × 10^4^	2.0948 × 10^3^	2.6618 × 10^4^	2.0820 × 10^3^	6.7316 × 10^3^	4.6816 × 10^3^	2.0069 × 10^3^
	Std	1.0351 × 10^2^	4.0277 × 10^3^	2.5286 × 10^3^	3.9214 × 10^3^	1.5978 × 10^1^	1.1507 × 10^4^	2.5385 × 10^1^	3.3594 × 10^3^	2.7647 × 10^3^	2.1031 × 10^00^
CEC2014-F21	Ave	2.9180 × 10^3^	8.4199 × 10^4^	1.4274 × 10^5^	4.0361 × 10^5^	4.3007 × 10^3^	1.5535 × 10^5^	2.9610 × 10^3^	2.4441 × 10^5^	1.1797 × 10^5^	2.3591 × 10^3^
	Std	2.9845 × 10^2^	7.0848 × 10^4^	1.1743 × 10^5^	1.3362 × 10^5^	4.0517 × 10^2^	9.8093 × 10^4^	2.3176 × 10^2^	2.1481 × 10^5^	1.2468 × 10^5^	1.2400 × 10^2^
CEC2014-F22	Ave	2.3974 × 10^3^	2.6189 × 10^3^	2.6263 × 10^3^	2.5957 × 10^3^	2.4759 × 10^3^	2.6471 × 10^3^	2.5076 × 10^3^	2.4037 × 10^3^	2.4288 × 10^3^	2.3616 × 10^3^
	Std	8.9222 × 10^1^	1.5423 × 10^2^	1.5234 × 10^2^	9.8016 × 10^1^	1.4043 × 10^2^	2.1090 × 10^2^	1.0856 × 10^2^	1.0767 × 10^2^	1.0711 × 10^2^	5.2308 × 10^1^
CEC2014-F23	Ave	2.5000 × 10^3^	2.6152 × 10^3^	2.6154 × 10^3^	2.6164 × 10^3^	2.5000 × 10^3^	2.6152 × 10^3^	2.6152 × 10^3^	2.6152 × 10^3^	2.6152 × 10^3^	2.6152 × 10^3^
	Std	0.0000 × 10^00^	2.8497 × 10^−7^	5.7549 × 10^−2^	3.6706 × 10^−1^	0.0000 × 10^00^	1.1860 × 10^−5^	7.4315 × 10^−8^	3.1751 × 10^−4^	9.0475 × 10^−7^	1.4964 × 10^−12^
CEC2014-F24	Ave	2.6000 × 10^3^	2.6384 × 10^3^	2.6264 × 10^3^	2.6384 × 10^3^	2.6000 × 10^3^	2.6086 × 10^3^	2.6328 × 10^3^	2.6275 × 10^3^	2.6000 × 10^3^	2.6244 × 10^3^
	Std	0.0000 × 10^00^	9.6732 × 10^00^	1.9696 × 10^00^	2.2380 × 10^00^	0.0000 × 10^00^	1.0735 × 10^1^	8.6256 × 10^00^	4.2284 × 10^00^	4.2152 × 10^−4^	1.4479 × 10^00^
CEC2014-F25	Ave	2.7000 × 10^3^	2.7087 × 10^3^	2.7097 × 10^3^	2.7129 × 10^3^	2.7000 × 10^3^	2.7033 × 10^3^	2.7043 × 10^3^	2.7073 × 10^3^	2.7015 × 10^3^	2.7030 × 10^3^
	Std	0.0000 × 10^00^	2.8358 × 10^00^	3.0918 × 10^00^	1.9317 × 10^00^	0.0000 × 10^00^	4.5657 × 10^00^	1.8730 × 10^00^	1.2664 × 10^00^	2.9335 × 10^00^	3.0097 × 10^−1^
CEC2014-F26	Ave	2.7036 × 10^3^	2.7042 × 10^3^	2.7635 × 10^3^	2.7004 × 10^3^	2.7003 × 10^3^	2.7237 × 10^3^	2.7225 × 10^3^	2.7069 × 10^3^	2.7303 × 10^3^	2.7002 × 10^3^
	Std	1.8209 × 10^1^	2.0533 × 10^1^	4.8873 × 10^1^	5.1958 × 10^−2^	6.5127 × 10^−2^	4.2809 × 10^1^	6.7846 × 10^1^	2.5378 × 10^1^	4.6428 × 10^1^	3.2830 × 10^−2^
CEC2014-F27	Ave	2.9000 × 10^3^	3.2097 × 10^3^	3.3272 × 10^3^	3.2543 × 10^3^	2.9429 × 10^3^	3.1839 × 10^3^	3.3264 × 10^3^	3.0923 × 10^3^	3.2220 × 10^3^	3.0209 × 10^3^
	Std	0.0000 × 10^00^	1.2366 × 10^2^	1.9481 × 10^2^	1.0923 × 10^2^	8.8021 × 10^1^	8.9489 × 10^1^	1.4673 × 10^2^	4.6584 × 10^1^	1.2048 × 10^2^	3.8252 × 10^1^
CEC2014-F28	Ave	3.2592 × 10^3^	3.8593 × 10^3^	3.9855 × 10^3^	3.7712 × 10^3^	3.0000 × 10^3^	3.8219 × 10^3^	3.9091 × 10^3^	3.6541 × 10^3^	3.6844 × 10^3^	3.6785 × 10^3^
	Std	2.3529 × 10^2^	2.4361 × 10^2^	4.3070 × 10^2^	2.7873 × 10^1^	0.0000 × 10^00^	2.3672 × 10^2^	2.0851 × 10^2^	4.4931 × 10^1^	6.2813 × 10^1^	2.3489 × 10^1^
CEC2014-F29	Ave	6.8506 × 10^4^	4.2437 × 10^3^	2.9974 × 10^5^	1.1505 × 10^4^	6.8265 × 10^5^	5.9885 × 10^5^	4.7270 × 10^6^	4.5430 × 10^3^	5.1207 × 10^3^	3.4499 × 10^3^
	Std	2.3017 × 10^5^	3.3950 × 10^2^	1.5902 × 10^6^	2.1707 × 10^4^	2.5939 × 10^6^	2.2620 × 10^6^	6.1732 × 10^6^	4.2237 × 10^2^	1.5422 × 10^3^	2.2320 × 10^2^
CEC2014-F30	Ave	5.0853 × 10^3^	5.5940 × 10^3^	6.2655 × 10^3^	1.3548 × 10^4^	6.1903 × 10^3^	6.0456 × 10^3^	6.9538 × 10^3^	6.8012 × 10^3^	6.0340 × 10^3^	3.6211 × 10^3^
	Std	4.2035 × 10^3^	9.2568 × 10^2^	8.2302 × 10^2^	2.7440 × 10^3^	1.1597 × 10^3^	1.0430 × 10^3^	2.5866 × 10^3^	2.6277 × 10^3^	9.8982 × 10^2^	2.0235 × 10^2^

**Table 3:** Performance Comparison of Different Algorithms on the 30-Dimensional CEC2017 Benchmark Functions.

ID	Items	BHO	NDO	GJA	SSLO	LEE	SAO	RBMO	ESC	SBOA	MISBOA
CEC2017-F1	Ave	1.0740 × 10^2^	3.6289 × 10^3^	1.2483 × 10^6^	1.2195 × 10^7^	1.6391 × 10^2^	4.2386 × 10^3^	4.0946 × 10^2^	2.8119 × 10^3^	5.2727 × 10^3^	1.0000 × 10^2^
	Std	1.4039 × 10^1^	4.1975 × 10^3^	2.0568 × 10^5^	4.0018 × 10^6^	6.8159 × 10^1^	5.8184 × 10^3^	3.4828 × 10^2^	3.6231 × 10^3^	5.3558 × 10^3^	2.6782 × 10^−14^
CEC2017-F3	Ave	3.0046 × 10^2^	5.7796 × 10^4^	3.1905 × 10^3^	1.0716 × 10^5^	1.0232 × 10^3^	7.1659 × 10^4^	3.0078 × 10^2^	4.4081 × 10^4^	5.9832 × 10^3^	3.0000 × 10^2^
	Std	4.3867 × 10^−1^	1.2400 × 10^4^	1.3429 × 10^3^	1.4848 × 10^4^	4.8830 × 10^2^	2.1870 × 10^4^	8.5749 × 10^−1^	1.3502 × 10^4^	3.1803 × 10^3^	3.2600 × 10^−9^
CEC2017-F4	Ave	4.6916 × 10^2^	4.7957 × 10^2^	5.0089 × 10^2^	5.3286 × 10^2^	4.9795 × 10^2^	4.9503 × 10^2^	4.7856 × 10^2^	5.0186 × 10^2^	4.9559 × 10^2^	4.2585 × 10^2^
	Std	2.5160 × 10^1^	3.4673 × 10^1^	2.3959 × 10^1^	1.2040 × 10^1^	1.4348 × 10^1^	1.4288 × 10^1^	2.4333 × 10^1^	2.3444 × 10^1^	2.3162 × 10^1^	2.8065 × 10^1^
CEC2017-F5	Ave	5.2875 × 10^2^	5.8186 × 10^2^	5.6398 × 10^2^	6.0096 × 10^2^	5.4852 × 10^2^	5.5250 × 10^2^	5.4794 × 10^2^	5.7739 × 10^2^	5.6455 × 10^2^	5.2189 × 10^2^
	Std	6.5946 × 10^00^	4.6020 × 10^1^	1.9621 × 10^1^	1.0160 × 10^1^	1.3792 × 10^1^	1.2937 × 10^1^	1.0442 × 10^1^	1.9085 × 10^1^	1.5616 × 10^1^	6.3789 × 10^00^
CEC2017-F6	Ave	6.0028 × 10^2^	6.0241 × 10^2^	6.0563 × 10^2^	6.0266 × 10^2^	6.0000 × 10^2^	6.0004 × 10^2^	6.0004 × 10^2^	6.0000 × 10^2^	6.0055 × 10^2^	6.0000 × 10^2^
	Std	2.3263 × 10^−1^	2.9305 × 10^00^	1.9400 × 10^00^	3.3107 × 10^−1^	2.0635 × 10^−4^	6.1913 × 10^−2^	5.3407 × 10^−2^	4.2105 × 10^−3^	6.1343 × 10^−1^	1.3378 × 10^−6^
CEC2017-F7	Ave	7.6558 × 10^2^	8.3293 × 10^2^	8.0812 × 10^2^	8.9383 × 10^2^	7.8168 × 10^2^	8.2386 × 10^2^	7.7783 × 10^2^	8.2475 × 10^2^	8.0460 × 10^2^	7.4829 × 10^2^
	Std	9.4642 × 10^00^	2.8546 × 10^1^	1.5932 × 10^1^	1.2263 × 10^1^	1.4760 × 10^1^	6.3526 × 10^1^	1.3283 × 10^1^	1.5747 × 10^1^	2.8026 × 10^1^	6.1372 × 10^00^
CEC2017-F8	Ave	8.2869 × 10^2^	8.7289 × 10^2^	8.6388 × 10^2^	9.0264 × 10^2^	8.5423 × 10^2^	8.5255 × 10^2^	8.5372 × 10^2^	8.7526 × 10^2^	8.5984 × 10^2^	8.1834 × 10^2^
	Std	7.4096 × 10^00^	1.9211 × 10^1^	1.5371 × 10^1^	1.0846 × 10^1^	1.4316 × 10^1^	1.4265 × 10^1^	1.7585 × 10^1^	2.1218 × 10^1^	1.7338 × 10^1^	4.3853 × 10^00^
CEC2017-F9	Ave	9.2509 × 10^2^	1.2225 × 10^3^	9.7483 × 10^2^	1.6693 × 10^3^	9.0152 × 10^2^	9.0546 × 10^2^	9.0890 × 10^2^	9.0084 × 10^2^	9.5482 × 10^2^	9.0003 × 10^2^
	Std	2.6963 × 10^1^	3.3651 × 10^2^	4.1124 × 10^1^	1.7538 × 10^2^	3.5595 × 10^00^	1.2454 × 10^1^	1.4554 × 10^1^	1.5142 × 10^00^	8.4825 × 10^1^	8.6397 × 10^−2^
CEC2017-F10	Ave	3.8951 × 10^3^	4.8517 × 10^3^	4.0983 × 10^3^	4.8336 × 10^3^	4.4588 × 10^3^	3.8680 × 10^3^	4.4890 × 10^3^	6.4250 × 10^3^	4.2045 × 10^3^	3.2613 × 10^3^
	Std	4.8122 × 10^2^	6.9530 × 10^2^	6.7577 × 10^2^	3.8861 × 10^2^	6.2092 × 10^2^	5.0078 × 10^2^	6.0145 × 10^2^	4.8994 × 10^2^	5.8371 × 10^2^	5.3609 × 10^2^
CEC2017-F11	Ave	1.1998 × 10^3^	1.1783 × 10^3^	1.2064 × 10^3^	1.6102 × 10^3^	1.1671 × 10^3^	1.1665 × 10^3^	1.1596 × 10^3^	1.1741 × 10^3^	1.1764 × 10^3^	1.1211 × 10^3^
	Std	3.2665 × 10^1^	3.3516 × 10^1^	3.4591 × 10^1^	1.6262 × 10^2^	3.0603 × 10^1^	4.6103 × 10^1^	3.0849 × 10^1^	2.4623 × 10^1^	3.3387 × 10^1^	2.1412 × 10^1^
CEC2017-F12	Ave	2.7334 × 10^4^	1.0588 × 10^5^	2.3382 × 10^6^	5.2432 × 10^6^	1.7946 × 10^5^	3.2335 × 10^5^	1.8770 × 10^4^	7.1805 × 10^5^	3.4547 × 10^5^	2.7938 × 10^3^
	Std	1.8891 × 10^4^	8.7463 × 10^4^	1.2377 × 10^6^	2.2234 × 10^6^	2.6087 × 10^5^	2.7853 × 10^5^	1.4759 × 10^4^	6.2879 × 10^5^	3.1649 × 10^5^	7.8023 × 10^2^
CEC2017-F13	Ave	3.5923 × 10^3^	1.2230 × 10^4^	1.0536 × 10^5^	1.9841 × 10^5^	7.0239 × 10^3^	1.8695 × 10^4^	4.5262 × 10^3^	1.1995 × 10^4^	2.0372 × 10^4^	1.3489 × 10^3^
	Std	1.2176 × 10^3^	1.0232 × 10^4^	3.0763 × 10^4^	1.8661 × 10^5^	5.9008 × 10^3^	1.8662 × 10^4^	1.0995 × 10^4^	9.4711 × 10^3^	2.0948 × 10^4^	3.6688 × 10^1^
CEC2017-F14	Ave	1.5448 × 10^3^	1.6923 × 10^4^	1.4493 × 10^4^	9.8324 × 10^4^	1.5059 × 10^3^	2.4081 × 10^4^	1.4594 × 10^3^	4.9109 × 10^4^	1.8495 × 10^4^	1.4282 × 10^3^
	Std	6.1477 × 10^1^	1.2532 × 10^4^	1.2729 × 10^4^	6.9423 × 10^4^	1.0893 × 10^1^	1.9232 × 10^4^	1.3063 × 10^1^	5.9674 × 10^4^	1.7469 × 10^4^	3.6274 × 10^00^
CEC2017-F15	Ave	1.7486 × 10^3^	8.7618 × 10^3^	3.3355 × 10^4^	4.0356 × 10^4^	1.7647 × 10^3^	5.5854 × 10^3^	1.6751 × 10^3^	1.0353 × 10^4^	1.1047 × 10^4^	1.5143 × 10^3^
	Std	1.1110 × 10^2^	7.6780 × 10^3^	1.4281 × 10^4^	2.9036 × 10^4^	6.0349 × 10^1^	7.5008 × 10^3^	6.4461 × 10^1^	8.5785 × 10^3^	8.3287 × 10^3^	6.7724 × 10^00^
CEC2017-F16	Ave	1.9866 × 10^3^	2.4280 × 10^3^	2.4762 × 10^3^	2.5923 × 10^3^	2.2445 × 10^3^	2.3497 × 10^3^	2.2201 × 10^3^	2.0926 × 10^3^	2.2408 × 10^3^	2.0377 × 10^3^
	Std	2.0850 × 10^2^	3.0132 × 10^2^	2.2409 × 10^2^	1.3612 × 10^2^	2.6898 × 10^2^	2.6690 × 10^2^	1.8825 × 10^2^	2.1612 × 10^2^	2.2161 × 10^2^	2.3614 × 10^2^
CEC2017-F17	Ave	1.8056 × 10^3^	2.0274 × 10^3^	1.9327 × 10^3^	1.9934 × 10^3^	1.9042 × 10^3^	2.0287 × 10^3^	1.9497 × 10^3^	1.8140 × 10^3^	1.8556 × 10^3^	1.7439 × 10^3^
	Std	4.6432 × 10^1^	1.7959 × 10^2^	1.0746 × 10^2^	8.1197 × 10^1^	1.1721 × 10^2^	2.0915 × 10^2^	9.7818 × 10^1^	8.1015 × 10^1^	8.2337 × 10^1^	3.8824 × 10^1^
CEC2017-F18	Ave	3.5039 × 10^3^	2.4241 × 10^5^	2.3512 × 10^5^	6.8135 × 10^5^	4.9840 × 10^3^	3.8670 × 10^5^	1.9144 × 10^3^	5.5822 × 10^5^	2.6816 × 10^5^	1.8752 × 10^3^
	Std	1.6423 × 10^3^	2.3965 × 10^5^	2.3267 × 10^5^	3.3020 × 10^5^	3.2704 × 10^3^	4.3688 × 10^5^	3.3725 × 10^1^	6.4101 × 10^5^	1.4387 × 10^5^	4.4420 × 10^1^
CEC2017-F19	Ave	2.0563 × 10^3^	1.0845 × 10^4^	4.5153 × 10^4^	5.1731 × 10^4^	1.9893 × 10^3^	6.4958 × 10^3^	1.9441 × 10^3^	1.2806 × 10^4^	1.3130 × 10^4^	1.9129 × 10^3^
	Std	6.4774 × 10^1^	1.2607 × 10^4^	2.7267 × 10^4^	3.2759 × 10^4^	2.8480 × 10^1^	3.7784 × 10^3^	1.7652 × 10^1^	1.3053 × 10^4^	1.5142 × 10^4^	4.4667 × 10^00^
CEC2017-F20	Ave	2.1398 × 10^3^	2.3182 × 10^3^	2.2774 × 10^3^	2.3562 × 10^3^	2.2589 × 10^3^	2.3239 × 10^3^	2.2698 × 10^3^	2.1269 × 10^3^	2.2297 × 10^3^	2.0891 × 10^3^
	Std	6.1937 × 10^1^	1.4433 × 10^2^	1.1556 × 10^2^	9.3653 × 10^1^	1.0974 × 10^2^	1.7134 × 10^2^	1.2085 × 10^2^	6.6802 × 10^1^	1.0293 × 10^2^	6.1402 × 10^1^
CEC2017-F21	Ave	2.3304 × 10^3^	2.3706 × 10^3^	2.3624 × 10^3^	2.3982 × 10^3^	2.3464 × 10^3^	2.3581 × 10^3^	2.3575 × 10^3^	2.3727 × 10^3^	2.3461 × 10^3^	2.3188 × 10^3^
	Std	6.7739 × 10^00^	2.1658 × 10^1^	1.5823 × 10^1^	1.2376 × 10^1^	1.3204 × 10^1^	1.6117 × 10^1^	1.7772 × 10^1^	1.9821 × 10^1^	1.1288 × 10^1^	6.8398 × 10^00^
CEC2017-F22	Ave	2.3003 × 10^3^	2.4268 × 10^3^	2.3120 × 10^3^	2.3240 × 10^3^	3.4112 × 10^3^	2.8764 × 10^3^	4.3287 × 10^3^	3.3803 × 10^3^	2.3008 × 10^3^	2.3000 × 10^3^
	Std	1.0373 × 10^00^	5.3421 × 10^2^	7.9133 × 10^−1^	5.6797 × 10^00^	1.7464 × 10^3^	1.1937 × 10^3^	1.9617 × 10^3^	2.2038 × 10^3^	1.2847 × 10^00^	1.6889 × 10^−13^
CEC2017-F23	Ave	2.6854 × 10^3^	2.7337 × 10^3^	2.7251 × 10^3^	2.7465 × 10^3^	2.7041 × 10^3^	2.7049 × 10^3^	2.7253 × 10^3^	2.7068 × 10^3^	2.6996 × 10^3^	2.6600 × 10^3^
	Std	8.5479 × 10^00^	2.3965 × 10^1^	2.1614 × 10^1^	1.1534 × 10^1^	1.3833 × 10^1^	1.3782 × 10^1^	2.6433 × 10^1^	2.2609 × 10^1^	1.6947 × 10^1^	1.0466 × 10^1^
CEC2017-F24	Ave	2.8560 × 10^3^	2.9096 × 10^3^	2.8888 × 10^3^	2.9149 × 10^3^	2.8783 × 10^3^	2.8798 × 10^3^	2.8995 × 10^3^	2.9132 × 10^3^	2.8692 × 10^3^	2.8320 × 10^3^
	Std	1.0336 × 10^1^	2.6077 × 10^1^	1.8817 × 10^1^	1.4235 × 10^1^	1.8662 × 10^1^	1.6584 × 10^1^	3.2291 × 10^1^	1.4565 × 10^1^	1.5848 × 10^1^	5.8100 × 10^00^
CEC2017-F25	Ave	2.8893 × 10^3^	2.8908 × 10^3^	2.9135 × 10^3^	2.9207 × 10^3^	2.8873 × 10^3^	2.8867 × 10^3^	2.8892 × 10^3^	2.8889 × 10^3^	2.8933 × 10^3^	2.8869 × 10^3^
	Std	6.6324 × 10^00^	9.7651 × 10^00^	1.8745 × 10^1^	8.8034 × 10^00^	8.2619 × 10^−1^	1.8729 × 10^00^	6.6018 × 10^00^	4.3333 × 10^00^	1.2431 × 10^1^	1.0303 × 10^−1^
CEC2017-F26	Ave	3.4460 × 10^3^	4.1009 × 10^3^	3.6682 × 10^3^	4.6996 × 10^3^	4.2180 × 10^3^	4.1323 × 10^3^	4.3594 × 10^3^	3.9437 × 10^3^	3.7868 × 10^3^	3.6386 × 10^3^
	Std	5.8271 × 10^2^	8.9344 × 10^2^	9.8808 × 10^2^	1.5191 × 10^2^	1.3092 × 10^2^	3.7319 × 10^2^	4.9309 × 10^2^	1.5738 × 10^2^	6.3072 × 10^2^	1.0213 × 10^2^
CEC2017-F27	Ave	3.2135 × 10^3^	3.2407 × 10^3^	3.2421 × 10^3^	3.2345 × 10^3^	3.2141 × 10^3^	3.2227 × 10^3^	3.2239 × 10^3^	3.2173 × 10^3^	3.2108 × 10^3^	3.2090 × 10^3^
	Std	1.0289 × 10^1^	1.9179 × 10^1^	1.8671 × 10^1^	7.1712 × 10^00^	1.1141 × 10^1^	1.3915 × 10^1^	2.2498 × 10^1^	8.2055 × 10^00^	1.1409 × 10^1^	1.1028 × 10^1^
CEC2017-F28	Ave	3.1712 × 10^3^	3.2288 × 10^3^	3.2447 × 10^3^	3.2882 × 10^3^	3.2284 × 10^3^	3.2159 × 10^3^	3.3235 × 10^3^	3.2243 × 10^3^	3.2196 × 10^3^	3.1335 × 10^3^
	Std	5.7453 × 10^1^	1.0445 × 10^2^	2.0971 × 10^1^	1.1244 × 10^1^	2.6018 × 10^1^	1.8035 × 10^1^	6.0940 × 10^2^	1.7275 × 10^1^	2.3788 × 10^1^	5.2481 × 10^1^
CEC2017-F29	Ave	3.4945 × 10^3^	3.6068 × 10^3^	3.6311 × 10^3^	3.6685 × 10^3^	3.6090 × 10^3^	3.6583 × 10^3^	3.6456 × 10^3^	3.4485 × 10^3^	3.5081 × 10^3^	3.3630 × 10^3^
	Std	9.7275 × 10^1^	1.5391 × 10^2^	1.8566 × 10^2^	6.5626 × 10^1^	1.3069 × 10^2^	1.3517 × 10^2^	1.3415 × 10^2^	7.4082 × 10^1^	1.1446 × 10^2^	1.9823 × 10^1^
CEC2017-F30	Ave	7.3532 × 10^3^	9.3334 × 10^3^	1.6175 × 10^5^	9.5922 × 10^4^	1.7642 × 10^4^	8.5175 × 10^3^	7.0105 × 10^3^	1.0911 × 10^4^	2.0223 × 10^4^	5.3068 × 10^3^
	Std	2.2403 × 10^3^	3.0397 × 10^3^	9.9323 × 10^4^	3.5134 × 10^4^	5.8532 × 10^3^	3.6042 × 10^3^	1.6945 × 10^3^	4.4873 × 10^3^	2.4201 × 10^4^	1.3538 × 10^2^

**Table 4:** Performance Comparison of Different Algorithms on the 100-Dimensional CEC2017 Benchmark Functions.

ID	Items	BHO	NDO	GJA	SSLO	LEE	SAO	RBMO	ESC	SBOA	MISBOA
CEC2017-F1	Ave	2.3656 × 10^5^	2.1626 × 10^8^	2.8232 × 10^7^	1.3296 × 10^10^	4.6316 × 10^4^	1.4426 × 10^8^	3.9710 × 10^7^	8.6900 × 10^9^	2.3778 × 10^8^	6.7162 × 10^3^
	Std	1.9031 × 10^5^	4.8936 × 10^8^	3.2417 × 10^6^	1.0328 × 10^9^	9.7020 × 10^4^	6.7772 × 10^7^	2.6587 × 10^7^	3.4946 × 10^9^	5.0375 × 10^8^	5.6948 × 10^3^
CEC2017-F3	Ave	1.1238 × 10^5^	4.9515 × 10^5^	2.9307 × 10^5^	5.6949 × 10^5^	2.3626 × 10^5^	7.9730 × 10^5^	7.9903 × 10^4^	4.8855 × 10^5^	2.4441 × 10^5^	3.1722 × 10^5^
	Std	1.8219 × 10^4^	5.6885 × 10^4^	4.2376 × 10^4^	4.9903 × 10^4^	2.7139 × 10^4^	1.7806 × 10^5^	1.5551 × 10^4^	5.0067 × 10^4^	2.3827 × 10^4^	2.2250 × 10^4^
CEC2017-F4	Ave	7.1858 × 10^2^	9.2884 × 10^2^	9.2122 × 10^2^	2.5730 × 10^3^	7.4919 × 10^2^	7.6995 × 10^2^	8.5117 × 10^2^	1.4783 × 10^3^	9.5445 × 10^2^	6.4263 × 10^2^
	Std	5.6997 × 10^1^	7.6884 × 10^1^	6.5320 × 10^1^	1.2746 × 10^2^	6.0462 × 10^1^	5.1533 × 10^1^	5.8474 × 10^1^	2.7996 × 10^2^	8.7135 × 10^1^	5.0854 × 10^1^
CEC2017-F5	Ave	8.3803 × 10^2^	9.7909 × 10^2^	9.5883 × 10^2^	1.3573 × 10^3^	8.3476 × 10^2^	1.2069 × 10^3^	9.3365 × 10^2^	1.0478 × 10^3^	9.9938 × 10^2^	6.2995 × 10^2^
	Std	5.4671 × 10^1^	7.0918 × 10^1^	8.2643 × 10^1^	3.7609 × 10^1^	5.9760 × 10^1^	2.3507 × 10^2^	7.0651 × 10^1^	1.2999 × 10^2^	7.5345 × 10^1^	2.1719 × 10^1^
CEC2017-F6	Ave	6.1784 × 10^2^	6.2240 × 10^2^	6.2534 × 10^2^	6.2600 × 10^2^	6.0807 × 10^2^	6.1170 × 10^2^	6.1111 × 10^2^	6.0584 × 10^2^	6.2608 × 10^2^	6.0115 × 10^2^
	Std	3.8183 × 10^00^	5.4665 × 10^00^	3.6385 × 10^00^	1.1616 × 10^00^	3.8598 × 10^00^	3.0288 × 10^00^	3.4458 × 10^00^	1.3344 × 10^00^	5.5798 × 10^00^	4.4594 × 10^−1^
CEC2017-F7	Ave	1.5294 × 10^3^	1.7855 × 10^3^	1.4494 × 10^3^	2.3961 × 10^3^	1.2976 × 10^3^	1.9557 × 10^3^	1.3352 × 10^3^	1.5178 × 10^3^	1.9246 × 10^3^	9.1940 × 10^2^
	Std	1.2869 × 10^2^	1.8724 × 10^2^	6.5704 × 10^1^	4.2081 × 10^1^	7.2251 × 10^1^	5.0828 × 10^1^	8.3361 × 10^1^	1.1265 × 10^2^	2.0701 × 10^2^	2.3951 × 10^1^
CEC2017-F8	Ave	1.1862 × 10^3^	1.3151 × 10^3^	1.2322 × 10^3^	1.6595 × 10^3^	1.1113 × 10^3^	1.4270 × 10^3^	1.2227 × 10^3^	1.3644 × 10^3^	1.2882 × 10^3^	9.3150 × 10^2^
	Std	6.1575 × 10^1^	8.5719 × 10^1^	6.1181 × 10^1^	3.2115 × 10^1^	5.1768 × 10^1^	2.1662 × 10^2^	1.0006 × 10^2^	1.1717 × 10^2^	6.3832 × 10^1^	2.1689 × 10^1^
CEC2017-F9	Ave	9.1382 × 10^3^	2.6044 × 10^4^	1.3531 × 10^4^	2.9839 × 10^4^	6.5359 × 10^3^	9.0870 × 10^3^	6.1915 × 10^3^	5.3717 × 10^3^	1.7130 × 10^4^	9.4629 × 10^2^
	Std	2.3868 × 10^3^	9.0571 × 10^3^	5.6651 × 10^3^	4.1849 × 10^3^	1.6765 × 10^3^	2.8502 × 10^3^	2.3205 × 10^3^	1.6787 × 10^3^	3.2980 × 10^3^	3.4376 × 10^1^
CEC2017-F10	Ave	1.5224 × 10^4^	2.3979 × 10^4^	1.4171 × 10^4^	2.1661 × 10^4^	1.5521 × 10^4^	2.0339 × 10^4^	1.9326 × 10^4^	2.8178 × 10^4^	1.4382 × 10^4^	2.9217 × 10^4^
	Std	1.1596 × 10^3^	4.0393 × 10^3^	1.1554 × 10^3^	5.2214 × 10^2^	1.2388 × 10^3^	6.9978 × 10^3^	1.7473 × 10^3^	6.6470 × 10^2^	1.8747 × 10^3^	8.9907 × 10^2^
CEC2017-F11	Ave	2.6851 × 10^3^	5.7817 × 10^4^	3.7572 × 10^3^	1.1568 × 10^5^	1.5462 × 10^4^	1.2635 × 10^5^	3.4177 × 10^3^	4.2050 × 10^4^	1.2155 × 10^4^	2.6260 × 10^3^
	Std	1.7968 × 10^2^	1.9018 × 10^4^	7.5299 × 10^2^	1.5916 × 10^4^	4.2986 × 10^3^	3.7462 × 10^4^	3.4655 × 10^2^	9.7885 × 10^3^	4.7945 × 10^3^	1.8456 × 10^2^
CEC2017-F12	Ave	7.4148 × 10^6^	4.1636 × 10^7^	1.7013 × 10^8^	3.3919 × 10^9^	2.6075 × 10^7^	4.4456 × 10^7^	3.2439 × 10^7^	3.6758 × 10^8^	5.4397 × 10^7^	3.6697 × 10^6^
	Std	2.8801 × 10^6^	2.5223 × 10^7^	5.2121 × 10^7^	3.6296 × 10^8^	1.0571 × 10^7^	2.6693 × 10^7^	2.7889 × 10^7^	2.5097 × 10^8^	2.6916 × 10^7^	1.5510 × 10^6^
CEC2017-F13	Ave	1.3917 × 10^4^	8.9109 × 10^3^	8.3288 × 10^5^	3.2118 × 10^7^	6.3794 × 10^3^	8.0810 × 10^3^	1.5315 × 10^4^	3.4792 × 10^4^	1.0632 × 10^4^	4.0617 × 10^3^
	Std	6.0828 × 10^3^	7.1554 × 10^3^	1.0378 × 10^5^	1.0888 × 10^7^	4.8554 × 10^3^	5.4849 × 10^3^	2.3616 × 10^4^	1.1822 × 10^4^	9.4458 × 10^3^	2.2374 × 10^3^
CEC2017-F14	Ave	5.6774 × 10^4^	1.0487 × 10^6^	1.5424 × 10^6^	1.6347 × 10^7^	4.0947 × 10^5^	8.3518 × 10^5^	2.3522 × 10^3^	4.7472 × 10^6^	1.7399 × 10^6^	2.5560 × 10^4^
	Std	3.5301 × 10^4^	5.6758 × 10^5^	4.2439 × 10^5^	4.5129 × 10^6^	2.2547 × 10^5^	4.1761 × 10^5^	1.9075 × 10^2^	3.1375 × 10^6^	9.3930 × 10^5^	1.7086 × 10^4^
CEC2017-F15	Ave	6.8646 × 10^3^	5.2421 × 10^3^	3.2655 × 10^5^	2.1906 × 10^6^	6.0308 × 10^3^	5.0891 × 10^3^	6.7004 × 10^3^	1.6607 × 10^4^	6.3186 × 10^3^	2.5555 × 10^3^
	Std	6.9871 × 10^3^	3.5998 × 10^3^	5.1891 × 10^4^	1.2305 × 10^6^	5.9219 × 10^3^	4.1559 × 10^3^	7.5220 × 10^3^	5.4254 × 10^3^	5.0903 × 10^3^	1.0446 × 10^3^
CEC2017-F16	Ave	4.5108 × 10^3^	5.2095 × 10^3^	5.8084 × 10^3^	7.9535 × 10^3^	5.3750 × 10^3^	5.1321 × 10^3^	6.0382 × 10^3^	6.7069 × 10^3^	4.9341 × 10^3^	3.9377 × 10^3^
	Std	6.7589 × 10^2^	7.7525 × 10^2^	6.2921 × 10^2^	2.7254 × 10^2^	7.2387 × 10^2^	6.2585 × 10^2^	7.5143 × 10^2^	7.5548 × 10^2^	7.1780 × 10^2^	6.2776 × 10^2^
CEC2017-F17	Ave	4.2193 × 10^3^	4.6872 × 10^3^	5.3200 × 10^3^	6.3280 × 10^3^	4.8059 × 10^3^	5.1044 × 10^3^	5.1280 × 10^3^	5.0645 × 10^3^	4.5074 × 10^3^	3.7065 × 10^3^
	Std	5.2659 × 10^2^	5.4104 × 10^2^	5.8614 × 10^2^	2.5693 × 10^2^	5.0609 × 10^2^	1.0927 × 10^3^	5.5306 × 10^2^	4.5268 × 10^2^	4.6538 × 10^2^	3.8564 × 10^2^
CEC2017-F18	Ave	1.7430 × 10^5^	5.0972 × 10^6^	1.7135 × 10^6^	1.8128 × 10^7^	7.9193 × 10^5^	3.4358 × 10^6^	8.7935 × 10^4^	8.3247 × 10^6^	3.4329 × 10^6^	7.8461 × 10^4^
	Std	7.4403 × 10^4^	3.0340 × 10^6^	5.1663 × 10^5^	4.8052 × 10^6^	4.1902 × 10^5^	2.1150 × 10^6^	3.9863 × 10^4^	3.4027 × 10^6^	1.7623 × 10^6^	1.9852 × 10^4^
CEC2017-F19	Ave	6.4859 × 10^3^	5.7996 × 10^3^	5.9772 × 10^5^	4.6921 × 10^6^	6.0335 × 10^3^	4.9609 × 10^3^	6.9109 × 10^3^	2.5038 × 10^4^	8.6746 × 10^3^	3.3521 × 10^3^
	Std	6.2478 × 10^3^	4.7900 × 10^3^	1.6030 × 10^5^	2.7171 × 10^6^	6.1895 × 10^3^	3.0320 × 10^3^	5.4175 × 10^3^	4.6720 × 10^4^	9.0864 × 10^3^	1.3850 × 10^3^
CEC2017-F20	Ave	4.5133 × 10^3^	5.6774 × 10^3^	4.6167 × 10^3^	6.0873 × 10^3^	5.0227 × 10^3^	5.4992 × 10^3^	4.9890 × 10^3^	5.7320 × 10^3^	4.3832 × 10^3^	4.3067 × 10^3^
	Std	3.9588 × 10^2^	6.5777 × 10^2^	4.8729 × 10^2^	2.7658 × 10^2^	4.7156 × 10^2^	1.4038 × 10^3^	5.0777 × 10^2^	3.3659 × 10^2^	5.1315 × 10^2^	6.5664 × 10^2^
CEC2017-F21	Ave	2.7132 × 10^3^	2.8101 × 10^3^	2.7456 × 10^3^	3.1871 × 10^3^	2.6387 × 10^3^	2.9009 × 10^3^	2.8686 × 10^3^	2.7763 × 10^3^	2.7793 × 10^3^	2.4481 × 10^3^
	Std	6.8494 × 10^1^	6.4857 × 10^1^	5.9494 × 10^1^	3.3741 × 10^1^	5.0480 × 10^1^	2.1268 × 10^2^	1.0823 × 10^2^	1.4014 × 10^2^	5.8507 × 10^1^	2.0727 × 10^1^
CEC2017-F22	Ave	1.9147 × 10^4^	2.3964 × 10^4^	1.4600 × 10^4^	2.3618 × 10^4^	1.8503 × 10^4^	2.2047 × 10^4^	2.2662 × 10^4^	2.9752 × 10^4^	1.8465 × 10^4^	3.0181 × 10^4^
	Std	3.4351 × 10^3^	3.8542 × 10^3^	5.7353 × 10^3^	2.3026 × 10^3^	1.2258 × 10^3^	6.9765 × 10^3^	1.7331 × 10^3^	9.3367 × 10^2^	1.8855 × 10^3^	4.6507 × 10^3^
CEC2017-F23	Ave	3.2901 × 10^3^	3.3057 × 10^3^	3.2787 × 10^3^	3.5467 × 10^3^	3.1765 × 10^3^	3.1796 × 10^3^	3.5807 × 10^3^	3.1279 × 10^3^	3.2147 × 10^3^	3.0198 × 10^3^
	Std	6.1841 × 10^1^	8.6901 × 10^1^	6.0468 × 10^1^	2.3372 × 10^1^	4.8020 × 10^1^	5.6195 × 10^1^	1.6950 × 10^2^	5.1835 × 10^1^	5.0010 × 10^1^	3.2517 × 10^1^
CEC2017-F24	Ave	3.8708 × 10^3^	3.9951 × 10^3^	3.6959 × 10^3^	4.1662 × 10^3^	3.6581 × 10^3^	3.6391 × 10^3^	4.2663 × 10^3^	3.6577 × 10^3^	3.7900 × 10^3^	3.4339 × 10^3^
	Std	1.1900 × 10^2^	1.1000 × 10^2^	7.3386 × 10^1^	3.2190 × 10^1^	6.7183 × 10^1^	6.4127 × 10^1^	2.0348 × 10^2^	8.3090 × 10^1^	9.1430 × 10^1^	3.3121 × 10^1^
CEC2017-F25	Ave	3.3821 × 10^3^	3.6049 × 10^3^	3.6035 × 10^3^	5.6725 × 10^3^	3.4252 × 10^3^	3.4859 × 10^3^	3.5348 × 10^3^	4.3815 × 10^3^	3.6110 × 10^3^	3.2703 × 10^3^
	Std	5.1848 × 10^1^	9.8536 × 10^1^	6.9321 × 10^1^	1.6037 × 10^2^	6.6191 × 10^1^	5.0934 × 10^1^	7.9807 × 10^1^	3.0052 × 10^2^	1.2027 × 10^2^	5.0850 × 10^1^
CEC2017-F26	Ave	1.1720 × 10^4^	1.4015 × 10^4^	1.4567 × 10^4^	1.5412 × 10^4^	1.0052 × 10^4^	9.4798 × 10^3^	1.3063 × 10^4^	9.6390 × 10^3^	1.3782 × 10^4^	7.0934 × 10^3^
	Std	1.2317 × 10^3^	2.0469 × 10^3^	5.8420 × 10^3^	3.6692 × 10^2^	7.5648 × 10^2^	5.1247 × 10^2^	1.7015 × 10^3^	6.5037 × 10^2^	3.2203 × 10^3^	2.3801 × 10^2^
CEC2017-F27	Ave	3.6557 × 10^3^	3.7558 × 10^3^	3.7740 × 10^3^	3.8946 × 10^3^	3.5658 × 10^3^	3.4287 × 10^3^	3.5226 × 10^3^	3.7109 × 10^3^	3.5892 × 10^3^	3.5068 × 10^3^
	Std	9.4630 × 10^1^	1.0374 × 10^2^	1.2417 × 10^2^	6.0549 × 10^1^	9.9748 × 10^1^	4.0292 × 10^1^	6.2107 × 10^1^	7.9100 × 10^1^	7.5451 × 10^1^	3.6778 × 10^1^
CEC2017-F28	Ave	3.4883 × 10^3^	3.8545 × 10^3^	3.7399 × 10^3^	6.9463 × 10^3^	3.5473 × 10^3^	3.5151 × 10^3^	3.7818 × 10^3^	6.2668 × 10^3^	3.7350 × 10^3^	3.4021 × 10^3^
	Std	3.9728 × 10^1^	1.7091 × 10^2^	5.7352 × 10^1^	4.3816 × 10^2^	3.0065 × 10^1^	3.4859 × 10^1^	1.4084 × 10^2^	7.9983 × 10^2^	5.8356 × 10^1^	3.7373 × 10^1^
CEC2017-F29	Ave	6.2833 × 10^3^	6.4310 × 10^3^	7.2054 × 10^3^	8.2914 × 10^3^	6.8524 × 10^3^	6.0556 × 10^3^	7.2724 × 10^3^	6.1302 × 10^3^	6.3823 × 10^3^	5.1616 × 10^3^
	Std	5.1510 × 10^2^	4.4050 × 10^2^	4.8165 × 10^2^	3.1281 × 10^2^	5.2648 × 10^2^	5.5642 × 10^2^	6.2413 × 10^2^	7.1563 × 10^2^	5.1835 × 10^2^	3.6989 × 10^2^
CEC2017-F30	Ave	5.1238 × 10^4^	1.8996 × 10^5^	4.0863 × 10^6^	2.1047 × 10^7^	9.0194 × 10^4^	2.7021 × 10^4^	7.0792 × 10^4^	6.2361 × 10^6^	5.7704 × 10^4^	1.1205 × 10^4^
	Std	3.7408 × 10^4^	1.7521 × 10^5^	1.1122 × 10^6^	4.3527 × 10^6^	4.1427 × 10^4^	2.1326 × 10^4^	1.2400 × 10^5^	1.1453 × 10^7^	4.9220 × 10^4^	2.8422 × 10^3^

**Table 5:** Performance Comparison of Different Algorithms on the 10-Dimensional CEC2020 Benchmark Functions.

ID	Items	BHO	NDO	GJA	SSLO	LEE	SAO	RBMO	ESC	SBOA	MISBOA
CEC2020-F1	Ave	1.0000 × 10^2^	2.0320 × 10^3^	5.1276 × 10^4^	1.7130 × 10^3^	1.0000 × 10^2^	1.4940 × 10^3^	1.0000 × 10^2^	1.4128 × 10^3^	3.4987 × 10^3^	1.0000 × 10^2^
	Std	0.0000 × 10^00^	2.2764 × 10^3^	1.5061 × 10^4^	1.4145 × 10^3^	6.1731 × 10^−6^	1.7886 × 10^3^	1.0998 × 10^−9^	1.8063 × 10^3^	3.8100 × 10^3^	0.0000 × 10^00^
CEC2020-F2	Ave	1.1673 × 10^3^	1.3691 × 10^3^	1.3519 × 10^3^	1.2821 × 10^3^	1.5156 × 10^3^	1.5442 × 10^3^	1.3842 × 10^3^	1.2153 × 10^3^	1.3187 × 10^3^	1.1353 × 10^3^
	Std	6.6420 × 10^1^	1.9253 × 10^2^	1.6758 × 10^2^	8.7577 × 10^1^	1.9987 × 10^2^	2.4909 × 10^2^	1.4950 × 10^2^	9.5647 × 10^1^	1.5524 × 10^2^	6.7669 × 10^1^
CEC2020- F3	Ave	7.1302 × 10^2^	7.2386 × 10^2^	7.1878 × 10^2^	7.2011 × 10^2^	7.1867 × 10^2^	7.1790 × 10^2^	7.1610 × 10^2^	7.1495 × 10^2^	7.1926 × 10^2^	7.1432 × 10^2^
	Std	1.1134 × 10^00^	6.2820 × 10^00^	3.5085 × 10^00^	2.6383 × 10^00^	4.3009 × 10^00^	3.3847 × 10^00^	4.0017 × 10^00^	1.8063 × 10^00^	5.2250 × 10^00^	1.9631 × 10^00^
CEC2020- F4	Ave	1.9005 × 10^3^	1.9011 × 10^3^	1.9009 × 10^3^	1.9011 × 10^3^	1.9008 × 10^3^	1.9009 × 10^3^	1.9007 × 10^3^	1.9011 × 10^3^	1.9009 × 10^3^	1.9006 × 10^3^
	Std	2.1196 × 10^−1^	5.2379 × 10^−1^	3.8904 × 10^−1^	2.2809 × 10^−1^	2.2350 × 10^−1^	3.4985 × 10^−1^	1.8610 × 10^−1^	2.0396 × 10^−1^	3.1218 × 10^−1^	2.0031 × 10^−1^
CEC2020- F5	Ave	1.8181 × 10^3^	5.4815 × 10^3^	3.1354 × 10^3^	1.2650 × 10^4^	1.7566 × 10^3^	3.3108 × 10^3^	1.7488 × 10^3^	5.1592 × 10^3^	2.9188 × 10^3^	1.7079 × 10^3^
	Std	9.0924 × 10^1^	2.9586 × 10^3^	1.2181 × 10^3^	6.7002 × 10^3^	4.0437 × 10^1^	1.1072 × 10^3^	3.3008 × 10^1^	3.0830 × 10^3^	1.1159 × 10^3^	9.0020 × 10^00^
CEC2020- F6	Ave	1.6170 × 10^3^	1.6874 × 10^3^	1.7296 × 10^3^	1.6031 × 10^3^	1.6012 × 10^3^	1.6875 × 10^3^	1.6282 × 10^3^	1.6179 × 10^3^	1.6214 × 10^3^	1.6005 × 10^3^
	Std	4.0928 × 10^1^	7.9769 × 10^1^	8.2648 × 10^1^	1.6116 × 10^00^	3.3895 × 10^−1^	9.4651 × 10^1^	5.1459 × 10^1^	3.9963 × 10^1^	4.5224 × 10^1^	2.3636 × 10^−1^
CEC2020- F7	Ave	2.1107 × 10^3^	2.4271 × 10^3^	3.5007 × 10^3^	2.8563 × 10^3^	2.1179 × 10^3^	8.4079 × 10^3^	2.1129 × 10^3^	2.6306 × 10^3^	2.2323 × 10^3^	2.1003 × 10^3^
	Std	2.5176 × 10^1^	1.2338 × 10^3^	1.2997 × 10^3^	6.1264 × 10^2^	8.9514 × 10^00^	4.9000 × 10^3^	1.5142 × 10^1^	8.6007 × 10^2^	1.1022 × 10^2^	2.8797 × 10^−1^
CEC2020- F8	Ave	2.2941 × 10^3^	2.2992 × 10^3^	2.3042 × 10^3^	2.2974 × 10^3^	2.2922 × 10^3^	2.2982 × 10^3^	2.2978 × 10^3^	2.3003 × 10^3^	2.2941 × 10^3^	2.2933 × 10^3^
	Std	2.3273 × 10^1^	1.4046 × 10^1^	9.9570 × 10^−1^	1.0513 × 10^1^	2.6286 × 10^1^	1.3058 × 10^1^	1.6289 × 10^1^	2.6509 × 10^−1^	2.4049 × 10^1^	2.5371 × 10^1^
CEC2020- F9	Ave	2.6007 × 10^3^	2.6307 × 10^3^	2.7046 × 10^3^	2.6949 × 10^3^	2.6995 × 10^3^	2.7444 × 10^3^	2.7141 × 10^3^	2.7366 × 10^3^	2.7063 × 10^3^	2.6076 × 10^3^
	Std	1.1717 × 10^2^	1.1994 × 10^2^	8.1504 × 10^1^	6.3528 × 10^1^	9.0782 × 10^1^	6.1567 × 10^00^	7.2693 × 10^1^	2.6107 × 10^00^	8.2351 × 10^1^	1.1705 × 10^2^
CEC2020- F10	Ave	2.9178 × 10^3^	2.9268 × 10^3^	2.9231 × 10^3^	2.8942 × 10^3^	2.9091 × 10^3^	2.9366 × 10^3^	2.9140 × 10^3^	2.9351 × 10^3^	2.9122 × 10^3^	2.9133 × 10^3^
	Std	2.2831 × 10^1^	2.3330 × 10^1^	2.2924 × 10^1^	3.3719 × 10^1^	2.0275 × 10^1^	1.9358 × 10^1^	2.2738 × 10^1^	2.0629 × 10^1^	2.1337 × 10^1^	2.2138 × 10^1^

**Table 6:** Performance Comparison of Different Algorithms on the 20-Dimensional CEC2020 Benchmark Functions.

ID	Items	BHO	NDO	GJA	SSLO	LEE	SAO	RBMO	ESC	SBOA	MISBOA
CEC2020-F1	Ave	1.0000 × 10^2^	2.6474 × 10^3^	3.6400 × 10^5^	9.6632 × 10^4^	1.0003 × 10^2^	1.8602 × 10^3^	1.0006 × 10^2^	1.4788 × 10^3^	3.8620 × 10^3^	1.0000 × 10^2^
	Std	5.2596 × 10^−7^	2.6801 × 10^3^	6.0721 × 10^4^	4.9678 × 10^4^	3.3184 × 10^−2^	2.5999 × 10^3^	9.5109 × 10^−2^	1.6985 × 10^3^	3.8546 × 10^3^	2.6389 × 10^−15^
CEC2020-F2	Ave	1.3133 × 10^3^	1.6728 × 10^3^	1.7697 × 10^3^	1.7812 × 10^3^	2.3493 × 10^3^	2.1489 × 10^3^	2.4181 × 10^3^	1.3455 × 10^3^	1.8534 × 10^3^	1.2239 × 10^3^
	Std	1.2688 × 10^2^	2.7333 × 10^2^	3.1742 × 10^2^	1.3158 × 10^2^	5.4236 × 10^2^	4.3716 × 10^2^	3.6818 × 10^2^	2.1398 × 10^2^	3.2082 × 10^2^	1.1880 × 10^2^
CEC2020- F3	Ave	7.2467 × 10^2^	7.6072 × 10^2^	7.5152 × 10^2^	7.5446 × 10^2^	7.4120 × 10^2^	7.4191 × 10^2^	7.4805 × 10^2^	7.3167 × 10^2^	7.4779 × 10^2^	7.2780 × 10^2^
	Std	1.9301 × 10^00^	2.9888 × 10^1^	7.1785 × 10^00^	5.3007 × 10^00^	7.8036 × 10^00^	7.4569 × 10^00^	8.3167 × 10^00^	4.7777 × 10^00^	1.1321 × 10^1^	3.5604 × 10^00^
CEC2020- F4	Ave	1.9012 × 10^3^	1.9034 × 10^3^	1.9060 × 10^3^	1.9038 × 10^3^	1.9028 × 10^3^	1.9025 × 10^3^	1.9025 × 10^3^	1.9033 × 10^3^	1.9027 × 10^3^	1.9015 × 10^3^
	Std	2.8079 × 10^−1^	1.8456 × 10^00^	1.6494 × 10^00^	4.6540 × 10^−1^	1.1288 × 10^00^	1.8444 × 10^00^	9.1032 × 10^−1^	6.5264 × 10^−1^	9.5541 × 10^−1^	3.4225 × 10^−1^
CEC2020- F5	Ave	2.5462 × 10^3^	8.7376 × 10^4^	9.7698 × 10^4^	4.3548 × 10^5^	2.5373 × 10^3^	1.0054 × 10^5^	2.1211 × 10^3^	2.2152 × 10^5^	1.1113 × 10^5^	1.8584 × 10^3^
	Std	3.1032 × 10^2^	5.9723 × 10^4^	3.4071 × 10^4^	2.4844 × 10^5^	2.5393 × 10^2^	5.0959 × 10^4^	1.2771 × 10^2^	1.7251 × 10^5^	9.7544 × 10^4^	7.7977 × 10^1^
CEC2020- F6	Ave	1.6032 × 10^3^	1.7148 × 10^3^	1.6912 × 10^3^	1.6114 × 10^3^	1.6182 × 10^3^	1.8301 × 10^3^	1.7152 × 10^3^	1.6133 × 10^3^	1.6486 × 10^3^	1.6023 × 10^3^
	Std	2.2062 × 10^00^	9.8260 × 10^1^	7.8896 × 10^1^	3.0660 × 10^00^	1.0292 × 10^1^	1.6252 × 10^2^	1.4309 × 10^2^	2.2822 × 10^1^	7.2413 × 10^1^	1.5090 × 10^00^
CEC2020- F7	Ave	2.3531 × 10^3^	8.9650 × 10^4^	4.8649 × 10^4^	1.0608 × 10^5^	2.7291 × 10^3^	9.2191 × 10^4^	2.4673 × 10^3^	8.4762 × 10^4^	3.0750 × 10^4^	2.1406 × 10^3^
	Std	1.3704 × 10^2^	8.6398 × 10^4^	3.4140 × 10^4^	6.3115 × 10^4^	1.8521 × 10^2^	8.6143 × 10^4^	1.1016 × 10^2^	8.5509 × 10^4^	3.2334 × 10^4^	5.1018 × 10^1^
CEC2020- F8	Ave	2.3004 × 10^3^	2.3008 × 10^3^	2.3081 × 10^3^	2.3019 × 10^3^	2.5158 × 10^3^	2.3537 × 10^3^	2.4607 × 10^3^	2.4741 × 10^3^	2.3003 × 10^3^	2.3000 × 10^3^
	Std	5.5270 × 10^−1^	7.0819 × 10^−1^	8.8076 × 10^−1^	5.3323 × 10^−1^	6.6153 × 10^2^	2.9269 × 10^2^	6.1192 × 10^2^	6.6256 × 10^2^	6.1521 × 10^−1^	1.1942 × 10^−13^
CEC2020- F9	Ave	2.8122 × 10^3^	2.8420 × 10^3^	2.8279 × 10^3^	2.8326 × 10^3^	2.8284 × 10^3^	2.8295 × 10^3^	2.8368 × 10^3^	2.8315 × 10^3^	2.8224 × 10^3^	2.8050 × 10^3^
	Std	5.2927 × 10^00^	2.3999 × 10^1^	1.2236 × 10^1^	6.0194 × 10^00^	1.1552 × 10^1^	1.1981 × 10^1^	1.2564 × 10^1^	1.1963 × 10^1^	1.2817 × 10^1^	4.0310 × 10^00^
CEC2020- F10	Ave	2.9146 × 10^3^	2.9511 × 10^3^	2.9701 × 10^3^	2.9176 × 10^3^	2.9219 × 10^3^	2.9259 × 10^3^	2.9246 × 10^3^	2.9211 × 10^3^	2.9342 × 10^3^	2.9136 × 10^3^
	Std	6.8313 × 10^00^	3.3864 × 10^1^	2.6325 × 10^1^	4.1139 × 10^00^	2.2081 × 10^1^	2.3885 × 10^1^	2.3990 × 10^1^	1.7529 × 10^1^	2.8985 × 10^1^	6.6357 × 10^−1^

**Table 7:** Experimental Results of the Wilcoxon Signed-Rank Test. (W/T/L).

MISBOA.VS	BHO	NDO	GJA	SSLO	LEE	SAO	RBMO	ESC	SBOA
CEC2014 (dim = 30)	23/7/0	27/2/1	28/2/0	28/1/1	22/2/6	27/2/1	28/1/1	25/4/1	23/3/4
CEC2017 (dim = 30)	27/2/1	30/0/0	29/1/0	30/0/0	29/1/0	29/1/0	29/1/0	28/2/0	28/2/0
CEC2017 (dim = 100)	24/3/3	28/0/2	26/1/3	28/0/2	25/2/3	26/1/3	26/2/2	28/1/1	26/1/3
CEC2020 (dim = 10)	5/3/2	9/1/0	9/1/0	8/2/0	8/2/0	10/0/0	8/2/0	10/0/0	9/1/0
CEC2020 (dim = 20)	7/1/2	10/0/0	10/0/0	10/0/0	10/0/0	9/1/0	10/0/0	9/1/0	10/0/0

**Table 8:** Friedman mean rank test result.

Suites	CEC2014		CEC2017				CEC2020			
Dimensions	30		30		100		10		20	
Algorithms	M.R	T.R	M.R	T.R	M.R	T.R	M.R	T.R	M.R	T.R
BHO	2.30	2	3.10	2	3.90	3	2.20	2	2.10	2
NDO	6.43	7	6.67	8	6.73	8	7.50	8	7.00	8
GJA	7.47	9	7.60	9	6.17	7	7.90	10	7.80	9
SSLO	8.40	10	9.77	10	9.70	10	7.10	7	8.40	10
LEE	4.37	3	4.53	3	3.67	2	5.30	4	5.00	3
SAO	7.00	8	5.50	5	5.27	5	7.70	9	6.20	7
RBMO	5.33	4	4.53	3	5.20	4	4.80	3	5.70	4
ESC	6.17	6	6.57	7	7.00	9	5.60	5	5.90	6
SBOA	5.53	5	5.70	6	5.50	6	5.60	5	5.70	4
MISBOA	2.00	1	1.03	1	1.87	1	1.30	1	1.20	1

**Table 9:** Experimental results compared with CEC winner algorithm.

Algorithm	Items	LSHADE	LSHADE-SPACMA	LSHADE-cnEpSin	MISBOA
CEC2017-F1	Ave	1.0708 × 10^2^	1.0001 × 10^2^	1.7712 × 10^3^	1.0000 × 10^2^
	Std	1.7515 × 10^1^	4.8689 × 10^−2^	2.4661 × 10^3^	2.0270 × 10^−14^
CEC2017-F3	Ave	4.3045 × 10^3^	3.5032 × 10^4^	3.9070 × 10^3^	3.0000 × 10^2^
	Std	2.0679 × 10^4^	5.4744 × 10^4^	6.6174 × 10^3^	1.8533 × 10^−10^
CEC2017-F4	Ave	4.6797 × 10^2^	4.5903 × 10^2^	4.8859 × 10^2^	4.3582 × 10^2^
	Std	2.1733 × 10^1^	3.3214 × 10^1^	2.8736 × 10^1^	2.6922 × 10^1^
CEC2017-F5	Ave	5.2977 × 10^2^	5.3351 × 10^2^	5.3480 × 10^2^	5.2040 × 10^2^
	Std	4.8004 × 10^00^	5.2945 × 10^00^	6.2344 × 10^00^	5.3405 × 10^00^
CEC2017-F6	Ave	6.0009 × 10^2^	6.0035 × 10^2^	6.0067 × 10^2^	6.0000 × 10^2^
	Std	1.1217 × 10^−1^	3.5811 × 10^−1^	4.8942 × 10^−1^	1.9196 × 10^−6^
CEC2017-F7	Ave	7.6497 × 10^2^	7.6628 × 10^2^	7.6962 × 10^2^	7.4610 × 10^2^
	Std	7.7942 × 10^00^	8.9243 × 10^00^	9.1815 × 10^00^	4.7083 × 10^00^
CEC2017-F8	Ave	8.2823 × 10^2^	8.3177 × 10^2^	8.3493 × 10^2^	8.1987 × 10^2^
	Std	6.3110 × 10^00^	8.2330 × 10^00^	5.2959 × 10^00^	5.8919 × 10^00^
CEC2017-F9	Ave	9.2084 × 10^2^	9.2396 × 10^2^	9.2642 × 10^2^	9.0001 × 10^2^
	Std	1.5364 × 10^1^	2.9162 × 10^1^	2.3478 × 10^1^	2.7318 × 10^−2^
CEC2017-F10	Ave	3.5280 × 10^3^	3.7472 × 10^3^	3.7694 × 10^3^	3.3798 × 10^3^
	Std	3.0783 × 10^2^	4.4807 × 10^2^	3.3263 × 10^2^	5.7834 × 10^2^
CEC2017-F11	Ave	1.1932 × 10^3^	1.2112 × 10^3^	1.2193 × 10^3^	1.1251 × 10^3^
	Std	4.5062 × 10^1^	4.4834 × 10^1^	4.3971 × 10^1^	2.5571 × 10^1^
CEC2017-F12	Ave	2.6469 × 10^4^	2.9180 × 10^4^	2.7267 × 10^4^	2.5468 × 10^3^
	Std	2.0869 × 10^4^	2.0623 × 10^4^	1.5669 × 10^4^	5.6942 × 10^2^
CEC2017-F13	Ave	1.6453 × 10^3^	4.5828 × 10^3^	4.1883 × 10^3^	1.3452 × 10^3^
	Std	2.0936 × 10^2^	1.6759 × 10^3^	1.1327 × 10^3^	3.2223 × 10^1^
CEC2017-F14	Ave	1.4891 × 10^3^	1.5474 × 10^3^	1.5493 × 10^3^	1.4281 × 10^3^
	Std	2.8302 × 10^1^	5.1296 × 10^1^	4.5612 × 10^1^	3.6879 × 10^00^
CEC2017-F15	Ave	1.6953 × 10^3^	1.8583 × 10^3^	1.9588 × 10^3^	1.5176 × 10^3^
	Std	1.2336 × 10^2^	9.7534 × 10^1^	1.9385 × 10^2^	6.9654 × 10^00^
CEC2017-F16	Ave	2.1973 × 10^3^	2.2197 × 10^3^	2.0098 × 10^3^	1.9806 × 10^3^
	Std	2.2161 × 10^2^	2.0916 × 10^2^	1.9007 × 10^2^	2.3374 × 10^2^
CEC2017-F17	Ave	1.8120 × 10^3^	1.8293 × 10^3^	1.8281 × 10^3^	1.7369 × 10^3^
	Std	4.4827 × 10^1^	5.4628 × 10^1^	6.3643 × 10^1^	7.6043 × 10^00^
CEC2017-F18	Ave	2.0552 × 10^3^	7.9281 × 10^3^	6.0787 × 10^3^	1.8657 × 10^3^
	Std	2.1935 × 10^2^	7.1912 × 10^3^	4.3390 × 10^3^	3.4847 × 10^1^
CEC2017-F19	Ave	1.9953 × 10^3^	2.0495 × 10^3^	2.0631 × 10^3^	1.9130 × 10^3^
	Std	5.4975 × 10^1^	8.1484 × 10^1^	6.6187 × 10^1^	3.8464 × 10^00^
CEC2017-F20	Ave	2.2023 × 10^3^	2.2135 × 10^3^	2.1694 × 10^3^	2.0936 × 10^3^
	Std	8.0431 × 10^1^	1.0684 × 10^2^	6.7029 × 10^1^	6.0960 × 10^1^
CEC2017-F21	Ave	2.3295 × 10^3^	2.3346 × 10^3^	2.3381 × 10^3^	2.3185 × 10^3^
	Std	5.6685 × 10^00^	6.5256 × 10^00^	8.2807 × 10^00^	6.7052 × 10^00^
CEC2017-F22	Ave	2.4894 × 10^3^	2.3012 × 10^3^	2.3798 × 10^3^	2.3000 × 10^3^
	Std	7.0965 × 10^2^	1.7589 × 10^00^	4.3251 × 10^2^	1.4626 × 10^−13^
CEC2017-F23	Ave	2.6797 × 10^3^	2.6870 × 10^3^	2.6885 × 10^3^	2.6598 × 10^3^
	Std	6.1832 × 10^00^	8.7358 × 10^00^	9.3977 × 10^00^	9.6610 × 10^00^
CEC2017-F24	Ave	2.8487 × 10^3^	2.8611 × 10^3^	2.8574 × 10^3^	2.8339 × 10^3^
	Std	5.3234 × 10^00^	1.0471 × 10^1^	1.1075 × 10^1^	5.6674 × 10^00^
CEC2017-F25	Ave	2.8886 × 10^3^	2.8914 × 10^3^	2.8893 × 10^3^	2.8869 × 10^3^
	Std	6.8082 × 10^00^	9.1486 × 10^00^	3.8007 × 10^00^	8.2095 × 10^−2^
CEC2017-F26	Ave	3.8914 × 10^3^	3.9787 × 10^3^	3.9573 × 10^3^	3.6409 × 10^3^
	Std	1.2628 × 10^2^	2.4512 × 10^2^	2.5347 × 10^2^	9.3325 × 10^1^
CEC2017-F27	Ave	3.2163 × 10^3^	3.2222 × 10^3^	3.2174 × 10^3^	3.2107 × 10^3^
	Std	9.9239 × 10^00^	9.9340 × 10^00^	1.0903 × 10^1^	7.2401 × 10^00^
CEC2017-F28	Ave	3.1916 × 10^3^	3.1553 × 10^3^	3.2064 × 10^3^	3.1290 × 10^3^
	Std	5.4058 × 10^1^	5.9772 × 10^1^	3.6538 × 10^1^	4.8940 × 10^1^
CEC2017-F29	Ave	3.4567 × 10^3^	3.4442 × 10^3^	3.4773 × 10^3^	3.3715 × 10^3^
	Std	6.0365 × 10^1^	6.8641 × 10^1^	6.8330 × 10^1^	1.9535 × 10^1^
CEC2017-F30	Ave	5.8124 × 10^3^	8.5509 × 10^3^	7.9381 × 10^3^	5.2900 × 10^3^
	Std	6.6711 × 10^2^	3.2951 × 10^3^	2.6561 × 10^3^	1.6663 × 10^2^

**Table 10:** Strategy effectiveness analysis experimental results.

Suites	CEC2014		CEC2017				CEC2020			
Dimensions	30		30		100		10		20	
Algorithms	M.R	T.R	M.R	T.R	M.R	T.R	M.R	T.R	M.R	T.R
SBOA	4.40	5	4.97	5	4.83	5	4.20	5	4.40	5
SBOA1	3.43	4	3.50	4	3.40	4	3.80	4	3.90	4
SBOA2	3.10	3	2.83	3	3.13	3	3.00	2	3.10	3
SBOA3	2.57	2	2.67	2	2.30	2	3.00	2	2.60	2
MISBOA	1.50	1	1.03	1	1.33	1	1.00	1	1.00	1

**Table 11:** Ave and Std of the optimal fitness values with Otsu as the objective function.

Image	Threshold	Metric	BHO	NDO	GJA	SSLO	LEE	SAO	RBMO	ESC	SBOA	MISBOA
baboon	4	Ave	1.008 × 10^3^	1.008 × 10^3^	1.008 × 10^3^	1.008 × 10^3^	1.008 × 10^3^	1.008 × 10^3^	1.008 × 10^3^	1.008 × 10^3^	1.008 × 10^3^	1.008 × 10^3^
		Std	7.357 × 10^−3^	9.781 × 10^−2^	2.313 × 10^−13^	8.290 × 10^−2^	2.313 × 10^−13^	2.313 × 10^−13^	2.313 × 10^−13^	1.873 × 10^−2^	4.823 × 10^−3^	4.823 × 10^−3^
	6	Ave	1.036 × 10^3^	1.002 × 10^3^	1.036 × 10^3^	1.036 × 10^3^	1.036 × 10^3^	1.036 × 10^3^	1.036 × 10^3^	1.036 × 10^3^	1.036 × 10^3^	1.036 × 10^3^
		Std	3.299 × 10^−2^	1.892 × 10^2^	1.600 × 10^−2^	2.586 × 10^−1^	4.625 × 10^−13^	2.194 × 10^−2^	1.836 × 10^−2^	2.540 × 10^−1^	3.795 × 10^−3^	5.532 × 10^−4^
	8	Ave	1.049 × 10^3^	7.690 × 10^2^	1.049 × 10^3^	8.380 × 10^2^	1.049 × 10^3^	1.049 × 10^3^	1.014 × 10^3^	1.048 × 10^3^	1.049 × 10^3^	1.049 × 10^3^
		Std	1.739 × 10^−1^	4.717 × 10^2^	5.449 × 10^−2^	4.262 × 10^2^	3.416 × 10^−3^	5.035 × 10^−2^	1.915 × 10^2^	4.951 × 10^−1^	4.070 × 10^−2^	8.525 × 10^−2^
	10	Ave	1.055 × 10^3^	4.221 × 10^2^	1.055 × 10^3^	5.965 × 10^2^	1.056 × 10^3^	1.020 × 10^3^	8.444 × 10^2^	1.054 × 10^3^	9.851 × 10^2^	1.056 × 10^3^
		Std	2.146 × 10^−1^	5.258 × 10^2^	1.358 × 10^−1^	5.305 × 10^2^	2.799 × 10^−2^	1.927 × 10^2^	4.294 × 10^2^	4.437 × 10^−1^	2.678 × 10^2^	6.553 × 10^−2^
bank	4	Ave	3.602 × 10^3^	3.602 × 10^3^	3.602 × 10^3^	3.602 × 10^3^	3.602 × 10^3^	3.602 × 10^3^	3.602 × 10^3^	3.602 × 10^3^	3.602 × 10^3^	3.602 × 10^3^
		Std	2.776 × 10^−2^	3.824 × 10^−2^	2.957 × 10^−3^	1.088 × 10^−1^	1.850 × 10^−12^	2.183 × 10^−1^	3.139 × 10^−3^	2.454 × 10^−1^	5.688 × 10^−3^	2.957 × 10^−3^
	6	Ave	3.681 × 10^3^	3.681 × 10^3^	3.681 × 10^3^	3.680 × 10^3^	3.681 × 10^3^	3.681 × 10^3^	3.681 × 10^3^	3.680 × 10^3^	3.681 × 10^3^	3.681 × 10^3^
		Std	6.435 × 10^−2^	7.107 × 10^−1^	2.099 × 10^−2^	8.351 × 10^−1^	1.388 × 10^−12^	1.548 × 10^−2^	3.033 × 10^−2^	9.351 × 10^−1^	2.164 × 10^−2^	1.388 × 10^−12^
	8	Ave	3.711 × 10^3^	3.711 × 10^3^	3.712 × 10^3^	3.710 × 10^3^	3.712 × 10^3^	3.711 × 10^3^	3.711 × 10^3^	3.709 × 10^3^	3.711 × 10^3^	3.712 × 10^3^
		Std	2.104 × 10^−1^	1.322 × 10^00^	5.751 × 10^−2^	8.201 × 10^−1^	8.268 × 10^−3^	1.745 × 10^00^	3.917 × 10^−1^	1.204 × 10^00^	4.103 × 10^−1^	2.077 × 10^−1^
	10	Ave	3.727 × 10^3^	3.727 × 10^3^	3.728 × 10^3^	3.725 × 10^3^	3.728 × 10^3^	3.727 × 10^3^	3.727 × 10^3^	3.724 × 10^3^	3.727 × 10^3^	3.728 × 10^3^
		Std	5.755 × 10^−1^	1.304 × 10^00^	1.962 × 10^−1^	1.129 × 10^00^	3.469 × 10^−3^	1.279 × 10^00^	8.652 × 10^−1^	1.088 × 10^00^	9.422 × 10^−1^	3.982 × 10^−1^
barbara	4	Ave	2.645 × 10^3^	2.645 × 10^3^	2.645 × 10^3^	2.645 × 10^3^	2.645 × 10^3^	2.645 × 10^3^	2.645 × 10^3^	2.645 × 10^3^	2.645 × 10^3^	2.645 × 10^3^
		Std	1.850 × 10^−12^	1.464 × 10^−2^	1.850 × 10^−12^	7.256 × 10^−2^	1.850 × 10^−12^	1.850 × 10^−12^	1.850 × 10^−12^	4.736 × 10^−2^	1.850 × 10^−12^	1.850 × 10^−12^
	6	Ave	2.701 × 10^3^	2.701 × 10^3^	2.702 × 10^3^	2.701 × 10^3^	2.702 × 10^3^	2.702 × 10^3^	2.702 × 10^3^	2.701 × 10^3^	2.702 × 10^3^	2.702 × 10^3^
		Std	4.806 × 10^−2^	4.179 × 10^−1^	1.692 × 10^−2^	5.258 × 10^−1^	9.250 × 10^−13^	1.167 × 10^−2^	4.875 × 10^−2^	6.425 × 10^−1^	1.017 × 10^−2^	9.250 × 10^−13^
	8	Ave	2.727 × 10^3^	2.727 × 10^3^	2.727 × 10^3^	2.726 × 10^3^	2.727 × 10^3^	2.727 × 10^3^	2.727 × 10^3^	2.725 × 10^3^	2.727 × 10^3^	2.727 × 10^3^
		Std	9.586 × 10^−2^	3.919 × 10^−1^	3.799 × 10^−2^	5.743 × 10^−1^	4.625 × 10^−13^	2.125 × 10^−2^	3.254 × 10^−1^	7.797 × 10^−1^	5.484 × 10^−2^	2.079 × 10^−2^
	10	Ave	2.739 × 10^3^	2.739 × 10^3^	2.740 × 10^3^	2.738 × 10^3^	2.740 × 10^3^	2.740 × 10^3^	2.739 × 10^3^	2.737 × 10^3^	2.740 × 10^3^	2.740 × 10^3^
		Std	3.057 × 10^−1^	1.140 × 10^00^	2.730 × 10^−1^	6.164 × 10^−1^	1.327 × 10^−2^	2.295 × 10^−1^	4.912 × 10^−1^	8.514 × 10^−1^	4.416 × 10^−1^	9.999 × 10^−2^
boat	4	Ave	1.970 × 10^3^	1.970 × 10^3^	1.970 × 10^3^	1.970 × 10^3^	1.970 × 10^3^	1.970 × 10^3^	1.970 × 10^3^	1.970 × 10^3^	1.970 × 10^3^	1.970 × 10^3^
		Std	2.376 × 10^−3^	2.694 × 10^−2^	0.000 × 10^00^	1.342 × 10^−1^	0.000 × 10^00^	0.000 × 10^00^	0.000 × 10^00^	4.724 × 10^−2^	0.000 × 10^00^	0.000 × 10^00^
	6	Ave	2.025 × 10^3^	2.025 × 10^3^	2.025 × 10^3^	2.024 × 10^3^	2.025 × 10^3^	2.025 × 10^3^	2.025 × 10^3^	2.025 × 10^3^	2.025 × 10^3^	2.025 × 10^3^
		Std	8.649 × 10^−2^	9.811 × 10^−2^	2.050 × 10^−2^	5.452 × 10^−1^	1.156 × 10^−12^	1.837 × 10^−2^	2.599 × 10^−2^	3.903 × 10^−1^	2.405 × 10^−2^	7.595 × 10^−3^
	8	Ave	2.049 × 10^3^	2.048 × 10^3^	2.049 × 10^3^	2.047 × 10^3^	2.049 × 10^3^	2.049 × 10^3^	2.049 × 10^3^	2.047 × 10^3^	2.049 × 10^3^	2.049 × 10^3^
		Std	1.587 × 10^−1^	7.285 × 10^−1^	4.102 × 10^−2^	6.925 × 10^−1^	1.307 × 10^−3^	2.958 × 10^−2^	2.787 × 10^−1^	9.528 × 10^−1^	2.484 × 10^−1^	5.789 × 10^−2^
	10	Ave	2.060 × 10^3^	2.060 × 10^3^	2.061 × 10^3^	2.059 × 10^3^	2.061 × 10^3^	2.061 × 10^3^	2.061 × 10^3^	2.059 × 10^3^	2.061 × 10^3^	2.061 × 10^3^
		Std	3.238 × 10^−1^	6.520 × 10^−1^	1.551 × 10^−1^	5.862 × 10^−1^	1.344 × 10^−2^	1.674 × 10^−1^	2.361 × 10^−1^	7.034 × 10^−1^	2.476 × 10^−1^	1.490 × 10^−1^
brain	4	Ave	3.731 × 10^3^	3.731 × 10^3^	3.731 × 10^3^	3.731 × 10^3^	3.731 × 10^3^	3.731 × 10^3^	3.731 × 10^3^	3.731 × 10^3^	3.731 × 10^3^	3.731 × 10^3^
		Std	2.313 × 10^−12^	5.278 × 10^−2^	2.313 × 10^−12^	3.779 × 10^−2^	2.313 × 10^−12^	2.313 × 10^−12^	2.313 × 10^−12^	4.472 × 10^−2^	2.313 × 10^−12^	2.313 × 10^−12^
	6	Ave	3.752 × 10^3^	3.751 × 10^3^	3.751 × 10^3^	3.751 × 10^3^	3.751 × 10^3^	3.751 × 10^3^	3.751 × 10^3^	3.751 × 10^3^	3.751 × 10^3^	3.751 × 10^3^
		Std	8.481 × 10^−2^	6.276 × 10^−1^	4.957 × 10^−1^	2.890 × 10^−1^	4.166 × 10^−1^	5.986 × 10^−1^	3.631 × 10^−1^	5.265 × 10^−1^	6.086 × 10^−1^	5.403 × 10^−1^
	8	Ave	3.764 × 10^3^	3.763 × 10^3^	3.764 × 10^3^	3.763 × 10^3^	3.764 × 10^3^	3.763 × 10^3^	3.764 × 10^3^	3.763 × 10^3^	3.764 × 10^3^	3.764 × 10^3^
		Std	1.417 × 10^−1^	8.931 × 10^−1^	2.822 × 10^−2^	2.554 × 10^−1^	5.019 × 10^−3^	1.255 × 10^00^	9.029 × 10^−2^	5.291 × 10^−1^	7.783 × 10^−2^	6.148 × 10^−2^
	10	Ave	3.769 × 10^3^	3.769 × 10^3^	3.769 × 10^3^	3.769 × 10^3^	3.769 × 10^3^	3.769 × 10^3^	3.769 × 10^3^	3.768 × 10^3^	3.769 × 10^3^	3.769 × 10^3^
		Std	1.224 × 10^−1^	3.473 × 10^−1^	9.266 × 10^−2^	2.411 × 10^−1^	2.170 × 10^−2^	8.466 × 10^−2^	1.374 × 10^−1^	3.721 × 10^−1^	1.247 × 10^−1^	2.270 × 10^−1^
bridge	4	Ave	2.640 × 10^3^	2.640 × 10^3^	2.640 × 10^3^	2.640 × 10^3^	2.640 × 10^3^	2.640 × 10^3^	2.640 × 10^3^	2.640 × 10^3^	2.640 × 10^3^	2.640 × 10^3^
		Std	6.308 × 10^−3^	3.850 × 10^−2^	1.388 × 10^−12^	7.806 × 10^−2^	1.388 × 10^−12^	1.388 × 10^−12^	1.388 × 10^−12^	1.691 × 10^−1^	1.388 × 10^−12^	1.388 × 10^−12^
	6	Ave	2.719 × 10^3^	2.718 × 10^3^	2.719 × 10^3^	2.718 × 10^3^	2.719 × 10^3^	2.719 × 10^3^	2.719 × 10^3^	2.718 × 10^3^	2.719 × 10^3^	2.719 × 10^3^
		Std	7.795 × 10^−2^	5.065 × 10^−1^	1.242 × 10^−2^	7.046 × 10^−1^	2.313 × 10^−12^	2.204 × 10^−2^	7.592 × 10^−2^	8.565 × 10^−1^	1.690 × 10^−2^	1.074 × 10^−2^
	8	Ave	2.752 × 10^3^	2.751 × 10^3^	2.752 × 10^3^	2.750 × 10^3^	2.752 × 10^3^	2.752 × 10^3^	2.752 × 10^3^	2.750 × 10^3^	2.752 × 10^3^	2.752 × 10^3^
		Std	2.085 × 10^−1^	9.252 × 10^−1^	4.128 × 10^−2^	1.026 × 10^00^	5.943 × 10^−3^	2.048 × 10^−2^	3.281 × 10^−1^	1.165 × 10^00^	1.005 × 10^−1^	2.886 × 10^−2^
	10	Ave	2.768 × 10^3^	2.767 × 10^3^	2.768 × 10^3^	2.766 × 10^3^	2.768 × 10^3^	2.768 × 10^3^	2.768 × 10^3^	2.766 × 10^3^	2.768 × 10^3^	2.768 × 10^3^
		Std	4.287 × 10^−1^	1.534 × 10^00^	1.480 × 10^−1^	7.036 × 10^−1^	2.744 × 10^−2^	6.493 × 10^−1^	3.813 × 10^−1^	8.860 × 10^−1^	2.553 × 10^−1^	6.314 × 10^−1^
camera	4	Ave	4.601 × 10^3^	4.600 × 10^3^	4.600 × 10^3^	4.601 × 10^3^	4.600 × 10^3^	4.599 × 10^3^	4.601 × 10^3^	4.601 × 10^3^	4.601 × 10^3^	4.601 × 10^3^
		Std	6.121 × 10^−1^	1.125 × 10^00^	1.018 × 10^00^	3.118 × 10^−1^	9.838 × 10^−1^	1.267 × 10^00^	6.774 × 10^−3^	5.419 × 10^−1^	8.166 × 10^−1^	1.116 × 10^−2^
	6	Ave	4.652 × 10^3^	4.652 × 10^3^	4.652 × 10^3^	4.651 × 10^3^	4.652 × 10^3^	4.652 × 10^3^	4.652 × 10^3^	4.651 × 10^3^	4.652 × 10^3^	4.652 × 10^3^
		Std	6.146 × 10^−2^	3.386 × 10^−1^	8.109 × 10^−3^	4.799 × 10^−1^	9.250 × 10^−13^	3.344 × 10^−3^	6.793 × 10^−2^	3.450 × 10^−1^	3.532 × 10^−3^	7.707 × 10^−3^
	8	Ave	4.670 × 10^3^	4.670 × 10^3^	4.669 × 10^3^	4.669 × 10^3^	4.669 × 10^3^	4.670 × 10^3^	4.670 × 10^3^	4.669 × 10^3^	4.670 × 10^3^	4.670 × 10^3^
		Std	5.763 × 10^−1^	9.557 × 10^−1^	6.473 × 10^−1^	7.773 × 10^−1^	6.870 × 10^−1^	7.990 × 10^−1^	8.342 × 10^−1^	6.971 × 10^−1^	8.696 × 10^−1^	8.593 × 10^−1^
	10	Ave	4.681 × 10^3^	4.680 × 10^3^	4.681 × 10^3^	4.679 × 10^3^	4.681 × 10^3^	4.681 × 10^3^	4.681 × 10^3^	4.679 × 10^3^	4.681 × 10^3^	4.681 × 10^3^
		Std	1.987 × 10^−1^	1.246 × 10^00^	1.085 × 10^−1^	6.064 × 10^−1^	4.589 × 10^−2^	4.651 × 10^−2^	4.228 × 10^−1^	7.403 × 10^−1^	1.043 × 10^−1^	3.906 × 10^−1^
cell	4	Ave	2.567 × 10^3^	2.567 × 10^3^	2.567 × 10^3^	2.567 × 10^3^	2.567 × 10^3^	2.567 × 10^3^	2.567 × 10^3^	2.567 × 10^3^	2.567 × 10^3^	2.567 × 10^3^
		Std	5.157 × 10^−3^	9.250 × 10^−13^	9.250 × 10^−13^	4.078 × 10^−2^	9.250 × 10^−13^	9.250 × 10^−13^	9.250 × 10^−13^	3.711 × 10^−3^	9.250 × 10^−13^	9.250 × 10^−13^
	6	Ave	2.596 × 10^3^	2.596 × 10^3^	2.596 × 10^3^	2.596 × 10^3^	2.596 × 10^3^	2.596 × 10^3^	2.596 × 10^3^	2.596 × 10^3^	2.596 × 10^3^	2.596 × 10^3^
		Std	9.540 × 10^−2^	2.254 × 10^−1^	1.417 × 10^−1^	2.079 × 10^−1^	1.338 × 10^−1^	1.443 × 10^−1^	1.484 × 10^−1^	1.685 × 10^−1^	1.166 × 10^−1^	8.480 × 10^−2^
	8	Ave	2.612 × 10^3^	2.612 × 10^3^	2.612 × 10^3^	2.611 × 10^3^	2.612 × 10^3^	2.612 × 10^3^	2.612 × 10^3^	2.611 × 10^3^	2.612 × 10^3^	2.612 × 10^3^
		Std	1.108 × 10^−1^	4.209 × 10^−1^	1.781 × 10^−1^	3.816 × 10^−1^	1.511 × 10^−3^	5.411 × 10^−2^	9.064 × 10^−2^	3.157 × 10^−1^	1.087 × 10^−1^	4.626 × 10^−2^
	10	Ave	2.619 × 10^3^	2.619 × 10^3^	2.619 × 10^3^	2.618 × 10^3^	2.620 × 10^3^	2.619 × 10^3^	2.619 × 10^3^	2.618 × 10^3^	2.620 × 10^3^	2.620 × 10^3^
		Std	2.674 × 10^−1^	5.644 × 10^−1^	3.128 × 10^−1^	5.584 × 10^−1^	4.021 × 10^−3^	6.670 × 10^−1^	4.164 × 10^−1^	3.553 × 10^−1^	3.119 × 10^−1^	3.201 × 10^−1^
columbia	4	Ave	1.827 × 10^3^	1.827 × 10^3^	1.827 × 10^3^	1.827 × 10^3^	1.827 × 10^3^	1.827 × 10^3^	1.827 × 10^3^	1.827 × 10^3^	1.827 × 10^3^	1.827 × 10^3^
		Std	9.250 × 10^−13^	2.239 × 10^−2^	9.250 × 10^−13^	7.610 × 10^−2^	9.250 × 10^−13^	1.075 × 10^−3^	7.197 × 10^−4^	3.142 × 10^−2^	9.250 × 10^−13^	9.250 × 10^−13^
	6	Ave	1.878 × 10^3^	1.878 × 10^3^	1.878 × 10^3^	1.877 × 10^3^	1.878 × 10^3^	1.878 × 10^3^	1.878 × 10^3^	1.878 × 10^3^	1.878 × 10^3^	1.878 × 10^3^
		Std	2.831 × 10^−2^	3.070 × 10^−1^	1.985 × 10^−2^	2.857 × 10^−1^	6.938 × 10^−13^	2.776 × 10^−2^	4.494 × 10^−2^	5.555 × 10^−1^	3.277 × 10^−2^	2.655 × 10^−3^
	8	Ave	1.899 × 10^3^	1.899 × 10^3^	1.899 × 10^3^	1.898 × 10^3^	1.899 × 10^3^	1.899 × 10^3^	1.899 × 10^3^	1.898 × 10^3^	1.899 × 10^3^	1.899 × 10^3^
		Std	9.978 × 10^−2^	8.472 × 10^−1^	6.044 × 10^−2^	5.039 × 10^−1^	9.072 × 10^−3^	3.866 × 10^−2^	1.108 × 10^−1^	6.579 × 10^−1^	4.299 × 10^−2^	2.129 × 10^−2^
	10	Ave	1.910 × 10^3^	1.910 × 10^3^	1.910 × 10^3^	1.908 × 10^3^	1.910 × 10^3^	1.910 × 10^3^	1.910 × 10^3^	1.908 × 10^3^	1.910 × 10^3^	1.910 × 10^3^
		Std	2.226 × 10^−1^	6.125 × 10^−1^	1.688 × 10^−1^	6.336 × 10^−1^	4.588 × 10^−3^	1.607 × 10^−1^	2.833 × 10^−1^	6.890 × 10^−1^	1.852 × 10^−1^	4.951 × 10^−2^
couple	4	Ave	1.733 × 10^3^	1.733 × 10^3^	1.733 × 10^3^	1.733 × 10^3^	1.733 × 10^3^	1.733 × 10^3^	1.733 × 10^3^	1.733 × 10^3^	1.733 × 10^3^	1.733 × 10^3^
		Std	2.928 × 10^−2^	1.957 × 10^−1^	1.156 × 10^−12^	9.090 × 10^−2^	1.156 × 10^−12^	1.588 × 10^−2^	9.365 × 10^−3^	1.592 × 10^−1^	1.588 × 10^−2^	1.156 × 10^−12^
	6	Ave	1.799 × 10^3^	1.799 × 10^3^	1.799 × 10^3^	1.798 × 10^3^	1.799 × 10^3^	1.799 × 10^3^	1.799 × 10^3^	1.798 × 10^3^	1.799 × 10^3^	1.799 × 10^3^
		Std	8.390 × 10^−2^	9.501 × 10^−2^	2.431 × 10^−2^	6.145 × 10^−1^	2.367 × 10^−3^	1.792 × 10^−2^	3.931 × 10^−2^	7.056 × 10^−1^	1.401 × 10^−2^	2.367 × 10^−3^
	8	Ave	1.826 × 10^3^	1.826 × 10^3^	1.826 × 10^3^	1.825 × 10^3^	1.826 × 10^3^	1.826 × 10^3^	1.826 × 10^3^	1.825 × 10^3^	1.826 × 10^3^	1.826 × 10^3^
		Std	1.840 × 10^−1^	7.990 × 10^−1^	5.759 × 10^−2^	8.329 × 10^−1^	0.000 × 10^00^	1.800 × 10^−2^	1.184 × 10^−1^	9.247 × 10^−1^	1.309 × 10^−1^	2.180 × 10^−2^
	10	Ave	1.839 × 10^3^	1.839 × 10^3^	1.840 × 10^3^	1.838 × 10^3^	1.840 × 10^3^	1.840 × 10^3^	1.839 × 10^3^	1.837 × 10^3^	1.839 × 10^3^	1.840 × 10^3^
		Std	4.315 × 10^−1^	1.314 × 10^00^	1.323 × 10^−1^	6.939 × 10^−1^	7.430 × 10^−3^	2.443 × 10^−1^	3.022 × 10^−1^	9.746 × 10^−1^	5.120 × 10^−1^	1.463 × 10^−1^

**Table 12:** Ave and Std of all test images for PSNR in Otsu.

Image	Threshold	Metric	BHO	NDO	GJA	SSLO	LEE	SAO	RBMO	ESC	SBOA	MISBOA
baboon	4	Ave	2.0043 × 10^1^	2.0013 × 10^1^	2.0043 × 10^1^	1.9984 × 10^1^	2.0043 × 10^1^	2.0043 × 10^1^	2.0043 × 10^1^	2.0027 × 10^1^	2.0038 × 10^1^	2.0038 × 10^1^
		Std	1.8220 × 10^−3^	9.3751 × 10^−2^	7.2269 × 10^−15^	1.5140 × 10^−1^	7.2269 × 10^−15^	7.2269 × 10^−15^	7.2269 × 10^−15^	4.9761 × 10^−2^	2.8753 × 10^−2^	2.8753 × 10^−2^
	6	Ave	2.2783 × 10^1^	2.2199 × 10^1^	2.2788 × 10^1^	2.2706 × 10^1^	2.2732 × 10^1^	2.2729 × 10^1^	2.2733 × 10^1^	2.2634 × 10^1^	2.2772 × 10^1^	2.2747 × 10^1^
		Std	1.6022 × 10^−1^	3.1269 × 10^00^	1.3727 × 10^−1^	4.5581 × 10^−1^	7.2269 × 10^−15^	7.9669 × 10^−2^	5.2945 × 10^−2^	4.4727 × 10^−1^	1.1033 × 10^−1^	5.6263 × 10^−2^
	8	Ave	2.5170 × 10^1^	1.9878 × 10^1^	2.5180 × 10^1^	2.1069 × 10^1^	2.5346 × 10^1^	2.5187 × 10^1^	2.4900 × 10^1^	2.4725 × 10^1^	2.5248 × 10^1^	2.5180 × 10^1^
		Std	4.9283 × 10^−1^	8.6792 × 10^00^	3.0325 × 10^−1^	7.7697 × 10^00^	4.0809 × 10^−2^	3.5620 × 10^−1^	1.0685 × 10^00^	7.3364 × 10^−1^	3.7311 × 10^−1^	3.9277 × 10^−1^
	10	Ave	2.6875 × 10^1^	1.4206 × 10^1^	2.6782 × 10^1^	1.7943 × 10^1^	2.6825 × 10^1^	2.6506 × 10^1^	2.5146 × 10^1^	2.6034 × 10^1^	2.6305 × 10^1^	2.6810 × 10^1^
		Std	5.0870 × 10^−1^	1.0532 × 10^1^	3.5161 × 10^−1^	1.0714 × 10^1^	1.0775 × 10^−1^	1.8776 × 10^00^	3.8589 × 10^00^	8.3462 × 10^−1^	3.4924 × 10^00^	3.0576 × 10^−1^
bank	4	Ave	2.0250 × 10^1^	2.0247 × 10^1^	2.0253 × 10^1^	2.0234 × 10^1^	2.0254 × 10^1^	2.0245 × 10^1^	2.0253 × 10^1^	2.0236 × 10^1^	2.0252 × 10^1^	2.0253 × 10^1^
		Std	1.2877 × 10^−2^	1.3920 × 10^−2^	2.3359 × 10^−3^	1.9698 × 10^−2^	1.4454 × 10^−14^	2.7991 × 10^−2^	3.4256 × 10^−3^	2.9514 × 10^−2^	5.0318 × 10^−3^	2.3359 × 10^−3^
	6	Ave	2.3098 × 10^1^	2.3087 × 10^1^	2.3105 × 10^1^	2.3003 × 10^1^	2.3097 × 10^1^	2.3101 × 10^1^	2.3100 × 10^1^	2.3082 × 10^1^	2.3101 × 10^1^	2.3097 × 10^1^
		Std	1.2198 × 10^−2^	4.7784 × 10^−2^	6.9615 × 10^−3^	7.7247 × 10^−2^	3.6134 × 10^−15^	5.6647 × 10^−3^	4.7340 × 10^−3^	6.0110 × 10^−2^	1.1827 × 10^−2^	3.6134 × 10^−15^
	8	Ave	2.5276 × 10^1^	2.5212 × 10^1^	2.5284 × 10^1^	2.5094 × 10^1^	2.5293 × 10^1^	2.5258 × 10^1^	2.5274 × 10^1^	2.5107 × 10^1^	2.5282 × 10^1^	2.5296 × 10^1^
		Std	4.6213 × 10^−2^	1.3222 × 10^−1^	1.7980 × 10^−2^	1.0749 × 10^−1^	1.3611 × 10^−2^	1.6128 × 10^−1^	3.6399 × 10^−2^	1.3672 × 10^−1^	4.1472 × 10^−2^	1.8264 × 10^−2^
	10	Ave	2.6852 × 10^1^	2.6815 × 10^1^	2.6918 × 10^1^	2.6607 × 10^1^	2.6931 × 10^1^	2.6884 × 10^1^	2.6873 × 10^1^	2.6588 × 10^1^	2.6890 × 10^1^	2.6909 × 10^1^
		Std	8.3915 × 10^−2^	1.5534 × 10^−1^	3.8050 × 10^−2^	1.3514 × 10^−1^	8.2387 × 10^−3^	1.5275 × 10^−1^	9.3005 × 10^−2^	1.4243 × 10^−1^	1.0596 × 10^−1^	7.9087 × 10^−2^
barbara	4	Ave	1.8769 × 10^1^	1.8769 × 10^1^	1.8769 × 10^1^	1.8772 × 10^1^	1.8769 × 10^1^	1.8769 × 10^1^	1.8769 × 10^1^	1.8769 × 10^1^	1.8769 × 10^1^	1.8769 × 10^1^
		Std	7.2269 × 10^−15^	2.3013 × 10^−4^	7.2269 × 10^−15^	1.9208 × 10^−2^	7.2269 × 10^−15^	7.2269 × 10^−15^	7.2269 × 10^−15^	1.9589 × 10^−2^	7.2269 × 10^−15^	7.2269 × 10^−15^
	6	Ave	2.1076 × 10^1^	2.1117 × 10^1^	2.1067 × 10^1^	2.1064 × 10^1^	2.1054 × 10^1^	2.1056 × 10^1^	2.1072 × 10^1^	2.1061 × 10^1^	2.1063 × 10^1^	2.1054 × 10^1^
		Std	3.7126 × 10^−2^	9.0396 × 10^−2^	2.7941 × 10^−2^	1.6756 × 10^−1^	0.0000 × 10^00^	1.1628 × 10^−2^	3.3870 × 10^−2^	1.3449 × 10^−1^	2.2699 × 10^−2^	0.0000 × 10^00^
	8	Ave	2.3107 × 10^1^	2.3161 × 10^1^	2.3113 × 10^1^	2.2993 × 10^1^	2.3183 × 10^1^	2.3149 × 10^1^	2.3175 × 10^1^	2.2827 × 10^1^	2.3111 × 10^1^	2.3163 × 10^1^
		Std	1.5207 × 10^−1^	2.5714 × 10^−1^	1.0024 × 10^−1^	3.5611 × 10^−1^	1.0840 × 10^−14^	6.1531 × 10^−2^	1.2344 × 10^−1^	3.1101 × 10^−1^	1.1358 × 10^−1^	5.8799 × 10^−2^
	10	Ave	2.4595 × 10^1^	2.4585 × 10^1^	2.4634 × 10^1^	2.4525 × 10^1^	2.4636 × 10^1^	2.4631 × 10^1^	2.4600 × 10^1^	2.4207 × 10^1^	2.4550 × 10^1^	2.4641 × 10^1^
		Std	2.0911 × 10^−1^	2.3445 × 10^−1^	1.7159 × 10^−1^	3.6423 × 10^−1^	5.2702 × 10^−2^	1.1395 × 10^−1^	2.0340 × 10^−1^	5.6873 × 10^−1^	2.8118 × 10^−1^	8.2002 × 10^−2^
boat	4	Ave	1.9897 × 10^1^	1.9901 × 10^1^	1.9898 × 10^1^	1.9892 × 10^1^	1.9898 × 10^1^	1.9898 × 10^1^	1.9898 × 10^1^	1.9898 × 10^1^	1.9898 × 10^1^	1.9898 × 10^1^
		Std	1.2417 × 10^−3^	1.2035 × 10^−2^	1.0840 × 10^−14^	4.7619 × 10^−2^	1.0840 × 10^−14^	1.0840 × 10^−14^	1.0840 × 10^−14^	1.9761 × 10^−2^	1.0840 × 10^−14^	1.0840 × 10^−14^
	6	Ave	2.3032 × 10^1^	2.3036 × 10^1^	2.3045 × 10^1^	2.2965 × 10^1^	2.3062 × 10^1^	2.3039 × 10^1^	2.3042 × 10^1^	2.2971 × 10^1^	2.3032 × 10^1^	2.3058 × 10^1^
		Std	5.2951 × 10^−2^	5.1484 × 10^−2^	2.5596 × 10^−2^	1.4360 × 10^−1^	1.0840 × 10^−14^	3.5706 × 10^−2^	4.0395 × 10^−2^	8.7513 × 10^−2^	3.2469 × 10^−2^	1.0742 × 10^−2^
	8	Ave	2.5054 × 10^1^	2.5088 × 10^1^	2.5086 × 10^1^	2.5125 × 10^1^	2.5128 × 10^1^	2.5115 × 10^1^	2.5137 × 10^1^	2.4897 × 10^1^	2.5107 × 10^1^	2.5093 × 10^1^
		Std	1.2334 × 10^−1^	1.7846 × 10^−1^	7.1810 × 10^−2^	2.9238 × 10^−1^	5.0066 × 10^−2^	5.9528 × 10^−2^	9.6351 × 10^−2^	3.1784 × 10^−1^	1.2537 × 10^−1^	6.6630 × 10^−2^
	10	Ave	2.6904 × 10^1^	2.6848 × 10^1^	2.6872 × 10^1^	2.6734 × 10^1^	2.6992 × 10^1^	2.7013 × 10^1^	2.6895 × 10^1^	2.6471 × 10^1^	2.6783 × 10^1^	2.6883 × 10^1^
		Std	2.7577 × 10^−1^	3.3114 × 10^−1^	2.1533 × 10^−1^	3.5248 × 10^−1^	2.8711 × 10^−2^	1.5284 × 10^−1^	2.1437 × 10^−1^	3.7559 × 10^−1^	2.9500 × 10^−1^	1.9512 × 10^−1^
brain	4	Ave	2.5074 × 10^1^	2.5074 × 10^1^	2.5080 × 10^1^	2.5067 × 10^1^	2.5066 × 10^1^	2.5072 × 10^1^	2.5056 × 10^1^	2.5079 × 10^1^	2.5060 × 10^1^	2.5084 × 10^1^
		Std	5.2921 × 10^−2^	5.4889 × 10^−2^	5.5675 × 10^−2^	7.4146 × 10^−2^	4.8494 × 10^−2^	5.8925 × 10^−2^	4.7507 × 10^−2^	4.8104 × 10^−2^	5.2566 × 10^−2^	4.7593 × 10^−2^
	6	Ave	2.7315 × 10^1^	2.7510 × 10^1^	2.7572 × 10^1^	2.7195 × 10^1^	2.7556 × 10^1^	2.7474 × 10^1^	2.7324 × 10^1^	2.7329 × 10^1^	2.7489 × 10^1^	2.7410 × 10^1^
		Std	9.9469 × 10^−2^	2.2300 × 10^−1^	1.2711 × 10^−1^	1.4883 × 10^−1^	9.7655 × 10^−2^	1.9204 × 10^−1^	1.1499 × 10^−1^	2.4033 × 10^−1^	1.6065 × 10^−1^	1.3366 × 10^−1^
	8	Ave	2.9518 × 10^1^	2.9438 × 10^1^	2.9532 × 10^1^	2.9290 × 10^1^	2.9554 × 10^1^	2.9494 × 10^1^	2.9509 × 10^1^	2.9309 × 10^1^	2.9534 × 10^1^	2.9555 × 10^1^
		Std	1.0328 × 10^−1^	1.7318 × 10^−1^	7.7984 × 10^−2^	1.8247 × 10^−1^	5.8882 × 10^−2^	2.0732 × 10^−1^	9.2775 × 10^−2^	1.8462 × 10^−1^	6.3379 × 10^−2^	6.2975 × 10^−2^
	10	Ave	3.0948 × 10^1^	3.0910 × 10^1^	3.1031 × 10^1^	3.0692 × 10^1^	3.1096 × 10^1^	3.0978 × 10^1^	3.0940 × 10^1^	3.0669 × 10^1^	3.0980 × 10^1^	3.0980 × 10^1^
		Std	1.3441 × 10^−1^	1.8819 × 10^−1^	1.3002 × 10^−1^	2.2361 × 10^−1^	8.2212 × 10^−2^	1.6043 × 10^−1^	1.7196 × 10^−1^	2.2679 × 10^−1^	1.9969 × 10^−1^	2.0238 × 10^−1^
bridge	4	Ave	1.8547 × 10^1^	1.8563 × 10^1^	1.8546 × 10^1^	1.8562 × 10^1^	1.8546 × 10^1^	1.8546 × 10^1^	1.8546 × 10^1^	1.8551 × 10^1^	1.8546 × 10^1^	1.8546 × 10^1^
		Std	1.4255 × 10^−3^	4.4020 × 10^−2^	3.6134 × 10^−15^	6.0258 × 10^−2^	3.6134 × 10^−15^	3.6134 × 10^−15^	3.6134 × 10^−15^	6.4280 × 10^−2^	3.6134 × 10^−15^	3.6134 × 10^−15^
	6	Ave	2.1658 × 10^1^	2.1672 × 10^1^	2.1668 × 10^1^	2.1654 × 10^1^	2.1659 × 10^1^	2.1648 × 10^1^	2.1650 × 10^1^	2.1584 × 10^1^	2.1663 × 10^1^	2.1657 × 10^1^
		Std	7.2423 × 10^−2^	1.2255 × 10^−1^	2.8597 × 10^−2^	1.9936 × 10^−1^	0.0000 × 10^00^	2.2355 × 10^−2^	4.6133 × 10^−2^	2.3117 × 10^−1^	2.6904 × 10^−2^	2.0880 × 10^−2^
	8	Ave	2.3981 × 10^1^	2.3866 × 10^1^	2.4011 × 10^1^	2.3821 × 10^1^	2.4015 × 10^1^	2.4009 × 10^1^	2.3965 × 10^1^	2.3788 × 10^1^	2.3989 × 10^1^	2.4014 × 10^1^
		Std	1.3956 × 10^−1^	2.1980 × 10^−1^	6.8789 × 10^−2^	3.5727 × 10^−1^	3.2111 × 10^−2^	5.6899 × 10^−2^	1.4357 × 10^−1^	3.3445 × 10^−1^	1.2304 × 10^−1^	6.5003 × 10^−2^
	10	Ave	2.5767 × 10^1^	2.5634 × 10^1^	2.5807 × 10^1^	2.5643 × 10^1^	2.5909 × 10^1^	2.5869 × 10^1^	2.5805 × 10^1^	2.5462 × 10^1^	2.5799 × 10^1^	2.5890 × 10^1^
		Std	2.1725 × 10^−1^	3.4655 × 10^−1^	1.5148 × 10^−1^	2.3667 × 10^−1^	8.8194 × 10^−2^	3.1488 × 10^−1^	2.2674 × 10^−1^	3.3442 × 10^−1^	2.4669 × 10^−1^	2.2270 × 10^−1^
camera	4	Ave	1.9670 × 10^1^	1.9059 × 10^1^	1.8903 × 10^1^	1.9831 × 10^1^	1.9205 × 10^1^	1.8351 × 10^1^	1.9871 × 10^1^	1.9704 × 10^1^	1.9508 × 10^1^	1.9863 × 10^1^
		Std	5.4887 × 10^−1^	9.4311 × 10^−1^	9.2168 × 10^−1^	1.0457 × 10^−1^	8.9029 × 10^−1^	8.8888 × 10^−1^	1.7329 × 10^−3^	3.4369 × 10^−1^	7.3899 × 10^−1^	3.4447 × 10^−2^
	6	Ave	2.1879 × 10^1^	2.1887 × 10^1^	2.1895 × 10^1^	2.1801 × 10^1^	2.1935 × 10^1^	2.1922 × 10^1^	2.1913 × 10^1^	2.1823 × 10^1^	2.1905 × 10^1^	2.1914 × 10^1^
		Std	6.4135 × 10^−2^	1.0619 × 10^−1^	5.1106 × 10^−2^	2.5455 × 10^−1^	3.6134 × 10^−15^	2.7301 × 10^−2^	3.4446 × 10^−2^	1.4054 × 10^−1^	4.2733 × 10^−2^	4.1597 × 10^−2^
	8	Ave	2.3022 × 10^1^	2.3144 × 10^1^	2.3276 × 10^1^	2.2985 × 10^1^	2.3295 × 10^1^	2.3229 × 10^1^	2.3182 × 10^1^	2.2970 × 10^1^	2.3227 × 10^1^	2.3196 × 10^1^
		Std	1.7916 × 10^−1^	1.6877 × 10^−1^	1.6904 × 10^−1^	3.0685 × 10^−1^	1.5584 × 10^−1^	1.7319 × 10^−1^	2.1028 × 10^−1^	3.5030 × 10^−1^	2.3951 × 10^−1^	2.0685 × 10^−1^
	10	Ave	2.4374 × 10^1^	2.4273 × 10^1^	2.4301 × 10^1^	2.4349 × 10^1^	2.4395 × 10^1^	2.4296 × 10^1^	2.4285 × 10^1^	2.3961 × 10^1^	2.4330 × 10^1^	2.4300 × 10^1^
		Std	2.8828 × 10^−1^	3.9483 × 10^−1^	2.8288 × 10^−1^	4.4959 × 10^−1^	2.1134 × 10^−1^	2.2902 × 10^−1^	2.8784 × 10^−1^	4.4186 × 10^−1^	3.2961 × 10^−1^	2.9287 × 10^−1^
cell	4	Ave	1.8639 × 10^1^	1.8639 × 10^1^	1.8639 × 10^1^	1.8654 × 10^1^	1.8639 × 10^1^	1.8639 × 10^1^	1.8639 × 10^1^	1.8639 × 10^1^	1.8639 × 10^1^	1.8639 × 10^1^
		Std	2.1159 × 10^−4^	1.0840 × 10^−14^	1.0840 × 10^−14^	4.4564 × 10^−2^	1.0840 × 10^−14^	1.0840 × 10^−14^	1.0840 × 10^−14^	1.5226 × 10^−4^	1.0840 × 10^−14^	1.0840 × 10^−14^
	6	Ave	1.9899 × 10^1^	1.9898 × 10^1^	1.9869 × 10^1^	1.9956 × 10^1^	1.9940 × 10^1^	1.9834 × 10^1^	1.9827 × 10^1^	1.9806 × 10^1^	1.9838 × 10^1^	1.9879 × 10^1^
		Std	2.0047 × 10^−1^	2.2446 × 10^−1^	2.1960 × 10^−1^	2.0985 × 10^−1^	1.9591 × 10^−1^	2.3455 × 10^−1^	2.4327 × 10^−1^	2.2655 × 10^−1^	2.5270 × 10^−1^	2.4851 × 10^−1^
	8	Ave	2.0723 × 10^1^	2.0730 × 10^1^	2.0757 × 10^1^	2.0716 × 10^1^	2.0716 × 10^1^	2.0713 × 10^1^	2.0727 × 10^1^	2.0656 × 10^1^	2.0749 × 10^1^	2.0755 × 10^1^
		Std	7.8055 × 10^−2^	1.1628 × 10^−1^	6.2506 × 10^−2^	2.3728 × 10^−1^	2.7727 × 10^−2^	5.9719 × 10^−2^	7.0103 × 10^−2^	1.5750 × 10^−1^	5.7536 × 10^−2^	3.6102 × 10^−2^
	10	Ave	2.1243 × 10^1^	2.1380 × 10^1^	2.1320 × 10^1^	2.1322 × 10^1^	2.1377 × 10^1^	2.1304 × 10^1^	2.1265 × 10^1^	2.1153 × 10^1^	2.1311 × 10^1^	2.1303 × 10^1^
		Std	1.2296 × 10^−1^	2.1800 × 10^−1^	1.1314 × 10^−1^	3.1032 × 10^−1^	1.2424 × 10^−3^	1.2084 × 10^−1^	1.2562 × 10^−1^	2.2105 × 10^−1^	1.2946 × 10^−1^	1.0266 × 10^−1^
columbia	4	Ave	1.9999 × 10^1^	1.9999 × 10^1^	1.9999 × 10^1^	1.9993 × 10^1^	1.9999 × 10^1^	1.9999 × 10^1^	1.9999 × 10^1^	1.9988 × 10^1^	1.9999 × 10^1^	1.9999 × 10^1^
		Std	1.4454 × 10^−14^	4.2826 × 10^−2^	1.4454 × 10^−14^	6.5606 × 10^−2^	1.4454 × 10^−14^	2.6687 × 10^−4^	1.7862 × 10^−4^	4.2424 × 10^−2^	1.4454 × 10^−14^	1.4454 × 10^−14^
	6	Ave	2.2301 × 10^1^	2.2310 × 10^1^	2.2314 × 10^1^	2.2384 × 10^1^	2.2320 × 10^1^	2.2345 × 10^1^	2.2323 × 10^1^	2.2198 × 10^1^	2.2321 × 10^1^	2.2321 × 10^1^
		Std	5.8477 × 10^−2^	1.5826 × 10^−1^	4.3031 × 10^−2^	2.8971 × 10^−1^	7.2269 × 10^−15^	5.9940 × 10^−2^	7.2414 × 10^−2^	1.7924 × 10^−1^	8.1013 × 10^−2^	2.6730 × 10^−3^
	8	Ave	2.4073 × 10^1^	2.3964 × 10^1^	2.4067 × 10^1^	2.3952 × 10^1^	2.4074 × 10^1^	2.4036 × 10^1^	2.4036 × 10^1^	2.3935 × 10^1^	2.4044 × 10^1^	2.4080 × 10^1^
		Std	1.2835 × 10^−1^	2.9780 × 10^−1^	7.7280 × 10^−2^	3.7273 × 10^−1^	3.6052 × 10^−2^	9.6682 × 10^−2^	1.2093 × 10^−1^	2.8412 × 10^−1^	1.1075 × 10^−1^	3.5891 × 10^−2^
	10	Ave	2.5385 × 10^1^	2.5300 × 10^1^	2.5337 × 10^1^	2.5521 × 10^1^	2.5261 × 10^1^	2.5365 × 10^1^	2.5266 × 10^1^	2.4936 × 10^1^	2.5310 × 10^1^	2.5371 × 10^1^
		Std	2.7417 × 10^−1^	3.2611 × 10^−1^	2.4305 × 10^−1^	6.2033 × 10^−1^	7.7994 × 10^−2^	2.6089 × 10^−1^	2.4412 × 10^−1^	3.6354 × 10^−1^	2.9047 × 10^−1^	1.8206 × 10^−1^
couple	4	Ave	2.0264 × 10^1^	2.0246 × 10^1^	2.0269 × 10^1^	2.0247 × 10^1^	2.0269 × 10^1^	2.0255 × 10^1^	2.0265 × 10^1^	2.0242 × 10^1^	2.0255 × 10^1^	2.0269 × 10^1^
		Std	2.1817 × 10^−2^	4.7972 × 10^−2^	7.2269 × 10^−15^	5.5179 × 10^−2^	7.2269 × 10^−15^	2.6489 × 10^−2^	1.5622 × 10^−2^	7.1147 × 10^−2^	2.6489 × 10^−2^	7.2269 × 10^−15^
	6	Ave	2.3446 × 10^1^	2.3454 × 10^1^	2.3460 × 10^1^	2.3379 × 10^1^	2.3460 × 10^1^	2.3465 × 10^1^	2.3462 × 10^1^	2.3391 × 10^1^	2.3462 × 10^1^	2.3460 × 10^1^
		Std	2.5052 × 10^−2^	2.1507 × 10^−2^	1.2529 × 10^−2^	8.0557 × 10^−2^	3.2024 × 10^−3^	1.0898 × 10^−2^	1.3520 × 10^−2^	8.0754 × 10^−2^	8.4720 × 10^−3^	3.2024 × 10^−3^
	8	Ave	2.5361 × 10^1^	2.5366 × 10^1^	2.5391 × 10^1^	2.5195 × 10^1^	2.5426 × 10^1^	2.5421 × 10^1^	2.5406 × 10^1^	2.5271 × 10^1^	2.5412 × 10^1^	2.5420 × 10^1^
		Std	4.5081 × 10^−2^	1.2493 × 10^−1^	2.1050 × 10^−2^	1.5715 × 10^−1^	3.6134 × 10^−15^	1.2129 × 10^−2^	2.6196 × 10^−2^	1.1442 × 10^−1^	1.6682 × 10^−2^	9.8911 × 10^−3^
	10	Ave	2.6788 × 10^1^	2.6788 × 10^1^	2.6831 × 10^1^	2.6652 × 10^1^	2.6831 × 10^1^	2.6864 × 10^1^	2.6883 × 10^1^	2.6609 × 10^1^	2.6881 × 10^1^	2.6843 × 10^1^
		Std	7.5709 × 10^−2^	1.8703 × 10^−1^	4.7342 × 10^−2^	1.4799 × 10^−1^	9.5275 × 10^−3^	5.6289 × 10^−2^	8.6158 × 10^−2^	2.0722 × 10^−1^	6.9185 × 10^−2^	3.6745 × 10^−2^

**Table 13:** Ave and Std of all test images for FSIM in Otsu.

Image	Threshold	Metric	BHO	NDO	GJA	SSLO	LEE	SAO	RBMO	ESC	SBOA	MISBOA
baboon	4	Ave	8.3368 × 10^−1^	8.3309 × 10^−1^	8.3367 × 10^−1^	8.3259 × 10^−1^	8.3367 × 10^−1^	8.3367 × 10^−1^	8.3367 × 10^−1^	8.3340 × 10^−1^	8.3356 × 10^−1^	8.3356 × 10^−1^
		Std	9.9178 × 10^−5^	1.6870 × 10^−3^	2.2584 × 10^−16^	3.0753 × 10^−3^	2.2584 × 10^−16^	2.2584 × 10^−16^	2.2584 × 10^−16^	7.9317 × 10^−4^	6.0223 × 10^−4^	6.0223 × 10^−4^
	6	Ave	8.8549 × 10^−1^	inf	8.8557 × 10^−1^	8.8393 × 10^−1^	8.8462 × 10^−1^	8.8465 × 10^−1^	8.8474 × 10^−1^	8.8281 × 10^−1^	8.8538 × 10^−1^	8.8489 × 10^−1^
		Std	2.7147 × 10^−3^	inf	2.3959 × 10^−3^	6.3538 × 10^−3^	3.3876 × 10^−16^	1.2321 × 10^−3^	8.3541 × 10^−4^	6.3012 × 10^−3^	1.8339 × 10^−3^	9.6359 × 10^−4^
	8	Ave	9.2119 × 10^−1^	inf	9.2117 × 10^−1^	inf	9.2286 × 10^−1^	9.2133 × 10^−1^	9.1655 × 10^−1^	9.1388 × 10^−1^	9.2214 × 10^−1^	9.2129 × 10^−1^
		Std	6.4167 × 10^−3^	inf	3.6074 × 10^−3^	inf	6.2423 × 10^−4^	3.7032 × 10^−3^	1.9484 × 10^−2^	9.8909 × 10^−3^	4.5064 × 10^−3^	4.8059 × 10^−3^
	10	Ave	9.4242 × 10^−1^	inf	9.4213 × 10^−1^	inf	9.4324 × 10^−1^	9.3501 × 10^−1^	8.9844 × 10^−1^	9.3048 × 10^−1^	9.2100 × 10^−1^	9.4292 × 10^−1^
		Std	6.3689 × 10^−3^	inf	3.4091 × 10^−3^	inf	5.5858 × 10^−4^	4.1254 × 10^−2^	1.0871 × 10^−1^	9.2048 × 10^−3^	1.0052 × 10^−1^	3.5963 × 10^−3^
bank	4	Ave	8.3499 × 10^−1^	8.3504 × 10^−1^	8.3493 × 10^−1^	8.3505 × 10^−1^	8.3490 × 10^−1^	8.3487 × 10^−1^	8.3491 × 10^−1^	8.3499 × 10^−1^	8.3498 × 10^−1^	8.3493 × 10^−1^
		Std	2.2030 × 10^−4^	2.6605 × 10^−4^	1.2955 × 10^−4^	6.2546 × 10^−4^	1.1292 × 10^−16^	3.0615 × 10^−4^	2.0398 × 10^−5^	5.9176 × 10^−4^	2.1620 × 10^−4^	1.2955 × 10^−4^
	6	Ave	8.8128 × 10^−1^	8.8145 × 10^−1^	8.8145 × 10^−1^	8.8091 × 10^−1^	8.8128 × 10^−1^	8.8143 × 10^−1^	8.8139 × 10^−1^	8.8106 × 10^−1^	8.8119 × 10^−1^	8.8128 × 10^−1^
		Std	4.8838 × 10^−4^	7.1891 × 10^−4^	3.1737 × 10^−4^	1.5432 × 10^−3^	5.6460 × 10^−16^	2.7124 × 10^−4^	2.3995 × 10^−4^	1.0533 × 10^−3^	2.2669 × 10^−4^	5.6460 × 10^−16^
	8	Ave	9.0952 × 10^−1^	9.0904 × 10^−1^	9.0990 × 10^−1^	9.0831 × 10^−1^	9.0982 × 10^−1^	9.0964 × 10^−1^	9.0976 × 10^−1^	9.0835 × 10^−1^	9.0975 × 10^−1^	9.0970 × 10^−1^
		Std	1.5825 × 10^−3^	2.7873 × 10^−3^	8.3354 × 10^−4^	3.7422 × 10^−3^	1.2788 × 10^−4^	2.3413 × 10^−3^	1.3162 × 10^−3^	2.8426 × 10^−3^	1.2912 × 10^−3^	4.6018 × 10^−4^
	10	Ave	9.3119 × 10^−1^	9.3044 × 10^−1^	9.3116 × 10^−1^	9.2921 × 10^−1^	9.3127 × 10^−1^	9.3104 × 10^−1^	9.3129 × 10^−1^	9.2686 × 10^−1^	9.3114 × 10^−1^	9.3148 × 10^−1^
		Std	1.7641 × 10^−3^	2.6827 × 10^−3^	9.8791 × 10^−4^	3.4240 × 10^−3^	2.0227 × 10^−4^	2.0609 × 10^−3^	1.3813 × 10^−3^	2.6745 × 10^−3^	2.0855 × 10^−3^	1.0583 × 10^−3^
barbara	4	Ave	8.1307 × 10^−1^	8.1305 × 10^−1^	8.1307 × 10^−1^	8.1309 × 10^−1^	8.1307 × 10^−1^	8.1307 × 10^−1^	8.1307 × 10^−1^	8.1305 × 10^−1^	8.1307 × 10^−1^	8.1307 × 10^−1^
		Std	5.6460 × 10^−16^	1.2093 × 10^−4^	5.6460 × 10^−16^	4.6029 × 10^−4^	5.6460 × 10^−16^	5.6460 × 10^−16^	5.6460 × 10^−16^	2.5892 × 10^−4^	5.6460 × 10^−16^	5.6460 × 10^−16^
	6	Ave	8.6385 × 10^−1^	8.6414 × 10^−1^	8.6380 × 10^−1^	8.6318 × 10^−1^	8.6351 × 10^−1^	8.6360 × 10^−1^	8.6376 × 10^−1^	8.6354 × 10^−1^	8.6359 × 10^−1^	8.6351 × 10^−1^
		Std	3.9678 × 10^−4^	6.5980 × 10^−4^	2.6551 × 10^−4^	1.3910 × 10^−3^	5.6460 × 10^−16^	1.1160 × 10^−4^	3.9417 × 10^−4^	1.2120 × 10^−3^	1.5899 × 10^−4^	5.6460 × 10^−16^
	8	Ave	8.9112 × 10^−1^	8.9162 × 10^−1^	8.9132 × 10^−1^	8.9010 × 10^−1^	8.9151 × 10^−1^	8.9144 × 10^−1^	8.9153 × 10^−1^	8.8963 × 10^−1^	8.9132 × 10^−1^	8.9154 × 10^−1^
		Std	9.7223 × 10^−4^	1.0178 × 10^−3^	5.6094 × 10^−4^	1.5840 × 10^−3^	0.0000 × 10^00^	1.9333 × 10^−4^	4.9461 × 10^−4^	1.3220 × 10^−3^	3.9638 × 10^−4^	1.5889 × 10^−4^
	10	Ave	9.1120 × 10^−1^	9.1073 × 10^−1^	9.1185 × 10^−1^	9.0847 × 10^−1^	9.1231 × 10^−1^	9.1223 × 10^−1^	9.1152 × 10^−1^	9.0668 × 10^−1^	9.1186 × 10^−1^	9.1226 × 10^−1^
		Std	1.4046 × 10^−3^	1.8660 × 10^−3^	1.0481 × 10^−3^	2.1187 × 10^−3^	3.6625 × 10^−4^	5.5281 × 10^−4^	1.0763 × 10^−3^	2.6066 × 10^−3^	1.2123 × 10^−3^	6.4469 × 10^−4^
boat	4	Ave	8.1304 × 10^−1^	8.1305 × 10^−1^	8.1304 × 10^−1^	8.1282 × 10^−1^	8.1304 × 10^−1^	8.1304 × 10^−1^	8.1304 × 10^−1^	8.1297 × 10^−1^	8.1304 × 10^−1^	8.1304 × 10^−1^
		Std	3.7162 × 10^−6^	7.8733 × 10^−5^	3.3876 × 10^−16^	7.4146 × 10^−4^	3.3876 × 10^−16^	3.3876 × 10^−16^	3.3876 × 10^−16^	2.0443 × 10^−4^	3.3876 × 10^−16^	3.3876 × 10^−16^
	6	Ave	8.7865 × 10^−1^	8.7880 × 10^−1^	8.7874 × 10^−1^	8.7765 × 10^−1^	8.7886 × 10^−1^	8.7872 × 10^−1^	8.7877 × 10^−1^	8.7821 × 10^−1^	8.7881 × 10^−1^	8.7885 × 10^−1^
		Std	4.2906 × 10^−4^	3.5662 × 10^−4^	4.3243 × 10^−4^	1.4671 × 10^−3^	1.1292 × 10^−16^	2.1917 × 10^−4^	3.1326 × 10^−4^	9.3243 × 10^−4^	3.3064 × 10^−4^	2.0089 × 10^−4^
	8	Ave	9.1173 × 10^−1^	9.1137 × 10^−1^	9.1181 × 10^−1^	9.0959 × 10^−1^	9.1192 × 10^−1^	9.1199 × 10^−1^	9.1183 × 10^−1^	9.0859 × 10^−1^	9.1211 × 10^−1^	9.1180 × 10^−1^
		Std	1.0177 × 10^−3^	1.5367 × 10^−3^	6.1039 × 10^−4^	1.9458 × 10^−3^	8.6306 × 10^−6^	1.5156 × 10^−4^	9.0352 × 10^−4^	2.1600 × 10^−3^	7.7144 × 10^−4^	3.7235 × 10^−4^
	10	Ave	9.3275 × 10^−1^	9.3212 × 10^−1^	9.3230 × 10^−1^	9.2889 × 10^−1^	9.3348 × 10^−1^	9.3283 × 10^−1^	9.3257 × 10^−1^	9.2701 × 10^−1^	9.3286 × 10^−1^	9.3314 × 10^−1^
		Std	1.0256 × 10^−3^	1.7207 × 10^−3^	9.5327 × 10^−4^	2.1764 × 10^−3^	1.6442 × 10^−4^	8.8873 × 10^−4^	1.1292 × 10^−3^	3.2697 × 10^−3^	9.5286 × 10^−4^	6.5670 × 10^−4^
brain	4	Ave	6.7513 × 10^−1^	6.7505 × 10^−1^	6.7511 × 10^−1^	6.7510 × 10^−1^	6.7510 × 10^−1^	6.7511 × 10^−1^	6.7508 × 10^−1^	6.7514 × 10^−1^	6.7511 × 10^−1^	6.7512 × 10^−1^
		Std	8.7986 × 10^−5^	2.4925 × 10^−4^	8.0070 × 10^−5^	1.6006 × 10^−4^	8.4112 × 10^−5^	8.4774 × 10^−5^	8.5347 × 10^−5^	1.7712 × 10^−4^	8.0714 × 10^−5^	7.5690 × 10^−5^
	6	Ave	9.2039 × 10^−1^	7.6846 × 10^−1^	7.3908 × 10^−1^	9.1923 × 10^−1^	7.2396 × 10^−1^	7.9130 × 10^−1^	8.9728 × 10^−1^	8.6572 × 10^−1^	8.1456 × 10^−1^	8.6060 × 10^−1^
		Std	2.7556 × 10^−3^	1.0840 × 10^−1^	9.3017 × 10^−2^	3.0315 × 10^−3^	7.9135 × 10^−2^	1.1401 × 10^−1^	6.9108 × 10^−2^	9.7400 × 10^−2^	1.1568 × 10^−1^	1.0263 × 10^−1^
	8	Ave	9.4994 × 10^−1^	9.4187 × 10^−1^	9.5034 × 10^−1^	9.4804 × 10^−1^	9.5030 × 10^−1^	9.4202 × 10^−1^	9.5016 × 10^−1^	9.4651 × 10^−1^	9.5091 × 10^−1^	9.5043 × 10^−1^
		Std	1.6432 × 10^−3^	4.1456 × 10^−2^	1.5399 × 10^−3^	1.9501 × 10^−3^	1.2931 × 10^−3^	4.4793 × 10^−2^	1.6697 × 10^−3^	3.5200 × 10^−3^	1.4171 × 10^−3^	1.5326 × 10^−3^
	10	Ave	9.6396 × 10^−1^	9.6207 × 10^−1^	9.6353 × 10^−1^	9.6145 × 10^−1^	9.6367 × 10^−1^	9.6490 × 10^−1^	9.6397 × 10^−1^	9.6043 × 10^−1^	9.6430 × 10^−1^	9.6463 × 10^−1^
		Std	1.6824 × 10^−3^	3.0844 × 10^−3^	1.8047 × 10^−3^	2.2796 × 10^−3^	1.3262 × 10^−3^	1.4194 × 10^−3^	2.2229 × 10^−3^	2.8039 × 10^−3^	1.8369 × 10^−3^	1.9837 × 10^−3^
bridge	4	Ave	7.9571 × 10^−1^	7.9607 × 10^−1^	7.9569 × 10^−1^	7.9619 × 10^−1^	7.9569 × 10^−1^	7.9569 × 10^−1^	7.9569 × 10^−1^	7.9568 × 10^−1^	7.9569 × 10^−1^	7.9569 × 10^−1^
		Std	7.7060 × 10^−5^	1.0378 × 10^−3^	5.6460 × 10^−16^	1.4516 × 10^−3^	5.6460 × 10^−16^	5.6460 × 10^−16^	5.6460 × 10^−16^	1.6023 × 10^−3^	5.6460 × 10^−16^	5.6460 × 10^−16^
	6	Ave	8.7209 × 10^−1^	8.7230 × 10^−1^	8.7265 × 10^−1^	8.7283 × 10^−1^	8.7244 × 10^−1^	8.7236 × 10^−1^	8.7207 × 10^−1^	8.7016 × 10^−1^	8.7254 × 10^−1^	8.7242 × 10^−1^
		Std	1.3789 × 10^−3^	1.7030 × 10^−3^	7.8498 × 10^−4^	3.6708 × 10^−3^	4.5168 × 10^−16^	4.0512 × 10^−4^	6.5360 × 10^−4^	4.0374 × 10^−3^	5.9794 × 10^−4^	3.2904 × 10^−4^
	8	Ave	9.1221 × 10^−1^	9.1053 × 10^−1^	9.1258 × 10^−1^	9.0932 × 10^−1^	9.1258 × 10^−1^	9.1273 × 10^−1^	9.1221 × 10^−1^	9.0820 × 10^−1^	9.1260 × 10^−1^	9.1274 × 10^−1^
		Std	2.6828 × 10^−3^	3.2676 × 10^−3^	1.3492 × 10^−3^	7.0761 × 10^−3^	5.3245 × 10^−4^	7.8558 × 10^−4^	1.8129 × 10^−3^	6.6615 × 10^−3^	1.8963 × 10^−3^	1.1146 × 10^−3^
	10	Ave	9.3534 × 10^−1^	9.3413 × 10^−1^	9.3640 × 10^−1^	9.3453 × 10^−1^	9.3755 × 10^−1^	9.3666 × 10^−1^	9.3594 × 10^−1^	9.3006 × 10^−1^	9.3631 × 10^−1^	9.3722 × 10^−1^
		Std	2.7665 × 10^−3^	4.9611 × 10^−3^	1.3143 × 10^−3^	3.5396 × 10^−3^	1.0765 × 10^−3^	3.5689 × 10^−3^	1.9903 × 10^−3^	4.7943 × 10^−3^	2.7980 × 10^−3^	3.1010 × 10^−3^
camera	4	Ave	8.3308 × 10^−1^	8.3513 × 10^−1^	8.3661 × 10^−1^	8.3243 × 10^−1^	8.3541 × 10^−1^	8.3414 × 10^−1^	8.3279 × 10^−1^	8.3110 × 10^−1^	8.3421 × 10^−1^	8.3260 × 10^−1^
		Std	2.4931 × 10^−3^	4.6444 × 10^−3^	3.6630 × 10^−3^	2.2114 × 10^−3^	3.5382 × 10^−3^	8.1730 × 10^−3^	1.1035 × 10^−4^	3.2811 × 10^−3^	2.9369 × 10^−3^	7.6966 × 10^−4^
	6	Ave	8.7790 × 10^−1^	8.7797 × 10^−1^	8.7831 × 10^−1^	8.7547 × 10^−1^	8.7905 × 10^−1^	8.7879 × 10^−1^	8.7859 × 10^−1^	8.7712 × 10^−1^	8.7850 × 10^−1^	8.7868 × 10^−1^
		Std	1.0028 × 10^−3^	2.0275 × 10^−3^	8.4153 × 10^−4^	4.2740 × 10^−3^	3.3876 × 10^−16^	5.2412 × 10^−4^	6.2342 × 10^−4^	2.2453 × 10^−3^	7.5318 × 10^−4^	7.0857 × 10^−4^
	8	Ave	9.0161 × 10^−1^	9.0215 × 10^−1^	9.0248 × 10^−1^	9.0016 × 10^−1^	9.0351 × 10^−1^	9.0317 × 10^−1^	9.0252 × 10^−1^	8.9909 × 10^−1^	9.0306 × 10^−1^	9.0289 × 10^−1^
		Std	1.8965 × 10^−3^	1.0651 × 10^−3^	1.1945 × 10^−3^	4.7787 × 10^−3^	8.3352 × 10^−4^	9.5864 × 10^−4^	9.7287 × 10^−4^	4.1231 × 10^−3^	1.2348 × 10^−3^	7.8222 × 10^−4^
	10	Ave	9.1897 × 10^−1^	9.1751 × 10^−1^	9.1918 × 10^−1^	9.1737 × 10^−1^	9.1960 × 10^−1^	9.1943 × 10^−1^	9.1880 × 10^−1^	9.1442 × 10^−1^	9.1934 × 10^−1^	9.1907 × 10^−1^
		Std	1.3728 × 10^−3^	2.9766 × 10^−3^	1.1734 × 10^−3^	3.2815 × 10^−3^	6.9378 × 10^−4^	7.7839 × 10^−4^	1.3788 × 10^−3^	3.6976 × 10^−3^	8.6426 × 10^−4^	1.3506 × 10^−3^
cell	4	Ave	8.7881 × 10^−1^	8.7881 × 10^−1^	8.7881 × 10^−1^	8.7921 × 10^−1^	8.7881 × 10^−1^	8.7881 × 10^−1^	8.7881 × 10^−1^	8.7881 × 10^−1^	8.7881 × 10^−1^	8.7881 × 10^−1^
		Std	2.1679 × 10^−5^	0.0000 × 10^00^	0.0000 × 10^00^	8.9181 × 10^−4^	0.0000 × 10^00^	0.0000 × 10^00^	0.0000 × 10^00^	1.5601 × 10^−5^	0.0000 × 10^00^	0.0000 × 10^00^
	6	Ave	9.0001 × 10^−1^	9.0101 × 10^−1^	9.0162 × 10^−1^	9.0071 × 10^−1^	8.9979 × 10^−1^	9.0213 × 10^−1^	9.0137 × 10^−1^	9.0088 × 10^−1^	9.0020 × 10^−1^	8.9901 × 10^−1^
		Std	2.1633 × 10^−3^	3.2026 × 10^−3^	3.0904 × 10^−3^	3.6562 × 10^−3^	3.1560 × 10^−3^	2.7138 × 10^−3^	2.9883 × 10^−3^	2.5322 × 10^−3^	2.8643 × 10^−3^	2.0424 × 10^−3^
	8	Ave	9.1747 × 10^−1^	9.1613 × 10^−1^	9.1709 × 10^−1^	9.1369 × 10^−1^	9.1808 × 10^−1^	9.1790 × 10^−1^	9.1758 × 10^−1^	9.1568 × 10^−1^	9.1763 × 10^−1^	9.1776 × 10^−1^
		Std	1.2966 × 10^−3^	3.2993 × 10^−3^	1.7528 × 10^−3^	4.3344 × 10^−3^	1.7585 × 10^−4^	4.4744 × 10^−4^	8.4041 × 10^−4^	3.0147 × 10^−3^	1.0134 × 10^−3^	5.2775 × 10^−4^
	10	Ave	9.1943 × 10^−1^	9.1436 × 10^−1^	9.1785 × 10^−1^	9.1521 × 10^−1^	9.1806 × 10^−1^	9.1901 × 10^−1^	9.1959 × 10^−1^	9.1759 × 10^−1^	9.1853 × 10^−1^	9.1947 × 10^−1^
		Std	2.8304 × 10^−3^	8.9320 × 10^−3^	4.1635 × 10^−3^	7.8579 × 10^−3^	1.4524 × 10^−4^	2.2851 × 10^−3^	2.7134 × 10^−3^	4.4777 × 10^−3^	4.1297 × 10^−3^	3.2286 × 10^−3^
columbia	4	Ave	8.1554 × 10^−1^	8.1541 × 10^−1^	8.1554 × 10^−1^	8.1542 × 10^−1^	8.1554 × 10^−1^	8.1544 × 10^−1^	8.1550 × 10^−1^	8.1531 × 10^−1^	8.1554 × 10^−1^	8.1554 × 10^−1^
		Std	2.2584 × 10^−16^	6.5508 × 10^−4^	2.2584 × 10^−16^	6.8100 × 10^−4^	2.2584 × 10^−16^	2.3172 × 10^−4^	1.5510 × 10^−4^	4.9364 × 10^−4^	2.2584 × 10^−16^	2.2584 × 10^−16^
	6	Ave	8.6893 × 10^−1^	8.6875 × 10^−1^	8.6894 × 10^−1^	8.6893 × 10^−1^	8.6962 × 10^−1^	8.6883 × 10^−1^	8.6888 × 10^−1^	8.6732 × 10^−1^	8.6907 × 10^−1^	8.6956 × 10^−1^
		Std	8.0032 × 10^−4^	7.4952 × 10^−4^	6.9489 × 10^−4^	2.6061 × 10^−3^	4.5168 × 10^−16^	7.6014 × 10^−4^	7.7918 × 10^−4^	1.7120 × 10^−3^	6.5433 × 10^−4^	2.9061 × 10^−4^
	8	Ave	8.9769 × 10^−1^	8.9718 × 10^−1^	8.9763 × 10^−1^	8.9551 × 10^−1^	8.9759 × 10^−1^	8.9746 × 10^−1^	8.9764 × 10^−1^	8.9582 × 10^−1^	8.9758 × 10^−1^	8.9741 × 10^−1^
		Std	8.5901 × 10^−4^	2.8874 × 10^−3^	7.3566 × 10^−4^	2.2873 × 10^−3^	5.5705 × 10^−4^	6.2579 × 10^−4^	6.8143 × 10^−4^	2.3523 × 10^−3^	5.5947 × 10^−4^	5.5263 × 10^−4^
	10	Ave	9.1606 × 10^−1^	9.1617 × 10^−1^	9.1661 × 10^−1^	9.1494 × 10^−1^	9.1599 × 10^−1^	9.1625 × 10^−1^	9.1693 × 10^−1^	9.1112 × 10^−1^	9.1667 × 10^−1^	9.1597 × 10^−1^
		Std	1.5457 × 10^−3^	2.1005 × 10^−3^	1.4163 × 10^−3^	3.5849 × 10^−3^	4.5407 × 10^−4^	9.6993 × 10^−4^	9.8669 × 10^−4^	2.8319 × 10^−3^	9.5957 × 10^−4^	5.5185 × 10^−4^
couple	4	Ave	8.0086 × 10^−1^	8.0061 × 10^−1^	8.0091 × 10^−1^	8.0072 × 10^−1^	8.0091 × 10^−1^	8.0072 × 10^−1^	8.0085 × 10^−1^	8.0041 × 10^−1^	8.0072 × 10^−1^	8.0091 × 10^−1^
		Std	3.5224 × 10^−4^	7.8442 × 10^−4^	0.0000 × 10^00^	1.1877 × 10^−3^	0.0000 × 10^00^	3.4992 × 10^−4^	2.0637 × 10^−4^	1.5658 × 10^−3^	3.4992 × 10^−4^	0.0000 × 10^00^
	6	Ave	8.7275 × 10^−1^	8.7306 × 10^−1^	8.7320 × 10^−1^	8.7232 × 10^−1^	8.7307 × 10^−1^	8.7332 × 10^−1^	8.7319 × 10^−1^	8.7148 × 10^−1^	8.7310 × 10^−1^	8.7307 × 10^−1^
		Std	9.7153 × 10^−4^	5.9879 × 10^−4^	5.1333 × 10^−4^	1.8619 × 10^−3^	1.4990 × 10^−4^	4.3455 × 10^−4^	5.5403 × 10^−4^	2.2623 × 10^−3^	4.7986 × 10^−4^	1.4990 × 10^−4^
	8	Ave	9.1112 × 10^−1^	9.1130 × 10^−1^	9.1157 × 10^−1^	9.0910 × 10^−1^	9.1166 × 10^−1^	9.1164 × 10^−1^	9.1176 × 10^−1^	9.0953 × 10^−1^	9.1195 × 10^−1^	9.1195 × 10^−1^
		Std	1.4631 × 10^−3^	1.6446 × 10^−3^	8.0795 × 10^−4^	3.2240 × 10^−3^	3.3876 × 10^−16^	3.8122 × 10^−4^	8.4913 × 10^−4^	2.9322 × 10^−3^	7.6148 × 10^−4^	4.2149 × 10^−4^
	10	Ave	9.3244 × 10^−1^	9.3252 × 10^−1^	9.3369 × 10^−1^	9.3039 × 10^−1^	9.3357 × 10^−1^	9.3399 × 10^−1^	9.3384 × 10^−1^	9.2926 × 10^−1^	9.3423 × 10^−1^	9.3395 × 10^−1^
		Std	1.5894 × 10^−3^	3.2795 × 10^−3^	9.8854 × 10^−4^	2.5713 × 10^−3^	2.6843 × 10^−4^	4.3312 × 10^−4^	9.1003 × 10^−4^	3.1956 × 10^−3^	1.1932 × 10^−3^	7.3509 × 10^−4^

**Table 14:** Ave and Std of all test images for SSIM in Otsu.

Image	Threshold	Metric	BHO	NDO	GJA	SSLO	LEE	SAO	RBMO	ESC	SBOA	MISBOA
baboon	4	Ave	7.8185 × 10^−1^	7.8106 × 10^−1^	7.8186 × 10^−1^	7.8027 × 10^−1^	7.8186 × 10^−1^	7.8186 × 10^−1^	7.8186 × 10^−1^	7.8148 × 10^−1^	7.8174 × 10^−1^	7.8174 × 10^−1^
		Std	3.1523 × 10^−5^	2.6359 × 10^−3^	1.1292 × 10^−16^	3.9493 × 10^−3^	1.1292 × 10^−16^	1.1292 × 10^−16^	1.1292 × 10^−16^	1.1906 × 10^−3^	6.5272 × 10^−4^	6.5272 × 10^−4^
	6	Ave	8.5968 × 10^−1^	8.3046 × 10^−1^	8.5985 × 10^−1^	8.5722 × 10^−1^	8.5910 × 10^−1^	8.5901 × 10^−1^	8.5902 × 10^−1^	8.5686 × 10^−1^	8.5963 × 10^−1^	8.5930 × 10^−1^
		Std	2.4962 × 10^−3^	1.5693 × 10^−1^	2.1350 × 10^−3^	7.3448 × 10^−3^	5.6460 × 10^−16^	1.2366 × 10^−3^	9.3989 × 10^−4^	7.0364 × 10^−3^	1.6212 × 10^−3^	8.1059 × 10^−4^
	8	Ave	9.0503 × 10^−1^	6.6244 × 10^−1^	9.0515 × 10^−1^	7.2106 × 10^−1^	9.0706 × 10^−1^	9.0546 × 10^−1^	8.9841 × 10^−1^	8.9824 × 10^−1^	9.0612 × 10^−1^	9.0535 × 10^−1^
		Std	5.5109 × 10^−3^	4.0625 × 10^−1^	3.2042 × 10^−3^	3.6321 × 10^−1^	5.5322 × 10^−4^	3.7134 × 10^−3^	3.1993 × 10^−2^	8.2449 × 10^−3^	3.9570 × 10^−3^	4.2691 × 10^−3^
	10	Ave	9.3122 × 10^−1^	3.7263 × 10^−1^	9.3093 × 10^−1^	5.3090 × 10^−1^	9.3177 × 10^−1^	9.2144 × 10^−1^	8.7057 × 10^−1^	9.2021 × 10^−1^	9.0099 × 10^−1^	9.3149 × 10^−1^
		Std	4.9990 × 10^−3^	4.6402 × 10^−1^	3.0462 × 10^−3^	4.6242 × 10^−1^	7.7762 × 10^−4^	5.4565 × 10^−2^	1.4609 × 10^−1^	8.3495 × 10^−3^	1.3361 × 10^−1^	2.6996 × 10^−3^
bank	4	Ave	7.4549 × 10^−1^	7.4525 × 10^−1^	7.4554 × 10^−1^	7.4482 × 10^−1^	7.4554 × 10^−1^	7.4506 × 10^−1^	7.4551 × 10^−1^	7.4487 × 10^−1^	7.4550 × 10^−1^	7.4554 × 10^−1^
		Std	2.0501 × 10^−4^	6.1294 × 10^−4^	1.3683 × 10^−5^	9.4303 × 10^−4^	2.2584 × 10^−16^	1.4940 × 10^−3^	1.9667 × 10^−4^	1.5980 × 10^−3^	1.9658 × 10^−4^	1.3683 × 10^−5^
	6	Ave	8.1464 × 10^−1^	8.1541 × 10^−1^	8.1490 × 10^−1^	8.1409 × 10^−1^	8.1456 × 10^−1^	8.1494 × 10^−1^	8.1468 × 10^−1^	8.1395 × 10^−1^	8.1429 × 10^−1^	8.1456 × 10^−1^
		Std	1.0313 × 10^−3^	2.8049 × 10^−3^	7.2130 × 10^−4^	3.8865 × 10^−3^	1.1292 × 10^−16^	5.9255 × 10^−4^	4.8826 × 10^−4^	3.1549 × 10^−3^	6.6096 × 10^−4^	1.1292 × 10^−16^
	8	Ave	8.5602 × 10^−1^	8.5592 × 10^−1^	8.5631 × 10^−1^	8.5455 × 10^−1^	8.5621 × 10^−1^	8.5760 × 10^−1^	8.5708 × 10^−1^	8.5669 × 10^−1^	8.5699 × 10^−1^	8.5663 × 10^−1^
		Std	2.2378 × 10^−3^	4.9149 × 10^−3^	1.6701 × 10^−3^	6.1437 × 10^−3^	1.2856 × 10^−3^	3.4487 × 10^−3^	4.2876 × 10^−3^	5.3954 × 10^−3^	3.4577 × 10^−3^	1.8104 × 10^−3^
	10	Ave	8.8543 × 10^−1^	8.8429 × 10^−1^	8.8525 × 10^−1^	8.8227 × 10^−1^	8.8579 × 10^−1^	8.8572 × 10^−1^	8.8568 × 10^−1^	8.7981 × 10^−1^	8.8474 × 10^−1^	8.8595 × 10^−1^
		Std	2.3913 × 10^−3^	4.3030 × 10^−3^	1.5559 × 10^−3^	4.7059 × 10^−3^	3.4508 × 10^−4^	3.1034 × 10^−3^	2.9192 × 10^−3^	5.1919 × 10^−3^	4.3061 × 10^−3^	1.3422 × 10^−3^
barbara	4	Ave	6.5794 × 10^−1^	6.5793 × 10^−1^	6.5794 × 10^−1^	6.5800 × 10^−1^	6.5794 × 10^−1^	6.5794 × 10^−1^	6.5794 × 10^−1^	6.5797 × 10^−1^	6.5794 × 10^−1^	6.5794 × 10^−1^
		Std	0.0000 × 10^00^	5.9756 × 10^−5^	0.0000 × 10^00^	6.2162 × 10^−4^	0.0000 × 10^00^	0.0000 × 10^00^	0.0000 × 10^00^	5.4503 × 10^−4^	0.0000 × 10^00^	0.0000 × 10^00^
	6	Ave	7.3958 × 10^−1^	7.4054 × 10^−1^	7.3965 × 10^−1^	7.3843 × 10^−1^	7.3936 × 10^−1^	7.3945 × 10^−1^	7.3986 × 10^−1^	7.3887 × 10^−1^	7.3963 × 10^−1^	7.3936 × 10^−1^
		Std	1.2771 × 10^−3^	2.4713 × 10^−3^	9.2488 × 10^−4^	6.0913 × 10^−3^	5.6460 × 10^−16^	4.2145 × 10^−4^	8.4074 × 10^−4^	4.8124 × 10^−3^	7.4454 × 10^−4^	5.6460 × 10^−16^
	8	Ave	7.9937 × 10^−1^	8.0116 × 10^−1^	7.9966 × 10^−1^	7.9523 × 10^−1^	8.0132 × 10^−1^	8.0050 × 10^−1^	8.0136 × 10^−1^	7.9293 × 10^−1^	7.9969 × 10^−1^	8.0097 × 10^−1^
		Std	3.6565 × 10^−3^	7.0809 × 10^−3^	2.6070 × 10^−3^	9.6245 × 10^−3^	5.6460 × 10^−16^	1.7136 × 10^−3^	3.3587 × 10^−3^	8.5137 × 10^−3^	2.9981 × 10^−3^	1.2984 × 10^−3^
	10	Ave	8.3800 × 10^−1^	8.3735 × 10^−1^	8.3810 × 10^−1^	8.3542 × 10^−1^	8.3855 × 10^−1^	8.3839 × 10^−1^	8.3757 × 10^−1^	8.2864 × 10^−1^	8.3660 × 10^−1^	8.3870 × 10^−1^
		Std	5.3098 × 10^−3^	6.4311 × 10^−3^	4.3157 × 10^−3^	9.0808 × 10^−3^	1.5144 × 10^−3^	3.2208 × 10^−3^	5.4654 × 10^−3^	1.2515 × 10^−2^	6.9074 × 10^−3^	1.9804 × 10^−3^
boat	4	Ave	7.0743 × 10^−1^	7.0730 × 10^−1^	7.0743 × 10^−1^	7.0660 × 10^−1^	7.0743 × 10^−1^	7.0743 × 10^−1^	7.0743 × 10^−1^	7.0714 × 10^−1^	7.0743 × 10^−1^	7.0743 × 10^−1^
		Std	1.2738 × 10^−5^	5.2107 × 10^−4^	3.3876 × 10^−16^	1.8333 × 10^−3^	3.3876 × 10^−16^	3.3876 × 10^−16^	3.3876 × 10^−16^	6.6292 × 10^−4^	3.3876 × 10^−16^	3.3876 × 10^−16^
	6	Ave	8.0498 × 10^−1^	8.0527 × 10^−1^	8.0552 × 10^−1^	8.0286 × 10^−1^	8.0628 × 10^−1^	8.0525 × 10^−1^	8.0535 × 10^−1^	8.0416 × 10^−1^	8.0506 × 10^−1^	8.0609 × 10^−1^
		Std	1.9007 × 10^−3^	2.1358 × 10^−3^	1.1789 × 10^−3^	6.7234 × 10^−3^	5.6460 × 10^−16^	1.5983 × 10^−3^	1.7772 × 10^−3^	3.0217 × 10^−3^	1.3594 × 10^−3^	4.0263 × 10^−4^
	8	Ave	8.5846 × 10^−1^	8.5916 × 10^−1^	8.5930 × 10^−1^	8.5935 × 10^−1^	8.6035 × 10^−1^	8.5990 × 10^−1^	8.6039 × 10^−1^	8.5396 × 10^−1^	8.5976 × 10^−1^	8.5936 × 10^−1^
		Std	3.1528 × 10^−3^	4.7921 × 10^−3^	1.7430 × 10^−3^	8.3239 × 10^−3^	1.4461 × 10^−3^	1.6842 × 10^−3^	2.1554 × 10^−3^	8.4787 × 10^−3^	3.0585 × 10^−3^	1.6997 × 10^−3^
	10	Ave	8.9548 × 10^−1^	8.9411 × 10^−1^	8.9473 × 10^−1^	8.9241 × 10^−1^	8.9764 × 10^−1^	8.9724 × 10^−1^	8.9519 × 10^−1^	8.8677 × 10^−1^	8.9337 × 10^−1^	8.9528 × 10^−1^
		Std	5.6167 × 10^−3^	6.5833 × 10^−3^	3.2530 × 10^−3^	8.0224 × 10^−3^	7.2536 × 10^−4^	2.0843 × 10^−3^	3.0822 × 10^−3^	7.8028 × 10^−3^	5.2980 × 10^−3^	3.2774 × 10^−3^
brain	4	Ave	3.7684 × 10^−1^	3.7677 × 10^−1^	3.7674 × 10^−1^	3.7676 × 10^−1^	3.7673 × 10^−1^	3.7674 × 10^−1^	3.7669 × 10^−1^	3.7697 × 10^−1^	3.7668 × 10^−1^	3.7679 × 10^−1^
		Std	3.4561 × 10^−4^	3.3560 × 10^−4^	3.9361 × 10^−4^	5.0207 × 10^−4^	3.6593 × 10^−4^	3.5373 × 10^−4^	3.7657 × 10^−4^	4.5989 × 10^−4^	3.3902 × 10^−4^	2.9604 × 10^−4^
	6	Ave	6.5865 × 10^−1^	4.9226 × 10^−1^	4.5444 × 10^−1^	6.9139 × 10^−1^	4.3883 × 10^−1^	5.0737 × 10^−1^	6.1940 × 10^−1^	5.8723 × 10^−1^	5.4933 × 10^−1^	5.8427 × 10^−1^
		Std	5.4732 × 10^−2^	1.3098 × 10^−1^	1.0197 × 10^−1^	5.7404 × 10^−2^	8.8712 × 10^−2^	1.2349 × 10^−1^	8.7389 × 10^−2^	1.1123 × 10^−1^	1.4390 × 10^−1^	1.1513 × 10^−1^
	8	Ave	7.6612 × 10^−1^	7.2601 × 10^−1^	7.6325 × 10^−1^	7.4042 × 10^−1^	7.6843 × 10^−1^	7.4909 × 10^−1^	7.6147 × 10^−1^	6.9011 × 10^−1^	7.4929 × 10^−1^	7.6283 × 10^−1^
		Std	3.1178 × 10^−2^	7.8746 × 10^−2^	2.6351 × 10^−2^	5.4074 × 10^−2^	2.5880 × 10^−2^	6.7023 × 10^−2^	2.6416 × 10^−2^	6.5521 × 10^−2^	3.6423 × 10^−2^	3.1330 × 10^−2^
	10	Ave	7.6754 × 10^−1^	7.7545 × 10^−1^	7.7465 × 10^−1^	7.7925 × 10^−1^	7.7124 × 10^−1^	7.7659 × 10^−1^	7.8460 × 10^−1^	7.4420 × 10^−1^	7.7966 × 10^−1^	7.9650 × 10^−1^
		Std	2.8464 × 10^−2^	5.0717 × 10^−2^	2.9104 × 10^−2^	5.6951 × 10^−2^	2.6333 × 10^−2^	3.0514 × 10^−2^	3.1401 × 10^−2^	6.2877 × 10^−2^	3.1633 × 10^−2^	3.6746 × 10^−2^
bridge	4	Ave	6.9311 × 10^−1^	6.9389 × 10^−1^	6.9311 × 10^−1^	6.9384 × 10^−1^	6.9311 × 10^−1^	6.9311 × 10^−1^	6.9311 × 10^−1^	6.9333 × 10^−1^	6.9311 × 10^−1^	6.9311 × 10^−1^
		Std	6.8714 × 10^−6^	2.1949 × 10^−3^	1.1292 × 10^−16^	2.9091 × 10^−3^	1.1292 × 10^−16^	1.1292 × 10^−16^	1.1292 × 10^−16^	3.1094 × 10^−3^	1.1292 × 10^−16^	1.1292 × 10^−16^
	6	Ave	8.1543 × 10^−1^	8.1598 × 10^−1^	8.1564 × 10^−1^	8.1535 × 10^−1^	8.1527 × 10^−1^	8.1480 × 10^−1^	8.1499 × 10^−1^	8.1325 × 10^−1^	8.1539 × 10^−1^	8.1521 × 10^−1^
		Std	2.7597 × 10^−3^	4.9376 × 10^−3^	1.1678 × 10^−3^	7.7628 × 10^−3^	3.3876 × 10^−16^	9.5752 × 10^−4^	1.8773 × 10^−3^	8.5453 × 10^−3^	1.1698 × 10^−3^	8.0551 × 10^−4^
	8	Ave	8.7742 × 10^−1^	8.7462 × 10^−1^	8.7817 × 10^−1^	8.7393 × 10^−1^	8.7827 × 10^−1^	8.7813 × 10^−1^	8.7677 × 10^−1^	8.7333 × 10^−1^	8.7760 × 10^−1^	8.7826 × 10^−1^
		Std	3.8828 × 10^−3^	6.8789 × 10^−3^	2.2062 × 10^−3^	1.1602 × 10^−2^	1.2072 × 10^−3^	1.9286 × 10^−3^	4.5428 × 10^−3^	1.0762 × 10^−2^	3.7647 × 10^−3^	2.0696 × 10^−3^
	10	Ave	9.1302 × 10^−1^	9.1079 × 10^−1^	9.1343 × 10^−1^	9.1276 × 10^−1^	9.1556 × 10^−1^	9.1558 × 10^−1^	9.1392 × 10^−1^	9.0773 × 10^−1^	9.1386 × 10^−1^	9.1584 × 10^−1^
		Std	5.8384 × 10^−3^	9.4939 × 10^−3^	4.2564 × 10^−3^	6.8804 × 10^−3^	2.4738 × 10^−3^	7.6182 × 10^−3^	6.4535 × 10^−3^	9.3464 × 10^−3^	7.2528 × 10^−3^	5.3954 × 10^−3^
camera	4	Ave	7.5084 × 10^−1^	7.2713 × 10^−1^	7.2125 × 10^−1^	7.5844 × 10^−1^	7.3304 × 10^−1^	6.9808 × 10^−1^	7.5913 × 10^−1^	7.5152 × 10^−1^	7.4483 × 10^−1^	7.5847 × 10^−1^
		Std	2.1361 × 10^−2^	3.7741 × 10^−2^	3.5908 × 10^−2^	5.1520 × 10^−3^	3.4685 × 10^−2^	3.6565 × 10^−2^	5.3832 × 10^−4^	1.3903 × 10^−2^	2.8790 × 10^−2^	2.0595 × 10^−3^
	6	Ave	8.0320 × 10^−1^	8.0354 × 10^−1^	8.0382 × 10^−1^	7.9953 × 10^−1^	8.0543 × 10^−1^	8.0490 × 10^−1^	8.0455 × 10^−1^	8.0132 × 10^−1^	8.0421 × 10^−1^	8.0456 × 10^−1^
		Std	2.6642 × 10^−3^	4.3932 × 10^−3^	2.0994 × 10^−3^	1.0309 × 10^−2^	1.1292 × 10^−16^	1.0898 × 10^−3^	1.3317 × 10^−3^	5.3853 × 10^−3^	1.7152 × 10^−3^	1.6905 × 10^−3^
	8	Ave	8.3094 × 10^−1^	8.3263 × 10^−1^	8.3453 × 10^−1^	8.2704 × 10^−1^	8.3668 × 10^−1^	8.3532 × 10^−1^	8.3372 × 10^−1^	8.2422 × 10^−1^	8.3549 × 10^−1^	8.3463 × 10^−1^
		Std	5.0436 × 10^−3^	4.5118 × 10^−3^	3.8335 × 10^−3^	1.2769 × 10^−2^	3.1533 × 10^−3^	3.2502 × 10^−3^	3.8801 × 10^−3^	1.2113 × 10^−2^	4.6657 × 10^−3^	3.5981 × 10^−3^
	10	Ave	8.6095 × 10^−1^	8.5745 × 10^−1^	8.6033 × 10^−1^	8.5849 × 10^−1^	8.6226 × 10^−1^	8.6026 × 10^−1^	8.5886 × 10^−1^	8.4937 × 10^−1^	8.6065 × 10^−1^	8.5987 × 10^−1^
		Std	6.0346 × 10^−3^	9.8191 × 10^−3^	5.7288 × 10^−3^	1.1529 × 10^−2^	3.4242 × 10^−3^	4.2943 × 10^−3^	6.3991 × 10^−3^	1.3199 × 10^−2^	6.1487 × 10^−3^	5.4403 × 10^−3^
cell	4	Ave	7.2796 × 10^−1^	7.2795 × 10^−1^	7.2795 × 10^−1^	7.2856 × 10^−1^	7.2795 × 10^−1^	7.2795 × 10^−1^	7.2795 × 10^−1^	7.2795 × 10^−1^	7.2795 × 10^−1^	7.2795 × 10^−1^
		Std	3.1833 × 10^−5^	2.2584 × 10^−16^	2.2584 × 10^−16^	9.9055 × 10^−4^	2.2584 × 10^−16^	2.2584 × 10^−16^	2.2584 × 10^−16^	2.2908 × 10^−5^	2.2584 × 10^−16^	2.2584 × 10^−16^
	6	Ave	7.3140 × 10^−1^	7.2937 × 10^−1^	7.2630 × 10^−1^	7.3443 × 10^−1^	7.3062 × 10^−1^	7.2351 × 10^−1^	7.2319 × 10^−1^	7.2436 × 10^−1^	7.2283 × 10^−1^	7.2592 × 10^−1^
		Std	2.0467 × 10^−2^	1.9922 × 10^−2^	2.1215 × 10^−2^	1.9707 × 10^−2^	1.6421 × 10^−2^	2.1134 × 10^−2^	2.2424 × 10^−2^	2.4677 × 10^−2^	2.1467 × 10^−2^	2.0974 × 10^−2^
	8	Ave	7.7368 × 10^−1^	7.7089 × 10^−1^	7.7527 × 10^−1^	7.6453 × 10^−1^	7.7464 × 10^−1^	7.7258 × 10^−1^	7.7239 × 10^−1^	7.7089 × 10^−1^	7.7513 × 10^−1^	7.7495 × 10^−1^
		Std	9.7507 × 10^−3^	1.2523 × 10^−2^	5.4677 × 10^−3^	1.5869 × 10^−2^	7.6730 × 10^−4^	7.8557 × 10^−3^	9.2329 × 10^−3^	1.4845 × 10^−2^	4.7834 × 10^−3^	2.6755 × 10^−3^
	10	Ave	8.0444 × 10^−1^	8.0296 × 10^−1^	8.0596 × 10^−1^	8.0411 × 10^−1^	8.1015 × 10^−1^	8.0610 × 10^−1^	8.0479 × 10^−1^	7.9724 × 10^−1^	8.0680 × 10^−1^	8.0915 × 10^−1^
		Std	6.8750 × 10^−3^	9.3244 × 10^−3^	7.9107 × 10^−3^	1.0840 × 10^−2^	8.0015 × 10^−5^	7.2430 × 10^−3^	6.7964 × 10^−3^	1.0737 × 10^−2^	6.5355 × 10^−3^	3.5475 × 10^−3^
columbia	4	Ave	7.1971 × 10^−1^	7.1987 × 10^−1^	7.1971 × 10^−1^	7.1955 × 10^−1^	7.1971 × 10^−1^	7.1978 × 10^−1^	7.1974 × 10^−1^	7.1921 × 10^−1^	7.1971 × 10^−1^	7.1971 × 10^−1^
		Std	1.1292 × 10^−16^	2.4193 × 10^−3^	1.1292 × 10^−16^	3.6586 × 10^−3^	1.1292 × 10^−16^	1.6996 × 10^−4^	1.1376 × 10^−4^	2.2743 × 10^−3^	1.1292 × 10^−16^	1.1292 × 10^−16^
	6	Ave	7.9306 × 10^−1^	7.9332 × 10^−1^	7.9348 × 10^−1^	7.9570 × 10^−1^	7.9375 × 10^−1^	7.9431 × 10^−1^	7.9372 × 10^−1^	7.9030 × 10^−1^	7.9359 × 10^−1^	7.9377 × 10^−1^
		Std	1.8474 × 10^−3^	4.4889 × 10^−3^	1.5345 × 10^−3^	8.1408 × 10^−3^	3.3876 × 10^−16^	1.4197 × 10^−3^	1.8446 × 10^−3^	6.3202 × 10^−3^	2.1375 × 10^−3^	8.2384 × 10^−5^
	8	Ave	8.3752 × 10^−1^	8.3449 × 10^−1^	8.3709 × 10^−1^	8.3449 × 10^−1^	8.3738 × 10^−1^	8.3658 × 10^−1^	8.3666 × 10^−1^	8.3439 × 10^−1^	8.3669 × 10^−1^	8.3766 × 10^−1^
		Std	2.9757 × 10^−3^	7.5153 × 10^−3^	2.0112 × 10^−3^	8.6325 × 10^−3^	1.1565 × 10^−3^	2.4140 × 10^−3^	3.4237 × 10^−3^	7.3768 × 10^−3^	2.7579 × 10^−3^	1.1175 × 10^−3^
	10	Ave	8.6142 × 10^−1^	8.6015 × 10^−1^	8.6045 × 10^−1^	8.6532 × 10^−1^	8.5949 × 10^−1^	8.6136 × 10^−1^	8.5906 × 10^−1^	8.5420 × 10^−1^	8.6038 × 10^−1^	8.6105 × 10^−1^
		Std	4.8179 × 10^−3^	6.0141 × 10^−3^	4.1734 × 10^−3^	1.2912 × 10^−2^	9.3868 × 10^−4^	4.9533 × 10^−3^	3.7042 × 10^−3^	6.3466 × 10^−3^	5.2010 × 10^−3^	2.7178 × 10^−3^
couple	4	Ave	7.2971 × 10^−1^	7.2885 × 10^−1^	7.2991 × 10^−1^	7.2893 × 10^−1^	7.2991 × 10^−1^	7.2918 × 10^−1^	7.2970 × 10^−1^	7.2893 × 10^−1^	7.2918 × 10^−1^	7.2991 × 10^−1^
		Std	1.2007 × 10^−3^	2.5780 × 10^−3^	3.3876 × 10^−16^	2.8759 × 10^−3^	3.3876 × 10^−16^	1.3475 × 10^−3^	7.9470 × 10^−4^	3.3411 × 10^−3^	1.3475 × 10^−3^	3.3876 × 10^−16^
	6	Ave	8.3226 × 10^−1^	8.3280 × 10^−1^	8.3314 × 10^−1^	8.3099 × 10^−1^	8.3313 × 10^−1^	8.3343 × 10^−1^	8.3323 × 10^−1^	8.3053 × 10^−1^	8.3319 × 10^−1^	8.3313 × 10^−1^
		Std	1.5334 × 10^−3^	1.2932 × 10^−3^	7.4539 × 10^−4^	4.3889 × 10^−3^	2.0419 × 10^−4^	7.3568 × 10^−4^	9.8343 × 10^−4^	4.5662 × 10^−3^	6.1716 × 10^−4^	2.0419 × 10^−4^
	8	Ave	8.7659 × 10^−1^	8.7639 × 10^−1^	8.7699 × 10^−1^	8.7215 × 10^−1^	8.7783 × 10^−1^	8.7766 × 10^−1^	8.7734 × 10^−1^	8.7503 × 10^−1^	8.7760 × 10^−1^	8.7778 × 10^−1^
		Std	1.5501 × 10^−3^	3.5710 × 10^−3^	7.5826 × 10^−4^	4.7311 × 10^−3^	3.3876 × 10^−16^	5.6560 × 10^−4^	8.6225 × 10^−4^	3.8435 × 10^−3^	5.0269 × 10^−4^	3.0561 × 10^−4^
	10	Ave	9.0309 × 10^−1^	9.0306 × 10^−1^	9.0372 × 10^−1^	9.0017 × 10^−1^	9.0398 × 10^−1^	9.0429 × 10^−1^	9.0488 × 10^−1^	9.0000 × 10^−1^	9.0460 × 10^−1^	9.0378 × 10^−1^
		Std	1.6416 × 10^−3^	3.7762 × 10^−3^	7.0856 × 10^−4^	3.5116 × 10^−3^	1.6428 × 10^−4^	9.0869 × 10^−4^	1.5499 × 10^−3^	4.1125 × 10^−3^	1.4444 × 10^−3^	4.4981 × 10^−4^

**Table 15:** Comparison of running times of various algorithms.

Image/Algorithm	GJA	SSLO	ESC	SBOA	MISBOA
baboon	26.09	22.05	40.74	22.71	23.77
bank	26.20	24.49	40.80	22.15	22.85
barbara	26.50	24.78	41.32	22.46	23.05
boat	26.89	24.99	41.95	22.72	23.51
brain	26.19	24.54	40.88	22.21	22.90
bridge	44.81	41.15	73.94	36.27	38.05
camera	26.12	24.41	40.72	22.07	22.68
cell	84.37	76.88	152.04	65.68	69.91
columbia	26.26	24.52	40.93	22.14	22.82
couple	26.82	25.04	41.45	22.73	23.47

## Data Availability

The original contributions presented in this study are included in the article. Further inquiries can be directed to the corresponding author.
